# Cardio-metabolic-related plasma proteins reveal biological links between cardiovascular diseases and fragility fractures: a cohort and Mendelian randomisation investigation

**DOI:** 10.1016/j.ebiom.2025.105580

**Published:** 2025-02-06

**Authors:** Karl Michaëlsson, Rui Zheng, John A. Baron, Tove Fall, Alicja Wolk, Lars Lind, Jonas Höijer, Carl Brunius, Eva Warensjö Lemming, Olga E. Titova, Bodil Svennblad, Susanna C. Larsson, Shuai Yuan, Håkan Melhus, Liisa Byberg, Hannah L. Brooke

**Affiliations:** a Medical Epidemiology, Department of Surgical Sciences, Uppsala University, Uppsala, Sweden; b Clinical Epidemiology, Department of Medical Sciences, Uppsala University, Uppsala, Sweden; c Department of Epidemiology, Gillings School of Global Public Health, University of North Carolina, Chapel Hill, NC, USA; d Molecular Epidemiology, Department of Medical Sciences, Uppsala University, Uppsala, Sweden; e Unit of Cardiovascular and Nutritional Epidemiology, Institute of Environmental Medicine, Karolinska Institutet, Stockholm, Sweden; f Food and Nutrition Science, Department of Life Sciences, Chalmers University of Technology, Gothenburg, Sweden; g Clinical Pharmacology, Department of Medical Sciences, Uppsala University, Uppsala, Sweden

**Keywords:** Cardiovascular diseases, Fragility fractures, Cohort studies, Mendelian randomisation, Proteomics

## Abstract

**Background:**

How cardiovascular diseases (CVD) predispose to a higher risk of fragility fractures is not well understood. Both contribute to significant components of disease burden and health expenditure. Poor bone quality, central obesity, sarcopenia, falls, and low grip strength are independent risk factors for hip and other fragility fractures and also for CVD and early death.

**Methods:**

We used proteomics and a cohort design combined with Mendelian randomisation analysis to understand shared mechanisms for developing CVD and fragility fractures, two significant sources of disease burden and health expenditure. We primarily aimed to discover and replicate the association of 274 cardio-metabolic-related proteins with future rates of hip and any fracture in two separate population-based cohorts, with a total of 12,314 women and men.

**Findings:**

The average age at baseline was 68 years in the discovery cohort of women and 74 years in the mixed-sex replication cohort. During 100,619 person-years of follow-up, 2168 had any fracture, and 538 had a hip fracture. Our analysis resulted in 24 cardiometabolic proteins associated with fracture risk: 20 with hip fracture, 9 with any fracture, and 5 with both. The associations remained even if protein concentrations were measured from specimens taken during preclinical stages of cardio-metabolic diseases, and 19 associations remained after adjustment for bone mineral density. Twenty-two of the proteins were associated with total body fat mass or lean body mass. Mendelian randomisation (MR) analysis supported causality since genetically predicted levels of SOST (Sclerostin), CCDC80 (Coiled-coil domain-containing protein 80), NT-proBNP (N-terminal prohormone brain natriuretic peptide), and BNP (Brain natriuretic peptide) were associated with risk of hip fracture. MR analysis also revealed a possible negative impact on bone mineral density (BMD) by genetically predicted higher levels of SOST, CCDC80, and TIMP4 (Metalloproteinase inhibitor 4). The MR association with BMD was positive for PTX3 (Pentraxin-related protein) and SPP1 (Osteopontin). Genetically predicted higher concentrations of SOST and lower concentrations of SPP1 also conferred a higher risk of falls and lowered grip strength. The genetically determined concentration of nine proteins influenced fat mass, and one influenced lean body mass.

**Interpretation:**

These data reveal biological links between cardiovascular diseases and fragility fractures. The proteins should be further evaluated as shared targets for developing pharmacological interventions to prevent fractures and cardiovascular disease.

**Funding:**

The study was supported by funding from the 10.13039/501100004359Swedish Research Council (https://www.vr.se; grants No. 2015-03257, 2017-00644, 2017-06100, and 2019-01291 to Karl Michaëlsson) and funding from 10.13039/501100004200Olle Engkvist Byggmästares stiftelse (SOEB).

**Research in context**

**Evidence before this study**

CVDs are associated with a higher risk of hip fracture and other types of fragility fractures, independently of recognised clinical risk factors. Conversely, low bone mineral density (BMD) and increased bone resorption, prior fragility fractures, sarcopenia, and low grip strength have been observationally associated with higher rates of cardiovascular events, independently of established cardiovascular risk factors. Co-twin control analyses in identical twins indicate that common genes may predispose to the development of both CVD and fractures. Despite this solid indication of links between CVD and fragility fracture, there is little understanding of the underlying shared mechanisms.

**Added value of this study**

We identified that 24 firmly established cardio-metabolic-related proteins were also associated with fracture risk in older women and men, even after considering prevalent cardiovascular disease at baseline. A strong indication of a direct causal link to hip fracture was established for four of the 24 proteins using MR analysis, which also showed that two of these and 7 additional proteins were also associated with secondary outcomes that are known to be involved in mechanisms that impact fracture risk. Hence, these proteins reveal biological links between CVD and fragility fractures. However, indirectly, the other 13 fracture-related proteins could still be causally related to fracture-developing pathogenic mechanisms.

**Implications of all the available evidence**

Our findings provide a common mechanistic bridge between CVD and fractures. Additional research is required to further corroborate our results; further validation of the identified proteins as targets for pharmacological interventions is necessary to realise the clinical implications of our results.

## Introduction

Cardiovascular diseases (CVDs) and fragility fractures, both leading sources of mortality and morbidity in the Western world,^1^ affect overlapping populations. CVDs are associated with a higher risk of hip fracture and other types of fragility fractures, independently of recognised clinical risk factors.^2^, ^3^, ^4^, ^5^, ^6^ Conversely, low bone mineral density (BMD) and increased bone resorption, prior fragility fractures, sarcopenia, and low grip strength have been observationally associated with higher rates of cardiovascular events, independently of established cardiovascular risk factors.^7^, ^8^, ^9^, ^10^, ^11^, ^12^, ^13^ Co-twin control analyses in identical twins indicate that common genes may predispose to the development of both CVD and fractures.^2^

Despite this solid indication of links between CVD and fragility fracture, there is little understanding of the underlying shared mechanisms. Circulating proteins act as essential keys in disease-development pathways, and proteomics offers the potential to discover the joint molecular underpinnings of CVD and fragility fractures. Proteomic discovery and replication studies of fracture do not exist.

In two population-based cohorts, we investigated the associations of 274 circulating cardiovascular- and metabolism-related protein biomarkers with the rates of hip and other types of fractures to discover shared common pathogenic mechanisms for cardiovascular diseases and fractures. We elucidate whether these proteins are associated with major risk factors for fracture, including BMD, bone size, bone turnover markers, body fat, and lean muscle mass.^14^, ^15^, ^16^, ^17^, ^18^ To strengthen evidence of causality, we used Mendelian randomisation (MR) analyses^19^ to evaluate replicated associations of proteins with hip fracture, BMD, body composition, falls, and grip strength (as a measure of muscle function) as outcomes.

## Methods

### Study cohorts

**Fig. 1:**
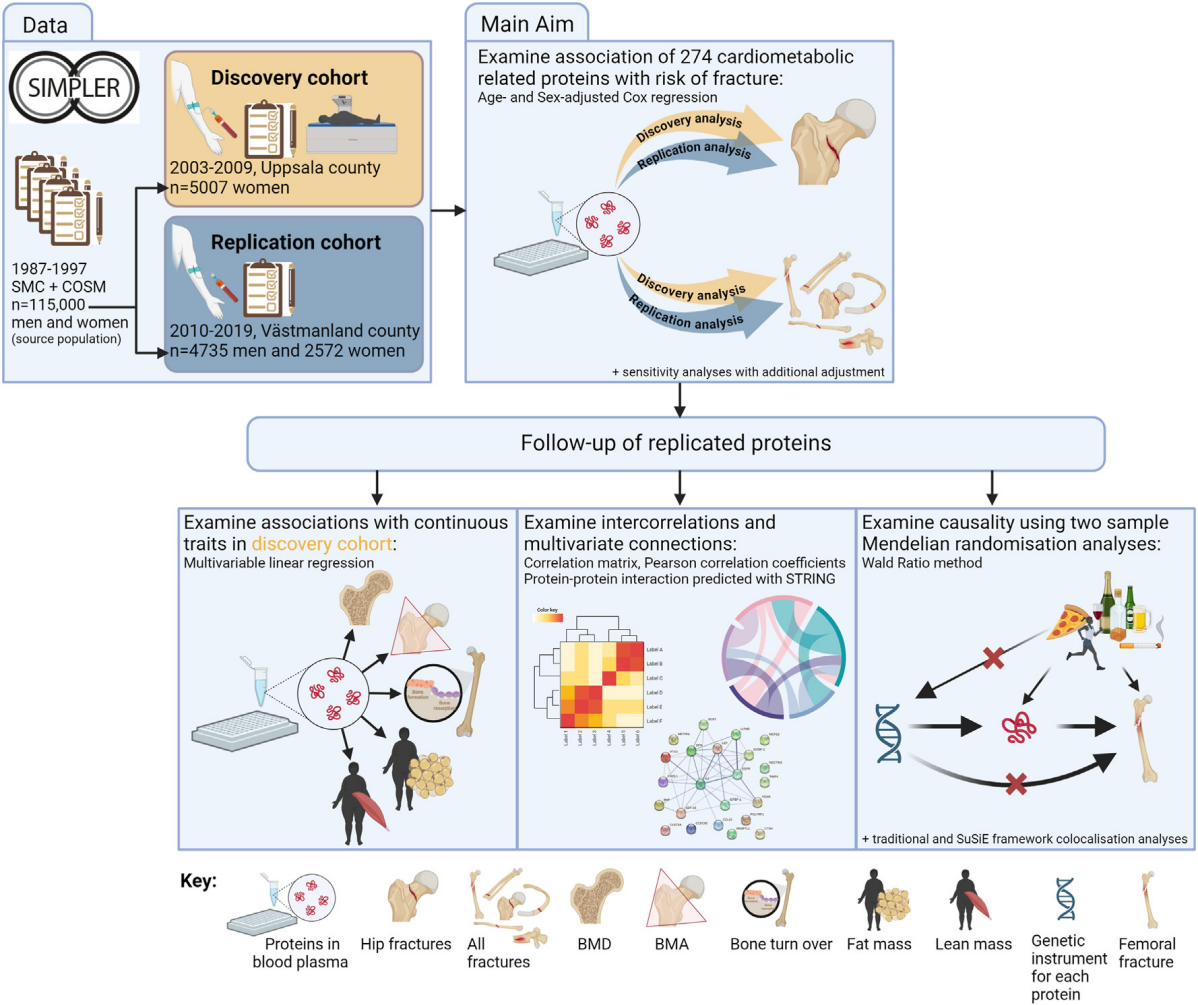
Overview of study design showing data, main aim, follow-up of replicated proteins and main analytical approaches. SIMPLER: Swedish Infrastructure for Medical Population-Based Life-Course and Environmental Research; SMC: Swedish Mammography Cohort; COSM: Cohort of Swedish Men; STRING: Search Tool for the Retrieval of Interacting Genes/Proteins; SuSiE: Sum of Single Effects; BMD: Bone Mineral Density; BMA: Bone Mineral Area. Created with BioRender.com.

To identify cardio-metabolic proteins associated with hip fractures, we used two cohort analyses in a discovery/replication approach. The two cohorts were randomly selected from the Swedish Mammography Cohort (SMC) and the Cohort of Swedish Men (COSM), both part of the Swedish Infrastructure for Medical Population-Based Life-Course and Environmental Research (SIMPLER; https://www.simpler4health.se/) (Fig. 1 displays the outline of the study design).

The discovery cohort included 5007 women (born 1920–1944, 65% of those invited) from Uppsala County, who provided fasting biosamples, underwent physical examinations and dual-energy X-ray absorptiometry (DXA), and had height and weight measurements taken between November 2003 and October 2009.

The replication cohort included 7307 individuals (4735 men and 2572 women, born 1920–1952; 85% of those invited) from neighbouring Västmanland County. Between May 2010 and June 2019, participants provided the same fasting biosamples and physical examinations as the discovery cohort and had height and weight measurements taken but did not undergo DXA examination.

Participants in the discovery and replication cohort responded to lifestyle, food frequency, and health questionnaires one month before their examination. The women and men also responded to lifestyle, food frequency, and health questionnaires in 1987–1990 and 1997.

### Plasma collection and proteomics measurement

Blood samples were collected in the morning following an overnight fast. For lithium-heparin plasma preparation, blood samples were light-protected and, after a delay of 15–20 min at room temperature, were spun in a centrifuge at 1615 g for 11 min at 4 °C. The plasma was aliquoted and stored at −80 °C until analysis.

We used the proximity extension assay (PEA) technique to measure levels of proteins in lithium-heparin plasma.^20^ Each protein is addressed by a matched pair of antibodies, coupled to unique, partially complementary oligonucleotides, and measured by quantitative real-time PCR. Only correctly matched antibody pairs give rise to a signal, yielding a high specificity.^20^^,^^21^ The mean intra- and inter-assay coefficients of variation were 8% and 12%. Samples were analysed in 2017–2019 at the Clinical Biomarkers Facility, SciLifeLab, Uppsala University using the Proseek Multiplex CVD II, CVD III, and Metabolism panels that cover proteins involved in cardiovascular disease, metabolism, and immune response processes; in total, 276 proteins were analysed (Supplementary Table S1). We used bridging samples from the two cohorts to standardise concentrations. Data generation consisted of normalisation of matched counts of an assay to the extension control spiked into every sample plate, log-2 transformation of the data, and level adjustment using the plate control. Normalised Protein eXpression (NPX) is Olink's relative quantification unit on a log-2 scale. A one-unit higher NPX represents an approximate doubling of measured protein concentration. Two panels measured one protein (NT-proBNP); only concentrations analysed by the Metabolism panel were retained for analysis. Reported plasma concentrations below LOD (lower limit of detection) were retained as such. One protein (CCL22) in the original CVD III panel used in the discovery cohort analysis was later replaced by Olink with the protein GP6 in the replication cohort analysis. Neither of these proteins was used in the present project. Less than 3% of data points were missing, and no protein was deleted; therefore, we had data for 274 proteins among 12,314 participants.

### Fracture identification—primary outcomes

We obtained information on all incident fracture events through 2020 using the unique Swedish personal registration number and linkage to the National In- and Out-Patient Registries (NPR), using ICD10 codes S720-S722 for hip fracture and S12-S92 for any fracture. Uppsala County has completed reporting to the National In-Patient Register since 1964, Västmanland since 1973, and the register has been nationally complete since 1987. The Out-Patient Register has had full national coverage since 2001. Reporting is mandatory by law for all physicians and hospitals, both private and publicly funded, to deliver complete data to the NPR.^22^ The positive predictive value for a first diagnosis of hip fracture in the NPR has been estimated from 95% to 99%, depending on the study, while the negative predictive value has been calculated to be 100%.^22^, ^23^, ^24^ The coding accuracies in the NPR for other types of extremity fractures are approximately 95%.^25^ We used a previously described validated algorithm^26^ to distinguish incident fractures from readmissions; only first fracture events after baseline were used in the analysis. We retained all non-pathological fractures in the analysis since comparable increases in the risks of low- and high-impact trauma fractures have been seen in association with decreasing BMD.^27^

### Body composition measurements and bone turnover markers—secondary outcomes

All participants had their height and weight measured by a stadiometer and a scale, respectively. Total body fat mass (FM) and lean mass (LM) in the discovery cohort were measured by DXA (Lunar Prodigy, Lunar Corp, Madison, WI, USA). We calculated total body fat mass index (FMI), lean mass index (LMI), and body mass index (BMI) by dividing FM, LM, and weight by height squared (kg/m^2^), respectively. Using the same DXA equipment, we also measured areal bone mineral density (BMD) and bone mineral area (BMA) at the total dual hip, lumbar spine L1-L4, and total body (g/cm^2^). Our narrow fan-beam DXA scanner provides minimal bone magnification errors.^28^^,^^29^ Modest differences in the area can render significant differences in bone strength and fracture risk, proportional to the fourth power of the bone radius.^30^, ^31^, ^32^, ^33^, ^34^ The precision error of triple DXA scans on 15 individuals, including repositioning, was 0.8–1.5%, depending on the type of measurement (regional or total BMD, LM, or FM). The fat mass measured by our narrow fan beam DXA equipment is 1.7–2% higher than that estimated by a four-compartment model.^35^

Fasting serum concentrations of CrossLaps and osteocalcin were analysed on a Roche Cobas 8000 e602 module (Roche Diagnostics, Mannheim, Germany) using the β-CrossLaps and N-MID Osteocalcin reagent kits. The coefficient of variation (CV) for CrossLaps was 1.8% at 294 ng/L and 1.4% at 2869 ng/L. CV for osteocalcin was 1% at 184 μg/L and 1% at 201 μg/L.

### Additional personal, lifestyle, and health status assessment

We collected information from the questionnaire examination one month before sample collection, including smoking habits, leisure-time physical activity, education, marital status, postmenopausal oestrogen therapy, bisphosphonate use, vitamin D and calcium supplement use, menopausal status, and parity. We calculated nutrient intakes as cumulative averages from food frequency questionnaire responses in 1987–1990, 1997, and one month before the sample collection. We defined prevalent cardiovascular disease at baseline by searching the NPR for the ICD10 codes I20-I25, I30-I52, and I60-I69. Diagnosis codes as main or secondary diagnoses based on ICD versions 8-10 were also collated from the NPR (1964 to baseline) to calculate a weighted Charlson comorbidity index.^36^ In addition to proteomics, 25 more conventional chemical lab analyses have been completed as previously described,^36^^,^^37^ including plasma alanine aminotransferase, plasma Cystatin C, plasma creatinine, and serum 25-hydroxyvitamin D.

### Selection of genetic instruments for MR analyses

We strengthened causal inference regarding the effect of plasma protein biomarkers on hip fracture and several risk factors of hip fracture using MR analysis. The genetic instruments for the proteins (Supplementary Table S2, sheet 1) were obtained from the UK Biobank Pharma Proteomics Project (UKB-PPP).^38^ This study discovered and replicated pQTLs of 3072 plasma proteins measured by the Olink proteomics assay in 35,571 and 18,181 UKB participants, respectively. To limit horizontal pleiotropy,^39^ we only used the index *cis*-pQTL single nucleotide polymorphisms (SNP) of each protein as the genetic instrument. Summary-level statistics of the genetic instruments were from three sources: 1). The sentinel *cis*-SNPs in the combined UK Biobank cohort (p < 1.7 × 10^−11^); 2). For proteins with the sentinel *cis*-SNPs being multiallelic, we used the *cis*-SNPs in the discovery and the replication cohorts (p < 1.7 × 10^−11^); 3). For the sentinel cis-SNPs being multiallelic in both the combined and discovery-replication cohorts in the UK Biobank, we used the *cis*-SNPs (within a 500 kb window of the protein-coding gene) with the lowest p-value (p < 5 × 10^−8^) and being biallelic in the combined cohort. Detailed definitions of the cohorts can be found elsewhere.^38^ The exact p-values of the selected cis-pQLTs as genetic instruments can be found in Supplementary Table S2, sheet 1. We provide the *F*-statistic for the SNP used as a genetic instrument for each plasma protein in Supplementary Table S2, sheet 1, calculated using the formula:

$$F-statistic={\left(\frac{beta}{se}\right)}^{2}$$

where beta and se are the beta coefficient and standard error of the SNP-exposure association, respectively.^40^ Data on SNPs associated with the outcome of femoral fracture (ICD10 code S72 and ICD8-9 codes 820) were obtained from a genome-wide association analysis involving 9489 register-identified cases and 402,692 non-cases of European ancestry of the FinnGen consortium (R10 release).^41^ The femoral fracture genetic data from FinnGen largely reflect a hip fracture since 90% of the patients with femoral fracture specifically had had a hip fracture (ICD10 codes S720-S722 or ICD 8-9 code 820). We lack GWAS data based on relevant and clinically confirmed fragility fracture composite endpoint information for fractures that occur at an advanced age. Body composition outcomes in MR analyses were femoral neck, lumbar spine, and total body BMD with genetic data obtained from the GEFOS database (www.gefos.org).^42^^,^^43^ From the UK Biobank study, we attained summary genetic data on estimated heel BMD,^44^ whole body fat mass (mrcieu gwas_id ukb-a-265), whole body fat-free (mrcieu gwas_id:ukb-a-266), leg fat mass (mrcieu gwas_id:ukb-b-7212 and mrcieu gwas_id:ukb-b-18096), hip circumference (mrcieu gwas_id:ukb-b-15590), waist-to-hip ratio adjusted for body mass index (mrcieu gwas_id:ebi-a-GCST90025996), as well as register-identified falls (ICD10-codes R29.6 and W00-W19 in FinnGen, R10 release, with n = 102,834; https://r.10finngen.fi) and grip strength (mrcieu gwas_id ukb-b-7478) as markers of frailty and muscle function. The three core instrumental variable assumptions for Mendelian randomisation analysis are relevance, independence, and exclusion restriction.

### Statistics

#### Protein identification

Sample size determination, randomisation and blinding were not used in this study since data were from cohort studies. In the discovery cohort, associations between each protein biomarker concentration and time to hip and any fracture were investigated using Cox regression to estimate hazard ratios (HRs) with 95% confidence intervals (CIs). We calculated the time at risk for hip fracture and any fracture for each participant from baseline (date of blood draw) until the date of the first outcome diagnosis, date of death, or end of follow-up on December 31, 2021, whichever came first. We excluded individuals with a history of cancer before baseline but retained individuals with a history of a previous fracture. For proteins associated with fractures in the discovery cohort, we repeated the Cox regression analyses using data from the replication cohort. Replicated fracture-related proteins were then analysed, using multivariable linear regression analysis, for their association with continuous outcomes in the discovery cohort: BMD and BMA at the total hip, lumbar spine, and whole body; serum CrossLaps and serum osteocalcin; total fat mass, and total lean mass. We used the Benjamini and Hochberg method^45^ and a false discovery rate of 5% to select proteins to take forward to replication analyses. We used a nominal p-value cut-off of 0.01 in the replication analyses to consider the protein-fracture associations as reproduced for the total cohort or in sex-stratified analyses. Applying nominal p-values in the replication phase is sufficiently conservative to reduce the risk of false-positive findings.^46^ We also conducted sensitivity analyses examining the association of all 274 proteins with hip fracture and all types of fracture in the replication cohort, overall and stratified by sex.

The primary analyses were adjusted for age (continuous) and sex. Secondary models were used for sensitivity analysis with additional adjustment for smoking status (current vs. no smoking), education level (three categories), body mass index (continuous), height (continuous), cardiovascular disease at baseline, baseline weighted Charlson comorbidity index (continuous), liver function estimated by baseline plasma alanine aminotransferase (continuous), estimated glomerular filtration rate by use of both plasma Cystatin C and creatinine (continuous).^36^^,^^37^ We also performed sensitivity analyses excluding women and men with prevalent CVD or any prevalent comorbidity included in the Charlson index.

All analyses were performed using Stata version 15.0 (Stata Corp., College Station, TX, USA) or R Statistical Software (v4.1.2; R Core Team 2021).

#### Network analysis of fracture-related proteins

Since many proteins co-vary and may share common background causes, a deeper understanding of these underlying patterns is needed. Firstly, we computed a correlation matrix heat map for all fracture-related proteins displaying age- and sex-adjusted Pearson correlation coefficients. In a figure, we then integrated correlations (absolute |r| > 0.3) between the proteins, statistical strengths of linear associations between proteins and BMD (shown as circles if p-values <0.05, diamonds if p-values ≥0.05), and hazard ratios of proteins with both hip and any fracture (bars). The sizes of the circles and bars are proportional to a reciprocal function of the p-values for the associations. Thirdly, we used a visualisation tool^47^ to provide further insights into exposure-protein-disease associations. This tool simultaneously integrates and displays proteins through principal components and their correlations with 51 fracture risk factors.^47^ Fourthly, we used the STRING (Search Tool for the Retrieval of Interacting Genes/Proteins) database (http://string-db.org) to analyse known and predicted protein-protein interactions. STRING derives interactions from genomic context predictions and co-expression analyses, results from high-throughput laboratory experiments, automated text mining of scientific articles, and previous knowledge accessed in databases.

#### Mendelian randomisation

For replicated fracture-related proteins, we used the Wald ratio method and the R package *TwoSampleMR*^48^ to estimate associations of genetically predicted protein concentrations with femoral fracture, body composition outcomes (BMD, fat mass, lean body mass, and hip circumference), falls, and grip strength. The effect size estimates of *cis*-pQTLs SNPs from the discovery-replication cohort of UKB-PPP (genetic instrument source No.2 as described above) were meta-analysed using the R package *Metafor*^49^ to generate MR instrument statistics via a linear fixed-effects model with inverse-variance weights. The MR estimate was computed by dividing the beta coefficient for the SNP-outcome association by the beta coefficient for the SNP-protein association, and the corresponding confidence intervals (CI) were estimated using the delta method.^50^ We used Steiger filtering^51^ to indicate whether the estimated effect was correctly orientated from protein levels to the outcomes and not due to reverse causation. In addition, to examine potential for horizontal pleiotropy, we used Ensembl (https://www.ensembl.org/index.html) to identify genes ±150 kbp of index SNPs that were significantly associated with at least one outcome in MR. We then used publicly available gene expression data in the GTEx portal (https://gtexportal.org/) to examine if these index SNPs were associated with gene expression of another protein-encoding gene in whole blood.

False discovery rates were applied for each outcome (femoral fracture [24 tests], falls [24 tests], and grip strength [22 tests]) or set of outcomes (i.e., BMD and whole body composition [158 tests] and lower limb fat distribution [74 tests]) separately.

#### Colocalisation analysis

We further implemented colocalisation analysis to understand whether the associations of the proteins of interest (FDR corrected p-value <0.1) with respective outcomes confirmed by MR were driven by distinct genetic variants in linkage disequilibrium (LD) using the R package *coloc*.^52^ For all SNPs within the predefined genomic region (500 kb within the protein-coding genes) of traits 1 and 2, the Bayesian-based method assesses the support for the following five exclusive hypotheses: 1). H_0_, no association with either trait; 2). H_1_, association with trait 1 not with trait 2; 3). H_2_, association with trait 2 but not with trait 1; 4). H_3_, association with both traits, with two distinct SNPs; 5). H_4_, an association with both traits by one shared SNP.^52^ This traditional method assumed that each trait has at most one causal signal per locus. To deal with multiple independent associations for the same trait, we used the Sum of Single Effects (SuSiE) framework^53^ adopted in the *coloc* package by integrating protein-outcome GWAS data with a correlation (LD) matrix of SNPs genotyped in individuals of European ancestry from the 1000 Genomes Project Phase 3. For both analyses, we set the prior probabilities of the SNPs being associated with only trait 1 (p1) or with only trait 2 (p2) as the default value in the package, 1e-4. For the prior probability of the SNPs being associated with both trait 1 and trait 2 (p12), we used a more conservative value, 5e-6, as suggested previously for improved robustness.^54^ The sensitivity of the colocalisation results to the variation in p12 was further examined by genetic locus visualisation.

### Ethics

The Swedish Ethical Review Authority granted ethical approval for this study, with reference numbers 2018/261, 2018/262, and 2018/263. All participants provided written informed consent.

### Availability of data and materials

Data cannot be shared publicly because of their sensitive nature and the GDPR legislation. However, data are available from the national research infrastructure SIMPLER for researchers who meet the criteria for access to confidential data. Details of obtaining data from the national research infrastructure SIMPLER can be obtained at the website www.simpler4health.se.

### Role of funders

The funding source had no role in the study design, data collection, data analyses, data interpretation, writing of the manuscript, or decision to submit it for publication.

## Results

Table 1 displays the baseline characteristics of the discovery (n = 5007) and replication (n = 7307) cohorts. The average age at baseline was 68 years in the discovery cohort of women and 73 years in the mixed-sex (35% women) replication cohort. Body mass index and energy intake distributions in women were similar in the cohorts. Energy intake and body size in men were substantially higher than in women. The discovery cohort had a higher educational level.

**Table 1:** Baseline characteristics of the participants in the discovery and replication cohort.

Variable	Level	Discovery cohort	Replication cohort by sex	
		n = 5007	Female n = 2572	Male n = 4735
Age at baseline, mean (SD)	years	67.6 (6.8)	74.4 (3.7)	74.3 (6.0)
Weight, mean (SD)	kg	69.6 (12.0)	69.9 (12.3)	82.3 (12.9)
Height, mean (SD)	cm	163.6 (6.1)	162.3 (6.1)	176.0 (6.6)
Body mass index, mean (SD)	kg/m^2^	26.0 (4.3)	26.6 (4.5)	26.6 (3.7)
Total fat mass, mean (SD)	g	26,867 (8670)	NA	NA
Total lean mass, mean (SD)	g	39,446 (4401)	NA	NA
BMD total hip, mean (SD)	g/cm^2^	0.92 (0.13)	NA	NA
Bone area total hip, mean (SD)	cm^2^	32.5 (2.2)	NA	NA
S-Beta CrossLaps, mean (SD)	ng/L	461 (192)	NA	NA
S-Osteocalcin, mean (SD)	ug/L	25.0 (8.9)	NA	NA
Energy intake, mean (SD)	kcal	1810 (542)	1863 (533)	2438 (704)
Cardiovascular disease,^a^ n (%)	0	4473 (89.3%)	1872 (80.4%)	3160 (66.7%)
	1	534 (10.7%)	455 (19.6%)	1575 (33.3%)
Charlson comorbidity index, n (%)	0	4265 (85.2%)	1835 (71.3%)	3005 (63.5%)
	1	470 (9.4%)	318 (12.4%)	809 (17.1%)
	2	272 (5.4%)	419 (16.3%)	921 (19.5%)
Smoking status, n (%)	Current	1090 (22.1%)	453 (19.8%)	923 (19.7%)
	Former	1332 (26.9%)	634 (27.7%)	1852 (39.5%)
	Never	2521 (51.0%)	1202 (52.5%)	1919 (40.9%)
Education level, n (%)	≤9 years	2696 (53.9%)	1486 (64.0%)	2761 (58.6%)
	10–12 years	443 (8.8%)	284 (12.2%)	937 (19.9%)
	>12 years	1867 (37.3%)	553 (23.8%)	1015 (21.5%)
Physical activity, n (%)	<1 h/week	887 (19.2%)	347 (16.0%)	899 (20.6%)
	1 h/week	1197 (26.0%)	565 (26.0%)	961 (22.0%)
	2–3 h/week	1571 (34.1%)	809 (37.2%)	1446 (33.1%)
	4–5 h/week	518 (11.2%)	246 (11.3%)	500 (11.5%)
	>5 h/week	436 (9.5%)	207 (9.5%)	559 (12.8%)

SD: standard deviation; NA: not available; BMD: bone mineral density.

a Cardiovascular diseases identified from ICD-10 codes I20-I25, I30-I52, and I60-I69, recorded in the Swedish National Patient Register.

**Fig. 2:**
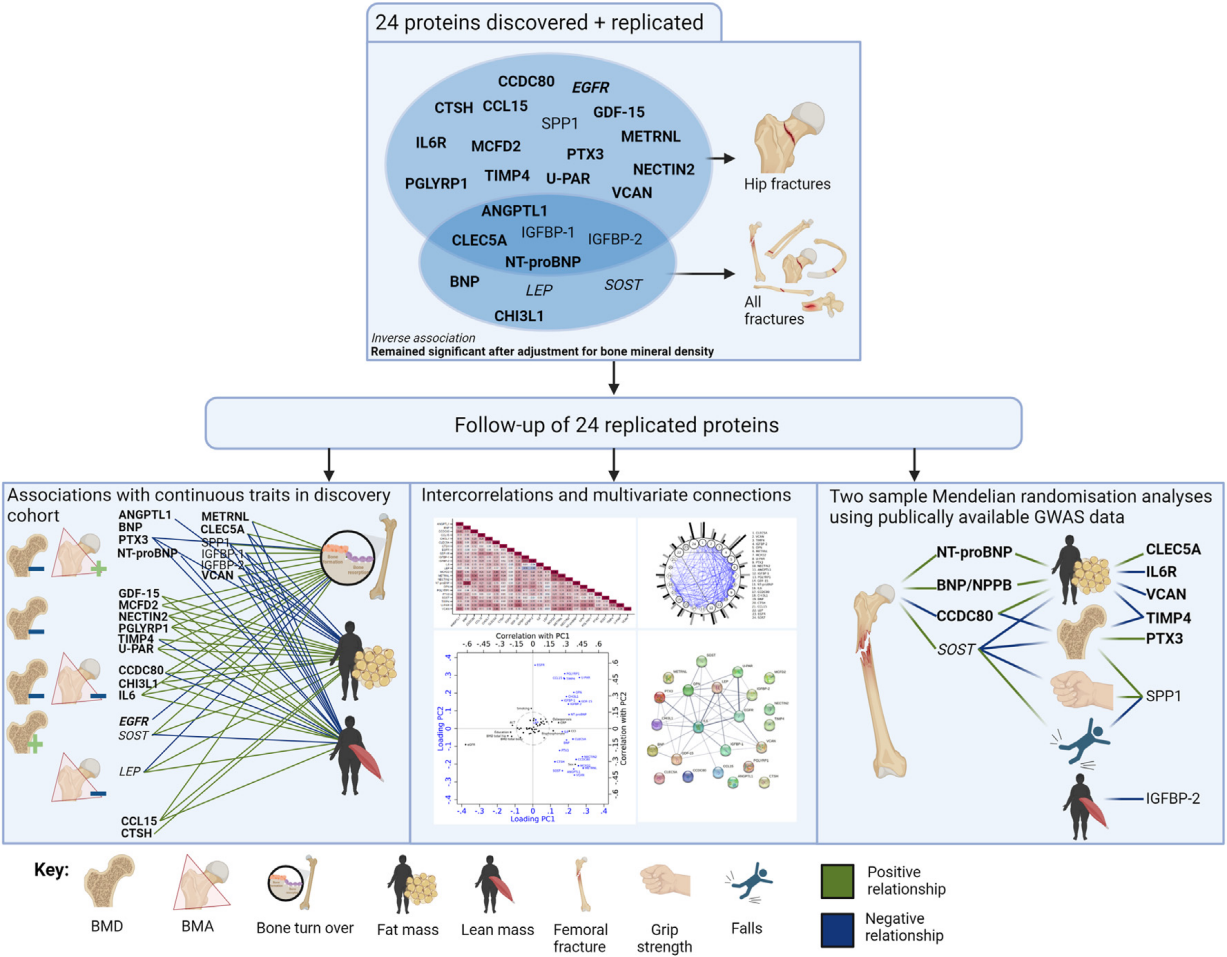
Overview of study results showing 24 discovered and replicated proteins associated with hip fracture and all fracture, as well as follow-up analyses. BMD: Bone Mineral Density; BMA: Bone Mineral Area. Created with BioRender.com.

The following results are divided into four sections: 1). cohort analyses of cardio-metabolic-related proteins with fracture outcomes; 2). cohort analyses of fracture-related proteins' associations with secondary outcomes; 3). intercorrelation analyses of fracture-related proteins; 4). MR and colocalisation analyses of fracture-related proteins. The overall results can be found in Fig. 2; details of each section's findings are presented below.

### Proteins and fracture rates

**Fig. 3:**
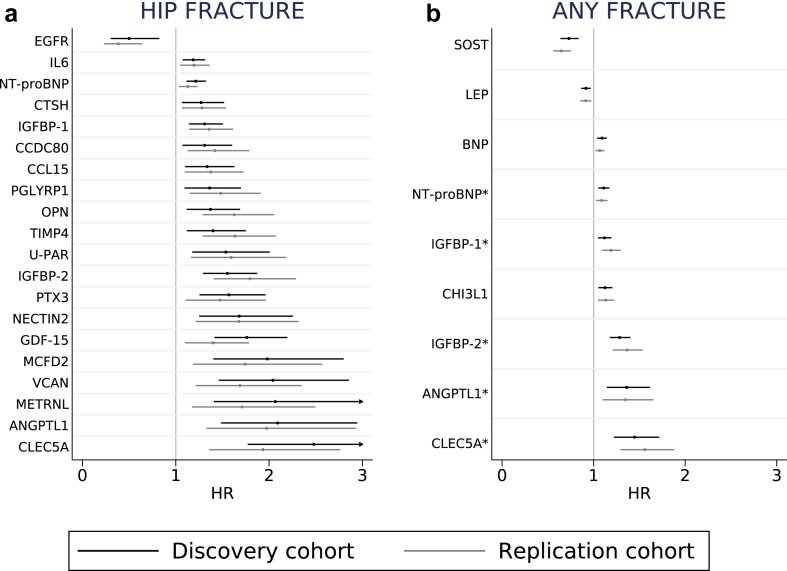
Age- and sex-adjusted hazard ratios (HRs) of hip fracture (panel a) and any fracture (panel b) per one higher plasma protein concentration unit in the discovery (black markers, Uppsala, only women) and replication (grey markers, Västerås, women and men) cohorts. The bars indicate 95% confidence intervals. An asterisk highlights proteins related to both hip and any fracture.

During 100,619 person-years of follow-up, 538 individuals had an incident hip fracture, and 2168 sustained any fracture (during 90,929 person-years). In the discovery cohort, we found 53 proteins (Supplementary Table S3) that passed the FDR correction significance level in both age-adjusted and multivariable models for hip or any fracture. We replicated 24 protein-fracture associations in the replication cohort: 20 for hip fracture, 9 for any fracture (Fig. 3, sorted by direction and strength of the hazard ratios), and five for both hip and any fracture. The HRs were similar in the discovery and the replication cohorts, with no statistical interaction with sex. The HRs for all 274 proteins with hip fracture and any fracture, overall and stratified by sex, in the discovery cohort and replication cohort are presented in Supplementary Table S4. The mean baseline concentrations of the 24 fracture-related proteins by cohort and sex are displayed in Supplementary Table S5.

Except for EGFR, all proteins associated with hip fracture conferred higher rates with increasing concentrations (Fig. 3a). For any fracture, both higher SOST and LEP concentrations conferred lower rates, while increasing concentrations of the other 7 proteins were related to higher rates (Fig. 3b). The results remained essentially unchanged after: additional adjustments for BMI and height (Supplementary Figure S1); adjustment for BMI, height and current smoking (Supplementary Figure S2); adjustment for baseline CVD (Supplementary Figure S3); exclusion of individuals with prevalent CVD (Supplementary Figure S4); adjustment for weighted Charlson comorbidity index (Supplementary Figure S5); exclusion of individuals with baseline comorbidity according to the Charlson comorbidity index (Supplementary Figure S6); adjustment for estimated glomerular filtration rate and plasma alanine aminotransferase (Supplementary Figure S7); and adjustment for educational level (Supplementary Figure S8).

Nineteen of the 24 proteins remained significantly associated with a risk of hip fracture or any fracture after adjustment for baseline total hip BMD (Supplementary Figure S9). Only IGFBP1, IGFBP2, and OPN of the 20 protein-hip fracture associations lost statistical significance. Estimates for the associations of SOST, LEP, IGFBP1, and IGFBP2 with any fracture were no longer statistically significant after BMD adjustment.

### Protein associations with BMD, bone area, and bone turnover markers in the discovery cohort

**Fig. 4:**
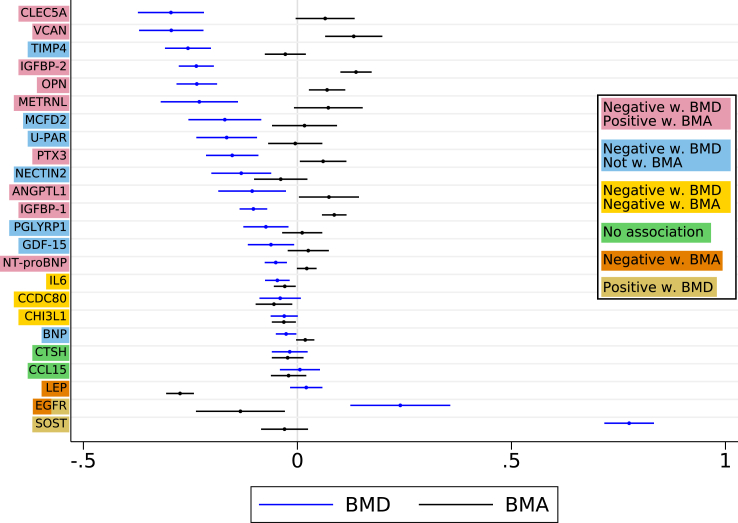
Age- and body mass index-adjusted parameter estimates of bone mineral density (BMD, blue markers) and bone mineral area (BMA, black marks) per one higher plasma protein concentration unit in women in the discovery cohort. The bars indicate 95% confidence intervals.

Both BMD and bone area directly affect bone strength and fracture risk, and we found a general trend that higher concentrations of most fracture-related proteins were associated with lower BMD and positive or neutral associations with BMA (Fig. 4). LEP and EGFR were, however, negatively associated with BMA, while both EGFR and SOST were the only proteins displaying a positive association with BMD.

**Fig. 5:**
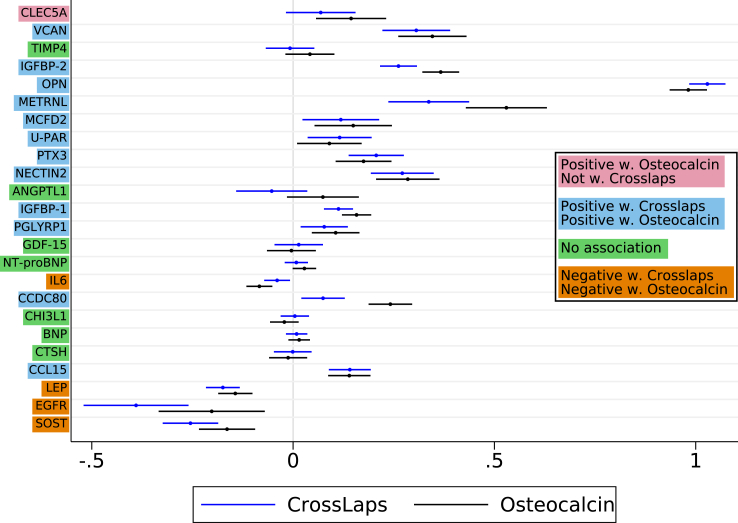
Age- and sex-adjusted parameter estimates of CrossLaps (blue markers) and osteocalcin (black marks) per one higher plasma protein concentration unit in women in the discovery cohort. The bars indicate 95% confidence intervals.

TIMP4, ANGPTL1, GDF-15, NT-proBNP, CHI3L1, BNP, and CTSH had no association with the circulating bone turnover markers CrossLaps and osteocalcin (Fig. 5). Higher LEP, EGFR, and SOST concentrations were related to lower bone turnover. In contrast, the other fracture-related proteins indicated an effect of higher bone turnover with increasing protein concentrations. OPN showed the strongest positive association with CrossLaps and osteocalcin among all fracture-related proteins.

### Proteins and body composition in the discovery cohort

**Fig. 6:**
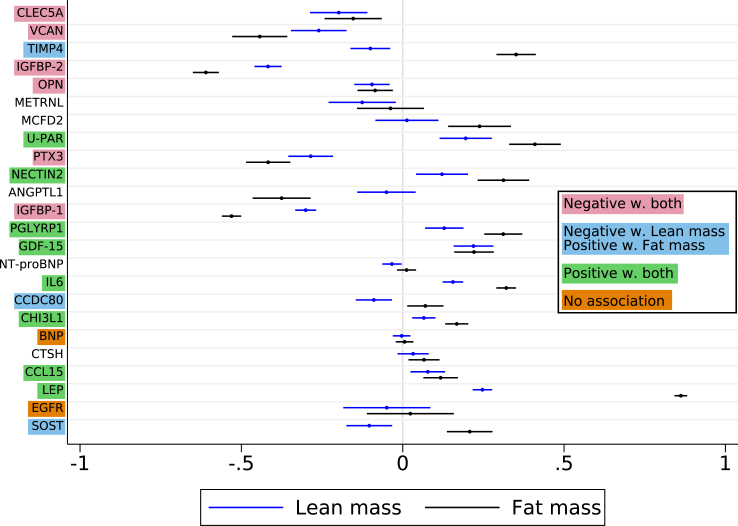
Age- and sex-adjusted parameter estimates of fat mass (black markers) and lean muscle mass (blue marks) per one higher plasma protein concentration unit in women in the discovery cohort. The bars indicate 95% confidence intervals.

Several proteins were related to fat and lean body mass indices (Fig. 6), generally in the same direction, with positive associations for 8 proteins and negative for 7 proteins. In contrast, higher concentrations of TIMP4, CCDC80, and SOST were all associated with higher fat but lower lean mass. EGFR and BNP were not associated with body composition, while NT-proBNP had a modest negative relationship with lean mass.

### Intercorrelations between fracture-related proteins and their multivariate connections

**Fig. 7:**
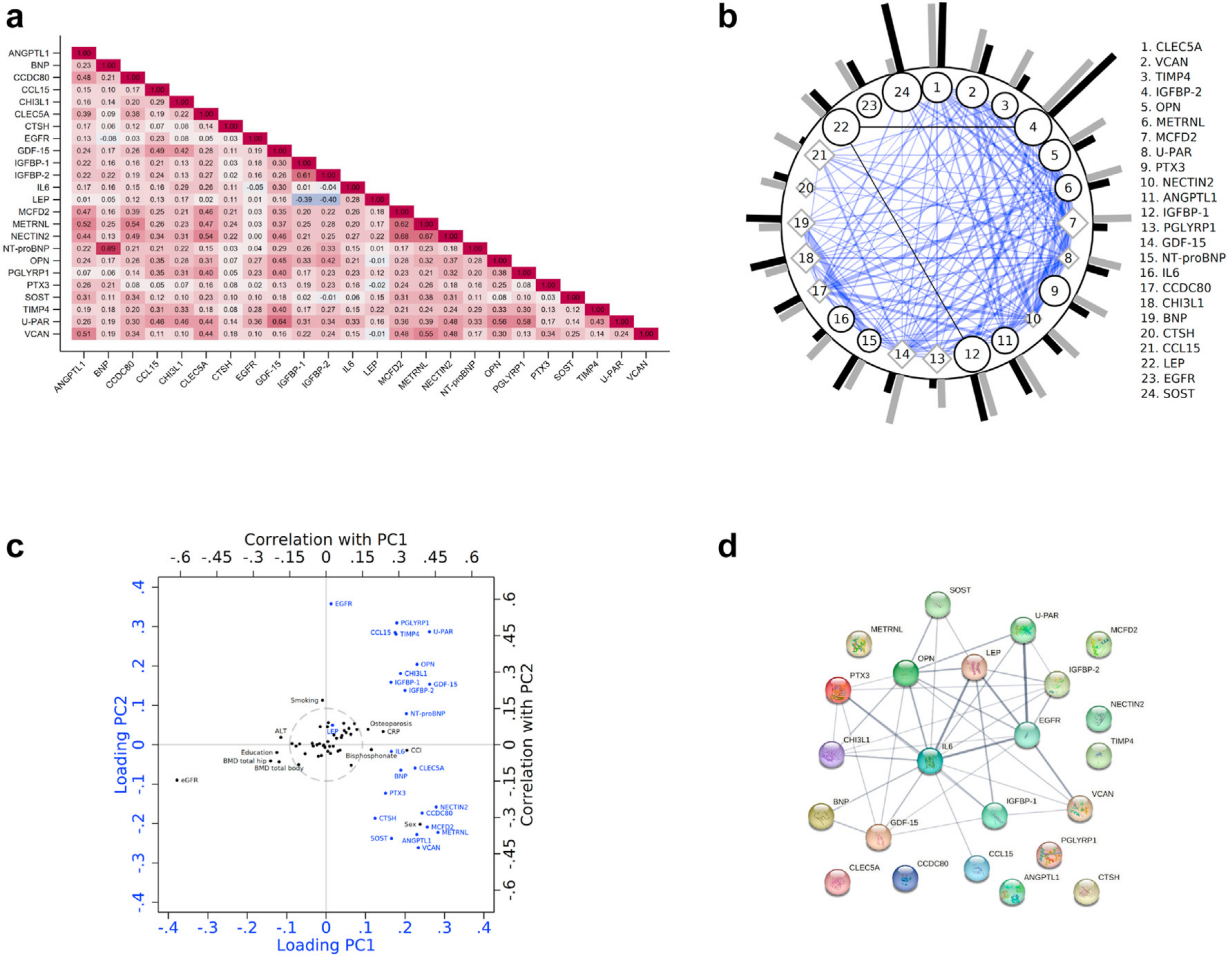
**(a).** A heat map displaying age- and sex-adjusted Pearson correlation coefficients between all 24 circulating fracture-related proteins. Cells in blue display positive correlations and red negative correlations. **(b).** The blue lines () represent positive correlations between proteins (labelled by a number from 1 to 24), while the black lines () represent negative correlations. The width of the line corresponds to the strength of the correlation. We only display correlations higher than 0.3 in absolute values and the correlations range between −0.37 and 0.90. Either black circles () or grey diamonds () surround the protein numbers. Black circles signify that the coefficient in an age-adjusted linear regression between BMD and protein has a p-value < 0.05. Grey diamonds mean that the corresponding coefficient has a p-value ≥ 0.05. The size of the shapes represents the size of the p-value, where a large shape corresponds to a low p-value. The area is proportional to (−logp)18. The p-values in these linear regressions range from 1.7 × 10^−143^ to 0.97. The grey bars () from the solid circle represent the p-value after a Cox regression analysis associating the protein with hip fracture, adjusted for age, site, and sex using pooled data. The length of the bar is proportional to −logp. The p-values in these Cox regression analyses range between 3.3 × 10^−11^ and 0.0045. The black bars () are similar to the grey bars but with any fracture as the outcome. These p-values range between 2.3 × 10^−14^ and 0.23 **(c).** Relation between fracture-related proteins (blue), expressed as two principal components (PCs), and 51 different variables (black) consisting of circulating biomarkers, various body composition measures, and lifestyle factors. The grey circle represents a cut-off of an absolute correlation of 0.15 to either of the two PCs. We only display names of variables connected to the two PCs with an absolute r-value greater than 0.15. The 51 tested variables were: serum total 25-hydroxyvitamin D, serum free 25-hydroxyvitamin D_3_, gynoid fat mass, android fat mass, gynoid lean mass, android lean mass, total fat mass index, total fat mass, total lean mass, body mass index, body weight, dual total hip BMD, BMD of whole body, BMD lumbar spine L1-L4, total energy intake, total protein intake, alcohol intake, calcium intake, estimated glomerular filtration rate (eGFR), plasma albumin, plasma LDL cholesterol, plasma HDL cholesterol, plasma triglycerides, plasma transferrin, serum CrossLaps, serum osteocalcin, serum sodium, serum potassium, plasma glucose, serum phosphate, plasma calcium, plasma CRP, plasma alanine aminotransferase (ALT), serum retinol, plasma insulin, plasma TSH, plasma parathyroid hormone (PTH), menopausal age, weighted Charlson comorbidity index (CCI), self-reported leisure time physical activity (four categories), education (four categories), season of blood draw, use of bisphosphonates, use of oestrogens, current smoking, diabetes, osteoporosis according to the DXA scan, living alone, male sex, and parity. **(d).** Construction of an interaction network between the fracture-related proteins using STRING tools. NT-proBNP is not available in STRING.

Fig. 7a displays the age- and sex-adjusted correlation coefficients among the 24 fracture-related proteins, with moderately strong positive correlations for NECTIN2 with METRNL and MCFD2 (r = 0.7), in addition to the natural prominent positive correlation between BNP and NT-proBNP (r = 0.9) and between IGFBP1 and IGFBP2 (r = 0.6). UPAR correlated with OPN, GDF15, and PGLYRP1 (r = 0.6). The strongest negative correlation was found between LEP and the two IGFBPs (r = −0.4).

Fig. 7b shows correlations (|r| > 0.3) among the proteins, p-values for age-adjusted linear regression between the proteins and BMD, and p-values for HRs of age- and sex-adjusted protein to time-to-fracture associations. The highest joint statistical strengths for both fractures and BMD as outcomes were found for CLEC5A, SOST, IGBP-1, and IGBP-2.

Fig. 7c displays the first two principal components of the fracture-related proteins and the correlation between these components with circulating biomarkers, body composition, and lifestyle factors, a total of 51 variables. Of those, only 11 factors (male sex, CCI, current smoking, bisphosphonate use, osteoporosis, BMD total hip and total body, circulating CRP and ALT, glomerular filtration rate, and educational level) were moderately correlated (|r| > 0.3) with either of the two principal components. Among the 40 factors only weakly (|r| < 0.3) related to the 2 principal components were serum total 25-hydroxyvitamin D, serum-free 25-hydroxyvitamin D3, blood lipids, and physical activity.

Fig. 7d was constructed using the web-based STRING tools to create a comprehensive network of the proteins based on prior scientific knowledge to reveal the potential interactions among the fracture-related proteins. The most prominent interactions were identified between OPN, LEP, IL-6, U-PAR, and EGFR. Ten proteins, among them CCDC80, were not at all related to any other fracture-related protein.

### MR analysis of proteins with femoral fracture, body composition, falls, and strength

**Fig. 8:**
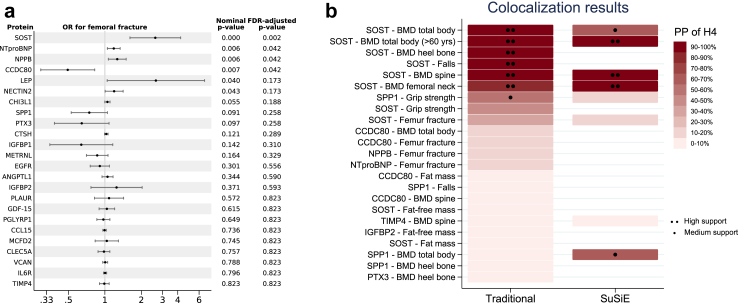
Mendelian randomisation femoral fracture results for 24 fracture-related proteins replicated in the observational part of our study (Panel a). Odds ratio values are on the x-axis presented on a log-scale, and nominal and FDR-corrected p-values are displayed. Panel b displays the results for the for the two colocalisation analyses. H4 denotes an association with both traits, by one shared SNP.

Four proteins remained statistically significant in the MR analysis after an FDR-corrected causal association analysis between the proteins and risk of femoral fracture (Fig. 8a and Supplementary Table S2, sheet 2). A genetically predicted one standard deviation (SD) increment in SOST conferred an odds ratio for femoral fracture of 2.61 (95% CI 1.62–4.23; p_nominal_< 0.001; p_fdr_ = 0.002). Higher predicted circulating SOST was also associated with lower bone mineral density (Supplementary Table S2, sheet 3), higher risk of falls (Supplementary Table S2, sheet 4), and lower grip strength (Supplementary Table S2, sheet 5). For the closely connected proteins NTproBNP and BNP, higher predicted blood levels conferred a higher risk of femoral fractures (Fig. 8a), as well as higher hip circumference and leg fat mass (Supplementary Table S2, sheet 6) but no associations with BMD, risk of falls, or grip strength (Supplementary Table S2, sheets 3–5). In addition, we found evidence of association (Fig. 8a) between increased CCDC80 (Coiled-coil domain-containing protein 80) and reduced hip fracture risk, with OR 0.49 (95% CI 0.30–0.82; p_nominal_< 0.007; p_fdr_ = 0.04). Genetically predicted higher CCDC80 levels were, however, negatively related to BMD and positively to total fat mass, hip circumference, and fat mass in the legs.

MR analyses further revealed an apparent negative FDR-corrected effect between genetically predicted circulating TIMP4 and BMD (Supplementary Table S2, sheet 3), while we observed positive associations for PTX3 and SPP1 with BMD. At the same time, higher genetically predicted levels of SPP1 were associated with a lower risk of falls and higher grip strength. According to the Steiger analyses, all MR associations were directed from the proteins to the outcomes, except for the bidirectional association between SOST and BMD (Supplementary Table S2, sheets 7–10).

The MR analyses further revealed negative associations between TIMP4 and leg fat mass and IGFBP2 with total fat-free mass (Supplementary Table S2, sheets 6 and 3, respectively). A positive association by CLEC5A on leg fat mass was indicated, and a negative association on waist-to-hip ratio by VCAN and IL6R (Supplementary Table S2, sheet 6). These analyses were aimed at exploration; thus, no further sensitivity analyses were conducted.

Examining genes ±150 kbp of index SNPs that were significantly associated with at least one outcome in MR (Supplementary Table S6) in the GTEx portal (https://gtexportal.org/), revealed that the SNP used as an instrument for BNP (rs198389) was associated with gene expression of *MTHFR* in whole blood, and the SNP used as an instrument for SPP1 (rs56254643) was associated with gene expression of *PKD2* in whole blood, both with a p-value smaller than the GTEx stated p-value threshold (Supplementary Table S7).

### Colocalisation of proteins with femoral fracture and body composition

We performed colocalisation analyses for all MR associations that had an FDR-adjusted p-value <0.1 (nominal p-values <0.01) (Fig. 8b and Supplementary Table S8). Out of 23 protein-outcome MR associations investigated, 7 associations supported colocalisation by the traditional coloc method. Among them, high support (PP.H4 > 0.8) was seen for SOST with total body BMD, total body BMD (>60 yr), heel BMD, falls, spine BMD, and femoral neck BMD. Medium support (0.5 < PP.H4 ≤ 0.8) was seen for SPP1 with grip strength (Supplementary Figure S10). Using SuSiE, additional evidence of supporting medium colocalisation of SPP1 and total body BMD was found.

## Discussion

This comprehensive study identified 24 firmly established cardio-metabolic proteins (Supplementary Table S9) associated with fracture risk in older women and men, even after considering prevalent cardiovascular disease at baseline. We found that most of these associations persisted even after adjustment for BMD, a prominent traditional risk factor for fragility fractures. Interestingly, more proteins were related to hip fractures than for any fractures. Hip fractures, compared with any fragility fracture, are more strongly associated with low BMD.^55^

The results of the extensive analyses on varying outcomes are discussed in depth below, organised into three main sections. Firstly, we discuss how established cardiovascular disease protein biomarkers were revealed to affect fracture risk. In particular, in line with the analysis of observational data, we found a strong indication of a direct causal relationship of NT-proBNP and BNP with increased risk of hip fracture using MR analysis. In contrast, for CCDC80 the results of the observational analysis and the MR were in opposite directions. Secondly, we discuss our observation of complex systemic effects of the bone-related proteins sclerostin (SOST), osteopontin (SPP1), and epidermal growth factor receptor (EGFR). Specifically, MR analyses for SOST and SPP1 conflicted with the results from the observational analyses. Discrepancies between observational and MR analyses suggest complex feedback loops that need to be better understood. Meanwhile, EGFR was the only protein inversely associated with hip fracture risk, but no causal relationship was suggested in the MR analysis. Thirdly, we discuss how inflammation-related proteins involved in cardiovascular disease are expected to impact fracture risk. Similar to SPP1, the results of the observational and MR analyses were contradictory for PTX3. Further, CLEC5A was one of the proteins with the strongest associations with hip fracture, but MR results did not suggest a causal effect on fracture risk. Overall, there were 13 fracture related proteins identified in the observational analyses for which we did not find evidence of a causal relationship based on MR analysis. Nonetheless, these could still be causally related to fracture-developing mechanisms indirectly.

During the last two decades, the age-adjusted incidence of both myocardial infarction and stroke in Sweden has decreased by 40%. The age-adjusted incidence of hip fractures during the same period has been reduced by 30%.^56^ Moreover, 85-year-olds born in the 1930s face less disability than cohorts born 20 or 30 years earlier, and the average expected life span continually increases.^57^ These downward trends may be explained by a generally improved lifestyle and health status over time.^56^, ^57^, ^58^ Our identified common proteomic keys for fragility fractures and cardio-metabolic diseases can be of interest as biomarkers of general health.

### Established cardiovascular disease protein biomarkers, now revealed to affect fracture risk

Our observation, both in the discovery-replication analysis and with MR analysis, that NT-proBNP and BNP act as two associated biomarkers for individuals at a heightened risk of future fragility fractures discloses a likely shared pathogenic pathway between fractures and cardiovascular disease. Our finding aligns with previous twin research,^2^ which highlighted various cardiovascular diseases, particularly heart failure, as early signs of increased fragility fracture risk. Our current analysis further strengthens this association,^2^ demonstrating that higher plasma concentrations of BNPs were consistently associated with fracture risk even after the exclusion of those with baseline co-morbidities such as CVD and heart failure. The biologically active BNP, with the highest concentrations found in the myocardium, acts through binding to the natriuretic peptide receptor A. Circulating levels of the related proteins BNP (32 amino acids) and NT-proBNP (76 amino acids) are used clinically to diagnose acute congestive heart failure, and blood concentrations are also typically increased in patients with asymptomatic or symptomatic left ventricular dysfunction.^59^ BNP has blood pressure-lowering effects, regulates water-salt balance, increases natriuresis, blocks cardiac hypertrophy and fibrosis, and inhibits the renin–angiotensin–aldosterone system (RAAS).^60^^,^^61^ BNP analogues have been tested as blood pressure-lowering drugs.^60^^,^^61^ Thus, higher BNP concentrations seen with cardiac dysfunction should be seen as a compensatory response to high ventricular filling pressures.^59^

Pre-clinical data also support these findings. Transgenic mice with elevated plasma BNP concentrations exhibit deformed skeletons by a mechanism of osteoclast recruitment affecting endochondral ossification, leading to increasing femur bone marrow width, albeit simultaneously with a normal BMD.^62^ We observed moderately strong associations between higher NT-proBNP with lower BMD and higher BMA. Still, our MR and colocalisation analyses did not reveal a statistically significant direct impact by BNP on bone or the other main measured fracture risk factors. No animal studies have evaluated the net effect on bone bending strength after an elevation of plasma BNP. NT-proBNP is also a predictor of loss in muscle mass,^62^ and we found an inverse observational association between this protein and lean muscle mass. Moreover, an exploratory MR analysis displayed a positive association between the two BNP proteins and higher hip circumference. A large pelvis and body size positively correlate with hip axis length and femoral neck angle,^63^ risk factors for hip fracture,^64^, ^65^, ^66^ even after adjustment for FRAX risk factors, BMD, and body height.^65^

In the first observational part of our study, increasing concentrations of the adipocytokine coiled-coil domain-containing 80 (CCDC80) were related to higher hip fracture risk, lower BMD and bone area, higher bone turnover, lower lean mass, and higher fat mass. In the MR analysis, we observed the complex pattern of lowered femoral fracture risk, reduced BMD, and a higher fat mass, with genetically predicted higher CCDC80 levels. There is no clear explanation for the fracture risk disagreement. Theoretically, horizontal pleiotropy is a concern, with the possibility of simultaneous up-or-down-regulation of proteins not examined in the study. Increasing concentrations of CCDC80 should theoretically lead to higher fracture risk. The protein impairs glucose tolerance, increases insulin resistance, increases the risk of cardiovascular disease,^67^^,^^68^ and accelerates atherosclerosis by decreasing lipoprotein lipase expression, increasing plasma triglyceride concentration. The knockout of CCDC80 decreases plaque lesion areas.^67^ CCDC80 plays dual roles in adipogenesis by mechanisms that involve at least in part down-regulation of Wnt/β-catenin signalling and induction of peroxisome proliferator-activated receptor γ (PPARγ),^68^ two critical regulators of bone homoeostasis.^69^ These changes in Wnt-signalling and PPARγ will both lead to bone loss.^69^ Human osteoblasts express CCDC80.^70^ Diabetes leads to a higher risk of fractures with mechanisms involving both a higher risk of falls and lowered bone size.^31^^,^^71^

TIMP-4 (Metalloproteinase inhibitor 4) influences platelet aggregation and is a negative regulator of atherosclerosis.^72^, ^73^, ^74^ TIMP-4 blocks the activities of several matrix metalloproteinases, which have a significant protective role in osteogenesis and bone regeneration.^75^ Both measured and genetically determined higher concentrations of TIMP-4 conferred lower BMD in our analyses while we found a higher risk of hip fracture in our discovery and replication cohorts but not in our MR analysis.

Higher levels of NECTIN-2 as a risk factor for hip fracture is also an important finding; the observation was evident in the cohort analysis and suggestive in the MR analysis (nominal p-value <0.05). Although there was an association of NECTIN-2 with lower BMD and higher bone turnover, the higher hip fracture rate remained after adjustment for baseline BMD. Interestingly, NECTIN-2 stimulates T-cell proliferation and cytokine production,^76^ and regulates angiogenic processes in vivo.^77^ There are genetic associations between the human NECTIN2 gene and both Alzheimer's and coronary heart disease.^78^

### Complex systemic effects of the bone-related proteins sclerostin (SOST), osteopontin (SPP1), and epidermal growth factor receptor (EGFR)

Osteocytes predominantly produce sclerostin/SOST, and the primary local function of SOST is to inhibit bone formation by binding to the membrane-bound cofactors LRP5 and LRP6. SOST thereby acts as a paracrine-acting antagonising regulator of Wnt/β-catenin signalling, inhibiting osteoblast differentiation and promoting osteoclast formation. This mechanism underlies the effect of romosozumab, a monoclonal antibody that binds to and inhibits sclerostin, to reduce fracture risk, increase bone formation, and decrease bone resorption.^79^ We found higher genetically determined concentrations of SOST to increase femoral fracture risk, reduce BMD and fat mass, lower grip strength, and increase risk of falls. The latter three findings support the view that muscle and bone can function as paracrine organs, secreting chemicals to modulate their own action.^80^ SOST may play a significant role in bone and muscle crosstalk by the Wnt/β-catenin signalling pathway, leading to muscle wasting and sarcopenia.^80^

Since SOST is expressed in arterial tissues and antagonises the Wnt pathway involved in cardiovascular remodelling, inhibition of SOST can promote vascular calcification and the risk of cardiovascular events; thus, SOST produced in arterial tissue may provide a natural defence mechanism against atherosclerosis.^81^, ^82^, ^83^, ^84^ There is growing evidence that the RANKL/RANK/OPG system contributes to the formation of vascular calcification, and SOST can stimulate the secretion of RANKL.^84^, ^85^, ^86^ Importantly, past research suggests high circulating SOST may benefit the cardiovascular system.^83^ Still, meta-analyses of RCTs provide contrasting views on whether using SOST-blocking antibodies impacts the risk of severe cardiovascular events.^87^^,^^88^ SOST genetic variants related to lower circulating SOST and higher BMD also confer a higher risk of myocardial infarction, coronary revascularization, and major adverse cardiovascular events and are associated with some cardiovascular risk factors such as type 2 diabetes mellitus, high blood pressure, and central adiposity.^87^ In contrast, ordinary observational studies found a positive correlation between high circulating SOST levels and cardiovascular mortality.^89^

Our observational discovery-replication results displayed increasing circulating SOST concentrations related to a lower risk of fracture and higher BMD. In contrast, our MR analysis showed higher hip fracture risk and lower BMD with higher SOST expression. Such surprising and discordant results have been previously found between other observational studies and MR results.^90^, ^91^, ^92^ It can be explained by the fact that bidirectional causal effects and feed-back loops may exist, as elegantly demonstrated by Zheng et al.^90^ and now confirmed by us, i.e., positive causal effect of BMD on circulating SOST levels, and simultaneously a negative causal effect of SOST on BMD.^90^ Indeed, our Steiger test analysis indicated bidirectional effects of SOST and BMD.

In addition to the paracrine role of SOST within the bone, SOST has systemic metabolic effects leading to the accumulation of white adipose tissue, impaired insulin sensitivity, and impact on fatty acid metabolism.^87^^,^^93^ We found a positive association with fat mass index, a negative association with lean mass index, and positive associations with other fracture-connected cardio-metabolic and inflammation-related proteins such as NECTIN-2, ANGPTL1 (Angiopoietin-related protein 1), VCAN (Versican), MCFD2 (Multiple coagulation factor deficiency protein 2), METRNL (Meteorin-like), and CCDC80. Furthermore, our STRING analysis revealed biological connections of SOST with OPN, LEP, and IL6, supporting a link with bone, fat, and inflammation. In our MR analysis, VCAN and IL6R were negatively associated with fat content.

Epidermal growth factor receptor (EGFR) was the only protein in the discovery-replication analysis related to lower hip fracture rates with increased circulating concentrations. EGFR plays an anabolic role in bone metabolism in vivo, and a reduction in EGFR activity in osteoblasts leads to a remarkable decrease in trabecular bone mass, number, and thickness.^94^ Moreover, mice with a constitutively active EGFR allele display increased trabecular and cortical bone mass. Administering an EGFR inhibitor to wild-type mice caused a significant reduction in trabecular bone mass.^94^ Activation of EGFR has been implicated in blood pressure regulation, endothelial dysfunction, neointimal hyperplasia, atherogenesis, and cardiac remodelling.^95^^,^^96^ Furthermore, increased circulating EGFR may mediate accelerated vascular disease development associated with chronic inflammation.^95^^,^^96^ However, some EGFR inhibitor treatments may also lead to heart failure and atrial fibrillation.^97^ Deficient signalling of the EGFR is associated with Alzheimer's disease. In contrast, overexpression is associated with tumour development across a wide range of cancers, an effect that EGFR-specific monoclonal antibody inhibitors can prevent.^98^ Our MR results did not indicate a direct effect of EGFR on fracture risk.

SPP1 or osteopontin (OPN) has an essential role in neuron-mediated and endocrine-regulated bone mass maintenance but is also involved in biological activities such as proliferation, migration, and adhesion of osteoclasts to the surface of the bone. Osteoblasts, osteocytes, and osteoclasts express SPP1, and its activity is highly complex. We showed that higher SPP1 concentrations were associated with elevated hip fracture rates and lower BMD, bone turnover, and fat and lean mass. At the same time, our MR results showed a contrasting view that higher SPP1 levels lead to higher BMD, grip strength, and lower risk of falls—an indication of a complex feedback loop mechanism that needs to be better understood. On the other hand, with our colocalisation SuSie results, we found only modest support for a direct causal association between SPP1 and BMD. In contrast to SOST, our Steiger analysis did not reveal an apparent bidirectional causal pathway between SPP1 and BMD. Previous clinical studies have also shown that SPP1 is involved in bone strength and remodelling, suggesting that serum SPP1 positively correlates with lower BMD and that high circulating SPP1 levels could be targeted as a biomarker for early diagnosis of postmenopausal osteoporosis.^99^ A link to cardiovascular disease is apparent: SPP1 is involved in atherosclerotic plaque formation, ischaemic heart disease progression, and insulin resistance.^100^^,^^101^ Experimental studies have also indicated that SPP1 is essential in vascular remodelling, and restenosis.^100^, ^101^, ^102^ Atherosclerosis-modifying therapies with statins or angiotensin II receptor antagonists (ARBs) simultaneously reduce cardiovascular disease risk and circulating SPP1 levels.^103^ Usage of statins and ARBs also, in turn, lowers the risk of fragility fractures.^104^^,^^105^

### Inflammation-related proteins involved in cardiovascular disease are expected to impact fracture risk

The acute phase protein PTX3 showed similar associations as those for SPP1, with higher hip fracture risk, lower BMD, higher bone turnover, and lower fat and lean mass. Our MR results indicated increased BMD with higher PTX3 expression, a pattern also seen for SPP1, but our colocalisation analysis indicated no direct impact of PTX3 on bone. Recently, PTX3 has emerged as a mediator of bone homoeostasis in rodents and human cell research.^106^^,^^107^ Human osteoblasts express the PTX3 protein, whose circulating concentrations positively correlate with bone density in vivo and osteoblast proliferation and maturation in vitro, thus indicating a role in bone formation. On the other hand, some evidence suggests osteoclastogenesis-promoting effects of PTX3, leading to bone resorption, and an inverse association between plasma PTX3 and BMD has previously been shown.^106^^,^^107^ PTX3 is a member of the pentraxin superfamily and acts as an acute phase response protein involved in inflammation response, and extracellular matrix remodelling, but PTX3 also has a beneficial atheroprotective impact in addition to harmful proinflammatory effects.^108^ Interestingly, vasculitis in autoimmune disease is influencing PTX3 levels,^109^ and PTX3 is involved in vascular inflammation and endothelial dysfunction. Patients with vasculitis have a higher risk of osteoporosis and fragility fractures.^110^ Plasma levels rise rapidly in acute myocardial infarction, heart failure, and cardiac arrest, reflecting the extent of tissue damage.^111^^,^^112^ PTX3 is also prognostic, reflecting the risk of CVD and all-cause mortality, independent of serum CRP levels or cardiovascular risk factors.^108^ Also, individuals with subclinical atherosclerotic lesions have higher serum PTX3 levels than those without atherosclerotic lesions.^108^

Finally, CLEC5A was one of the proteins with the strongest associations with hip fracture, any fracture, and BMD. However, MR results did not suggest a direct causal effect on fracture risk and BMD but a positive impact on fat mass. CLEC5A had a positive correlation (r = 0.6) with NECTIN-2, is strongly expressed in the bone marrow and trabecular bone, and its activation can lead to an inflammatory cascade, which can be reduced in vitro by a human CLEC5A monoclonal antibody.^113^ CLEC5A is strongly associated with activation of the inflammasome and pyroptotic cell death, which play vital roles in myocardial infarction. CLEC5A knockdown protects against cardiac dysfunction after myocardial infarction.^113^, ^114^, ^115^, ^116^

### There has been only one previous proteomic study of BMD and one hip fracture study

Only one cohort study, including 1874 men, has used large-scale proteomics and linkage to bone mineral density (BMD).^117^ Twenty annotated proteins were identified as related to BMD loss; five were additionally associated with self-reported hip fractures. No replication cohort was used, fracture-related proteins independent of BMD were not targeted, and the concentration of proteins was estimated from peptide compositions and not directly measured. Technical and analytical reasons necessitated that the 14 most abundant circulating proteins were not measured.^117^ We could not confirm the results of any of the five hip fracture-related proteins identified in this study^117^: CD14, CHL1, C7, PZP, and FCGBP. However, according to STRING, the first four are functionally related to several fracture-related proteins in our study (Supplementary Figure S11).

There is one carefully conducted recent proteomics and hip fracture study.^118^ The previous study used the SomaScan platform with 4979 aptamers/proteins, while we used the Olink platform and focused on 270 cardio-metabolic proteins. Although both the Olink and SomaScan platforms are affinity-based, they differ in nature. Olink is based on two antibodies for each protein, and the SomaScan is based on aptamers. When it comes to the correlation of circulating protein concentrations with variations in DNA coding regions, there is some supporting evidence for better assay performance with the Olink platform with, on average, stronger cis-SNP genomic associations.^119^^,^^120^ Moreover, there are modestly strong correlations between the platforms.^119^^,^^121^ None of the 23 hip fracture-related proteins identified in the study by Austin et al.^118^ were associated with fracture in the MR analysis. Still, two were related to bone density measures. The hip fractures in Austin et al.'s study were self-reported in one of the included cohorts and local register-identified in the other. Our analysis resulted in a higher proportion of the proteins related to fractures, of which four were associated with hip fracture using MR analysis. Nonetheless, three proteins (IGFBP-2, EGFR, and GDF15) with the strongest association with hip fracture in the Austin et al. study^118^ were also directionally equally associated with fracture in our study.

### Strengths and limitations

Our strengths include the study size and careful characterisation of study participants in both cohorts. Each cohort emanated from one of two adjacent counties but with socioeconomically different source populations, as indicated by a higher education level in the discovery cohort. A replication cohort is a significant strength of the study design, and the results were similar in both cohorts. Furthermore, proteins were measured in a multiplex proteomic assay using a PEA technique, which is both specific and sensitive. The selected three OLINK panels were highly suitable for providing insight into the connections between cardiovascular disease and fracture risk. We used complementary analysis to triangulate shared fracture and cardiovascular disease proteomic pathways (Supplementary Table S9), an approach to reveal potential therapeutic targets. Complete follow-up was enabled by national patient registers and individual personal registration numbers provided to all Swedish citizens.

One limitation is that only women were included in the discovery analysis. Different or additional proteins, or both, may have been associated with fractures had both sexes been included in the discovery cohort. i.e., we may have overlooked proteins that have a particular impact on fracture risk in men. In addition, the discovery cohort was slightly younger than the replication cohort, which may have similarly impacted the replication as well as the overall conclusions. Still, the replication analysis was done in both women and men without statistically significant protein-sex interaction, which suggests that the proteins that did replicate are likely generalisable to both women and men and across age groups. The cohorts included only participants of European ancestry, which might restrict the generalisability of our results to other populations. The MR direction of effect observed from quantifying circulating concentrations may not necessarily reflect the tissue activity for proteins with membrane-bound and unbound states. A weak instrument bias and the use of the UK Biobank for some MR outcomes (given that the same cohort was used to develop most genetic instruments) may be a concern.^122^ Still, our high F statistic values for these SNPs show that this will not likely be a problem.^123^^,^^124^ We used the same *cis*-SNP for both BNP and NT-proBNP, which could be seen as a violation of the assumptions underlying MR. Still, the approach is motivated by the fact that NT-proBNP is a prohormone of BNP, and our approach will only provide a conservatively biased MR result since we used the FDR correction. A distinct hip fracture phenotype for the MR analysis would have been preferable. Nonetheless, since 90% of the femur fracture outcomes in FinnGen are hip fractures, this misclassification is not a significant concern. A larger number of femoral fractures in the MR analysis could have provided more fracture-related proteins. In addition to our four fracture-related proteins identified in the MR analysis, an additional 7 proteins had nominal p-values <0.15. Additional results using the GTEx portal indicate that horizontal pleiotropy cannot be excluded for some proteins and suggest that future experimental studies are required to confirm the true causal relationship in these pathways. The cis-SNP used for BNP is, according to the GTEx results, associated with expression of *MTHFR* which encodes the protein methylenetetrahydrofolate reductase. Methylenetetrahydrofolate reductase plays an essential role in the removal of circulating homocysteine and has, in the last two decades, been implicated in the regulation of BMD; thus, it may serve as a potential risk factor for the development of fracture. Nonetheless, recent meta-analyses of observational studies and randomised controlled trials have not shown a clear benefit on bone-related outcomes or atherosclerosis development from lowering serum homocysteine concentrations.^125^, ^126^, ^127^, ^128^, ^129^, ^130^, ^131^ Although we used Steiger filtering to assess potential reverse causation, we cannot entirely rule out the possibility that the outcomes (e.g., hip fracture) affected the protein levels rather than vice versa. We were unable to conduct reverse MR analysis due to weak instruments for hip fracture, with few SNPs explaining a small proportion of the variance.^132^

Additional research is required to corroborate our results further. Moreover, further validation of the identified proteins as targets for pharmacological interventions is necessary, for example, through experimental studies using animal model systems or, for proteins that are already targets of existing medication, pharmacoepidemiological studies. Based on the consistency of associations in the observational and MR analyses, further investigation of NT-proBNP and BNP may be particularly interesting for future studies. At the same time, the importance of other proteins should not be discounted despite the lack of consistency between observational and MR results.

### Conclusion

In conclusion, we identified and replicated associations for hip or any fracture rates for 24 circulating proteins involved in cardio-metabolic pathways. All associations remained after adjustment for BMI and baseline comorbidities, and most remained after adjustment for BMD. Evidence supporting a direct causal impact on hip fracture risk or fracture risk factors was found for 11 proteins. These findings provide a common mechanistic bridge between CVD and fractures.

## Contributors

KM, JH, and LB contributed substantially to the study conception and design.

KM, AW, and LB contributed substantially to the acquisition of data.

KM, RZ, JH, BS, SCL, and SY contributed substantially to data analysis and accessed and verified the underlying data.

All authors contributed substantially to the interpretation of the data.

KM was responsible for the first draft of the manuscript.

All authors were involved in drafting the article or revising it critically for important intellectual content and agreed to be accountable for all aspects of the work ensuring that questions related to the accuracy and integrity of the work were appropriately resolved. All authors read and approved the final version of the manuscript.

## Data sharing statement

Data cannot be shared publicly because of their sensitive nature and the GDPR legislation. However, data are available from the national research infrastructure SIMPLER for researchers who meet the criteria for access to confidential data. Details of obtaining data from the national research infrastructure SIMPLER can be obtained at the website www.simpler4health.se.

## Declaration of interests

The authors have no relationships, activities or interests related to the content of the manuscript to declare.
