# Supplementary material for: Cardio-metabolic-related plasma proteins reveal biological links between cardiovascular diseases and fragility fractures: a cohort and Mendelian randomisation investigation
Source: eBioMedicine. 2025 Feb 6;113:105580. doi: 10.1016/j.ebiom.2025.105580 (PMC11848109; doi:10.1016/j.ebiom.2025.105580)
Supplement: Supplementary Table S5 [file mmc5.docx]

**Supplementary Table S5.** Baseline mean (SD) plasma protein concentrations of the discovery and replication cohort participants. The values are expressed in arbitrary units.

| **Plasma Protein** |  | **Discovery cohort**  **Women** | **Replication cohort by sex**  **Women Men** | |
| --- | --- | --- | --- | --- |
|  |  | n=5007 | n=2572 | n=4735 |
| SOST (Q9BQB4), mean (SD) |  | 4.80 (0.39) | 5.02 (0.45) | 5.29 (0.47) |
| EGFR (P00533), mean (SD) |  | 2.23 (0.21) | 2.21 (0.27) | 2.19 (0.25) |
| LEP (P41159), mean (SD) |  | 6.67 (0.90) | 7.07 (0.95) | 6.12 (1.08) |
| CCL15 (Q16663), mean (SD) |  | 6.40 (0.53) | 6.53 (0.58) | 6.53 (0.57) |
| CTSH (P09668), mean (SD) |  | 1.89 (0.58) | 2.30 (0.67) | 2.43 (0.73) |
| BNP (P16860), mean (SD) |  | 1.53 (1.09) | 2.30 (1.31) | 2.33 (1.54) |
| CHI3L1 (P36222), mean (SD) |  | 4.89 (0.81) | 5.14 (0.86) | 5.21 (0.93) |
| CCDC80 (Q76M96), mean (SD) |  | 5.75 (0.56) | 6.31 (0.59) | 6.17 (0.65) |
| IL6 (P05231), mean (SD) |  | 3.94 (0.91) | 4.35 (0.95) | 4.42 (0.90) |
| NT-proB (NA), mean (SD) |  | 2.67 (1.01) | 2.99 (1.07) | 2.96 (1.32) |
| GDF-15 (Q99988), mean (SD) |  | 3.64 (0.50) | 4.00 (0.56) | 4.15 (0.64) |
| PGLYRP1 (O75594), mean (SD) |  | 6.71 (0.48) | 6.76 (0.51) | 6.82 (0.52) |
| IGFBP-1 (P08833), mean (SD) |  | 4.66 (0.85) | 4.76 (0.85) | 4.68 (0.92) |
| ANGPTL1 (O95841), mean (SD) |  | 3.47 (0.32) | 3.77 (0.35) | 3.71 (0.35) |
| NECTIN2 (Q92692), mean (SD) |  | 6.38 (0.36) | 6.79 (0.41) | 6.86 (0.44) |
| PTX3 (P26022), mean (SD) |  | 1.34 (0.41) | 1.55 (0.48) | 1.62 (0.45) |
| U-PAR (Q03405), mean (SD) |  | 3.46 (0.36) | 3.62 (0.41) | 3.62 (0.44) |
| MCFD2 (Q8NI22), mean (SD) |  | 2.39 (0.29) | 2.71 (0.34) | 2.74 (0.36) |
| METRNL (Q641Q3), mean (SD) |  | 3.55 (0.29) | 4.00 (0.34) | 3.99 (0.39) |
| OPN (P10451), mean (SD) |  | 6.26 (0.52) | 6.48 (0.53) | 6.53 (0.54) |
| IGFBP-2 (P18065), mean (SD) |  | 7.73 (0.66) | 8.01 (0.62) | 8.03 (0.63) |
| TIMP4 (Q99727), mean (SD) |  | 3.17 (0.47) | 3.33 (0.50) | 3.20 (0.49) |
| VCAN (P13611), mean (SD) |  | 3.59 (0.33) | 4.01 (0.40) | 4.02 (0.42) |
| CLEC5A (Q9NY25), mean (SD) |  | 4.08 (0.33) | 4.28 (0.36) | 4.18 (0.38) |
|  |  |  |  |  |
|  |  |  |  |  |
