# Supplementary material for: Cardio-metabolic-related plasma proteins reveal biological links between cardiovascular diseases and fragility fractures: a cohort and Mendelian randomisation investigation
Source: eBioMedicine. 2025 Feb 6;113:105580. doi: 10.1016/j.ebiom.2025.105580 (PMC11848109; doi:10.1016/j.ebiom.2025.105580)
Supplement: Supplementary Table S9 [file mmc9.docx]

**Supplementary Table S9.** Evidence for a cardiovascular or metabolism connection for the 24 proteins from Olink’s panel CVD2, CVD3, and Metabolism identified associated with fragility fractures. Druggable protein targets are classified according to Finan et al. (1) in three classification categories:

**Tier 1.**

This tier incorporated the targets of approved drugs and drugs in clinical development.

**Tier 2.**

This tier incorporated proteins closely related to drug targets or with associated drug-like compounds.

**Tier 3.**

This tier incorporated extracellular proteins and members of key drug target families

| **Long protein name (short protein name)** | **Aliases** | **References** | **Drug development stage (1)** | **Cardiometabolic effects** |
| --- | --- | --- | --- | --- |
| **Brain natriuretic peptide** (BNP) | ANFB, NPPB, natriuretic peptide B, BNP, Iso-ANP | (2, 3) | Tier 3 | Blood pressure lowering, increases in both diuresis and natriuresis independently of changes in blood pressure, counteracting renin–angiotensin–aldosterone system (RAAS). Reduced serum concentrations with carvedilol, a non-selective beta-blocker with α-adrenoceptor antagonist properties. |
| **Angiopoietin-related protein 1** (ANGPTL1) | ANGPTL1, ANG3, ANGPT3, ARP1, AngY, UNQ162, dJ595C2.2, angiopoietin like 1 | (4-6) | Tier 1 | Antiangiogenic, positively correlated to the expression of other angiopoietin-related proteins that regulate lipid, glucose, and energy metabolism independently of angiogenic effects. |
| **Pro-cathepsin H** (CTSH) | CTSH, ACC-4, ACC-5, CPSB, minichain, ACC4, ACC5, cathepsin H, CATH | (7, 8) | Tier 2 | Pro-cathepsin H is active in cholesterol metabolism. Cardiovascular disease patients show increased cathepsin levels in the heart, aorta, and plasma. |
| **Coiled-coil domain-containing protein 80** (CCDC80) | CCDC80, CCD80, DRO1, SSG1, URB, okuribin, coiled-coil domain containing 80, CL2, LINC01279 | (9, 10) | Tier 3 | Adipocytokine affects glucose tolerance, insulin resistance, and cardiovascular diseases. Accelerates atherosclerosis development through decreasing lipoprotein lipase expression, increasing plasma triglyceride concentration. |
| **Chitinase-3-like protein 1** (CHI3L1) | CHI3L1, CH3L1, chitinase 3-like 1 (cartilage glycoprotein-39), ASRT7, CGP-39, GP-39, GP39, HC-gp39, HCGP-3P, YKL-40, YKL40, YYL-40, hCGP-39, chitinase 3 like 1, YK-40 | (11, 12) | Tier 3 | It has been strongly associated with asthma, arthritis, sepsis, diabetes, liver fibrosis, and Adipocytokine affects glucose tolerance, insulin resistance, and cardiovascular diseases. Accelerates atherosclerosis development through decreasing lipoprotein lipase expression, increasing plasma triglyceride concentration. |
| **C-type lectin domain family 5 member A** (CLEC5A) | CLEC5A, CLC5A, CLECSF5, MDL-1, MDL1, C-type lectin domain family 5 member A, C-type lectin domain containing 5A | (13-16) | No tier | CLEC5A is strongly associated with activation of the NOD-like receptor protein 3 (NLRP3) inflammasome and pyroptotic cell death, which play vital roles in myocardial infarction. CLEC5A knockdown protects against cardiac dysfunction after myocardial infarction. |
| **Versican core protein** (VCAN) | VCAN, CSPG2, ERVR, GHAP, PG-M, WGN, WGN1, versican | (17-19) | Tier 3 | Versican is increased after vascular injury and accumulates in advanced atherosclerotic plaques. Versican is also emerging as a potential target in controlling inflammation in several diseases, including cardiovascular diseases. Contributes to lipoprotein retention in atherosclerosis. One of 4 key proteins in a plaque protein signature that predicts future cardiovascular mortality. |
| **Epidermal growth factor receptor** (EGFR) | EGFR, ERBB, ERBB1, HER1, NISBD2, PIG61, mENA, epidermal growth factor receptor, Genes, erbB-1, ERRP | (20-22) | Tier 1 | - A transmembrane protein and its gene is an [oncogene](https://en.wikipedia.org/wiki/Oncogene), which has led to the development of anticancer therapeutics, EGFR inhibitors, or tyrosine kinase inhibitors. Activation of EGFR has been implicated in blood pressure regulation, endothelial dysfunction, neointimal hyperplasia, atherogenesis, and cardiac remodeling. Furthermore, increased circulating EGFR may mediate accelerated vascular disease development associated with chronic inflammation. However, some EGFR inhibitor treatments may also lead to heart failure and atrial fibrillation. |
| **Growth/differentiation factor 15** (GDF-15) | GDF15, GDF-15, MIC-1, MIC1, NAG-1, PDF, PLAB, PTGFB, growth differentiation factor 15, TGF-PL | (23, 24) | Tier 3 | The effects of GDF-15 are pleiotropic and include actions on metabolism, cell survival, immune response, and inflammation. GDF-15 also plays different roles in the pathophysiology of cardiovascular disease, autoimmunity, and diabetes. |
| **Insulin-like growth factor-binding protein 2** (IGFBP-2) | IGFBP2, IBP2, IGF-BP53, insulin-like growth factor binding protein 2 | (25-28) | Tier 2 | Low serum IGFBP2 increases the risk of obesity, type 2 diabetes, metabolic syndrome, and low insulin sensitivity. The protein is a therapeutic target for obesity-related insulin resistance. |
| **Multiple coagulation factor deficiency protein 2** (MCFD2) | MCFD2, CFD2, F5F8D, F5F8D2, LMAN1IP, SDNSF, multiple coagulation factor deficiency 2, multiple coagulation factor deficiency 2, ER cargo receptor complex subunit | (29-31) | No Tier | Mutations in this gene cause combined deficiency of FV and FVIII, a rare autosomal recessive bleeding disorder.  MCFD2 forms a Ca^2+^-dependent complex with LMAN1. This early secretory pathway has been suggested as a potentially attractive therapeutic target for anticoagulation. |
| **Nectin-2** (NECTIN2) | NECTIN2, NECT2, NECTIN-2, CD112, HVEB, PRR2, PVRR2, PVRL2, nectin cell adhesion molecule 2 | (16, 32-35) | No Tier | NECTIN‑2 stimulates T-cell proliferation and cytokine production and regulates angiogenic processes in vivo. NECTIN-2 is also a potential target for the clinical prevention of carotid atherosclerosis in the future. Deficiency in mice leads to cardiac fibrosis and dysfunction under chronic pressure overload. |
| **Osteopontin** (SPP1, OPN) | SPP1, OSTP, BNSP, BSPI, ETA-1, OPN, secreted phosphoprotein 1, Osteopontin | (36, 37) | Tier 1 | Osteopontin plays a central role in the remodeling response to [myocardial infarction](https://en.wikipedia.org/wiki/Myocardial_infarction) and dramatically upregulated cardiomyopathies. It stimulates a wide range of physiological changes in the [myocardium](https://en.wikipedia.org/wiki/Myocardium), including [angiogenesis](https://en.wikipedia.org/wiki/Angiogenesis), local production of [cytokines](https://en.wikipedia.org/wiki/Cytokines), differentiation of [myofibroblasts](https://en.wikipedia.org/wiki/Myofibroblasts), and [hypertrophy](https://en.wikipedia.org/wiki/Hypertrophy) of [cardiomyocytes](https://en.wikipedia.org/wiki/Cardiomyocytes). Taken together, these processes lead to an increased risk of [heart failure](https://en.wikipedia.org/wiki/Heart_failure). |
| **Peptidoglycan recognition protein 1** (PGLYRP1) | PGLYRP1, PGLYRP, PGRP,PGRP1, PGRP-S, PGRPS, TAG7, TNFSF3L, peptidoglycan recognition protein 1 | (38-40) | Tier 3 | Peptidoglycan recognition protein 1 promotes inflammation through the activation of innate immune mechanisms. The protein is a biomarker for coronary artery disease and heart failure. |
| **Pentraxin-related protein PTX3** (PTX3) | PTX3, TNFAIP5, TSG-14, pentraxin 3 | (41, 42) | Tier 3 | Pentraxin 3 is involved in vascular inflammation and endothelial dysfunction. Plasma levels rise rapidly in acute myocardial infarction, heart failure, and cardiac arrest, reflecting the extent of tissue damage and predicting the risk of mortality. |
| **Sclerostin** (SOST) | SOST, CDD, CHGA, SOST1, VBCH, DAND6, sclerostin, Sclerostin | (43-46) | Tier 1 | Sclerostin is an antagonizing regulator of Wnt/β-catenin signaling, and, in turn, the Wnt pathway is involved in cardiovascular remodeling, and inhibiting sclerostin could promote vascular calciﬁcation and the risk of cardiovascular events. SOST genetic variants related to lower circulating sclerostin also confer a higher risk of myocardial infarction, coronary revascularization, major adverse cardiovascular events, diabetes mellitus, high blood pressure, and central adiposity. |
| **Metalloproteinase inhibitor 4** (TIMP4) | TIMP4, TIMP metallopeptidase inhibitor 4, TIMP-4 | (47-49) | Tier 3 | TIMP-4 regulates platelet aggregation and is a negative regulator of atherosclerosis. |
| **Urokinase plasminogen activator surface receptor** (U-PAR) | PLAUR, CD87, U-PAR, UPAR, URKR, plasminogen activator, urokinase receptor | (50-52) | Tier 2 | Soluble urokinase receptor is a potential biomarker for acute coronary syndrome, and the protein predicts cardiovascular death and may enhance prognostication beyond traditional risk factors in apparently healthy individuals. |
| **Insulin-like growth factor-binding protein 1** (IGFBP-1) | IGFBP1, IGFBPL1, AFBP, IBP1, IGF-BP25, PP12, hIGFBP-1, insulin-like growth factor binding protein 1 | (53, 54) | Tier 1 | IGFBP1 improves insulin sensitivity and can predict future cardiovascular mortality and morbidity; serves as a marker of hyperinsulinemia, which precedes the subsequent development of insulin resistance and CVD |
| **Meteorin-like protein** (METRNL) | METRNL, Meteorin-like/Meteorin-Beta (Metrnl)/IL-41, subfatin, cometin | (55, 56) | Tier 2 | Produced by skeletal muscle and fat tissue, meteorin-like protein promotes myocardial repair. It improves glucose metabolism, and circulating meteorin-like protein is a new biomarker of heart failure with an independent prognostic value. |
| **N-terminal prohormone brain natriuretic peptide** (NT-proBNP) | BNPT, NPPB, NT-proBNP, NTPROBNP | (57, 58) | Tier 3 | An established biomarker of congestive heart failure and the protein is associated with [coronary artery disease](https://en.wikipedia.org/wiki/Coronary_artery_disease) and myocardial ischemia. |
| **C-C motif chemokine 15** (CCL15) | SCYA15, HCC-2, NCC-3, SCYL3, MIP-5, Lkn-1, MIP-1d, HMRP-2B | (59-61) | Tier 3 | CCL15 has angiogenic activity, and the protein participates together with other chemokines in atherogenesis and is related to intima-media thickness. |
| **Interleukin-6** (IL6) | IL6, BSF2, HGF, HSF, IFNB2, IL-6, BSF-2, CDF, IFN-beta-2, interleukin 6 | (62-64) | Tier 1 | Interleukin-6 is a crucial inflammation marker, and anti-inflammatory therapies targeting the central IL-6 signaling pathway are a promising treatment strategy to lessen the risk of MI. |
| **Leptin** (LEP) | LEP, LEPD, OB, OBS, leptin | (65-67) | Tier 3 | Leptin is a hormone mainly made by adipocytes, and its central role is likely to regulate long-term energy balance, but leptin is a critical regulator of the cardiovascular system. Leptin resistance increases the risk of cardiac dysfunction, acute myocardial infarction, stroke, and heart failure. |

**References**

1. Finan C, Gaulton A, Kruger FA, Lumbers RT, Shah T, Engmann J, et al. The druggable genome and support for target identification and validation in drug development. Science translational medicine. 2017;9(383).

2. Sangaralingham SJ, Kuhn M, Cannone V, Chen HH, Burnett JC. Natriuretic peptide pathways in heart failure: further therapeutic possibilities. Cardiovasc Res. 2023;118(18):3416-33.

3. Della Corte V, Pacinella G, Todaro F, Pecoraro R, Tuttolomondo A. The Natriuretic Peptide System: A Single Entity, Pleiotropic Effects. International journal of molecular sciences. 2023;24(11).

4. Yang J, Song QY, Niu SX, Chen HJ, Petersen RB, Zhang Y, et al. Emerging roles of angiopoietin-like proteins in inflammation: Mechanisms and potential as pharmacological targets. J Cell Physiol. 2022;237(1):98-117.

5. Hato T, Tabata M, Oike Y. The role of angiopoietin-like proteins in angiogenesis and metabolism. Trends Cardiovasc Med. 2008;18(1):6-14.

6. Santulli G. Angiopoietin-Like Proteins: A Comprehensive Look. Frontiers in Endocrinology. 2014;5.

7. Han SR, Momeni A, Strach K, Suriyaphol P, Fenske D, Paprotka K, et al. Enzymatically modified LDL induces cathepsin H in human monocytes: potential relevance in early atherogenesis. Arterioscler Thromb Vasc Biol. 2003;23(4):661-7.

8. Zhang X, Luo S, Wang M, Shi GP. Cysteinyl cathepsins in cardiovascular diseases. Biochim Biophys Acta Proteins Proteom. 2020;1868(4):140360.

9. Gong D, Zhang Q, Chen LY, Yu XH, Wang G, Zou J, et al. Coiled-coil domain-containing 80 accelerates atherosclerosis development through decreasing lipoprotein lipase expression via ERK1/2 phosphorylation and TET2 expression. Eur J Pharmacol. 2019;843:177-89.

10. Priyanka PP, Yenugu S. Coiled-Coil Domain-Containing (CCDC) Proteins: Functional Roles in General and Male Reproductive Physiology. Reproductive Sciences. 2021;28(10):2725-34.

11. Zhao T, Su Z, Li Y, Zhang X, You Q. Chitinase-3 like-protein-1 function and its role in diseases. Signal Transduct Target Ther. 2020;5(1):201.

12. Yeo IJ, Lee CK, Han SB, Yun J, Hong JT. Roles of chitinase 3-like 1 in the development of cancer, neurodegenerative diseases, and inflammatory diseases. Pharmacol Ther. 2019;203:107394.

13. Wang X, Hu Y, Wang Y, Shen D, Tao G. CLEC5A knockdown protects against cardiac dysfunction after myocardial infarction by suppressing macrophage polarization, NLRP3 inflammasome activation, and pyroptosis. Biochem Cell Biol. 2021;99(5):655-65.

14. Sung PS, Chang WC, Hsieh SL. CLEC5A: A Promiscuous Pattern Recognition Receptor to Microbes and Beyond. Adv Exp Med Biol. 2020;1204:57-73.

15. Yasmin T, Adiba M, Saba AA, Nabi AN. In Silico Design of siRNAs for Silencing CLEC5A Receptor as a Potential Therapeutic Approach Against Dengue and Japanese Encephalitis Virus Infection in Human. Bioinformatics and Biology Insights. 2022;16:11779322221142122.

16. Satomi-Kobayashi S, Ueyama T, Mueller S, Toh R, Masano T, Sakoda T, et al. Deficiency of nectin-2 leads to cardiac fibrosis and dysfunction under chronic pressure overload. Hypertension. 2009;54(4):825-31.

17. Kenagy RD, Plaas AH, Wight TN. Versican degradation and vascular disease. Trends Cardiovasc Med. 2006;16(6):209-15.

18. Wight TN, Kang I, Evanko SP, Harten IA, Chang MY, Pearce OMT, et al. Versican-A Critical Extracellular Matrix Regulator of Immunity and Inflammation. Front Immunol. 2020;11:512.

19. Theofilatos K, Stojkovic S, Hasman M, van der Laan SW, Baig F, Barallobre-Barreiro J, et al. Proteomic Atlas of Atherosclerosis: The Contribution of Proteoglycans to Sex Differences, Plaque Phenotypes, and Outcomes. Circ Res. 2023;133(7):542-58.

20. Makki N, Thiel KW, Miller FJ, Jr. The epidermal growth factor receptor and its ligands in cardiovascular disease. International journal of molecular sciences. 2013;14(10):20597-613.

21. Gekle M, Dubourg V, Schwerdt G, Benndorf RA, Schreier B. The role of EGFR in vascular AT1R signaling: From cellular mechanisms to systemic relevance. Biochem Pharmacol. 2023;217:115837.

22. Anand K, Ensor J, Trachtenberg B, Bernicker EH. Osimertinib-Induced Cardiotoxicity: A Retrospective Review of the FDA Adverse Events Reporting System (FAERS). JACC CardioOncol. 2019;1(2):172-8.

23. Iglesias P, Silvestre RA, Diez JJ. Growth differentiation factor 15 (GDF-15) in endocrinology. Endocrine. 2023;81(3):419-31.

24. Wollert KC, Kempf T, Wallentin L. Growth Differentiation Factor 15 as a Biomarker in Cardiovascular Disease. Clin Chem. 2017;63(1):140-51.

25. Boughanem H, Yubero-Serrano EM, Lopez-Miranda J, Tinahones FJ, Macias-Gonzalez M. Potential Role of Insulin Growth-Factor-Binding Protein 2 as Therapeutic Target for Obesity-Related Insulin Resistance. International journal of molecular sciences. 2021;22(3).

26. Hoeflich A, Wu M, Mohan S, Föll Jr, Wanke Rd, Froehlich T, et al. Overexpression of Insulin-Like Growth Factor-Binding Protein-2 in Transgenic Mice Reduces Postnatal Body Weight Gain. Endocrinology. 1999;140(12):5488-96.

27. Brismar K, Hilding A, Ansurudeen I, Flyvbjerg A, Frystyk J, Ostenson CG. Adiponectin, IGFBP-1 and -2 are independent predictors in forecasting prediabetes and type 2 diabetes. Front Endocrinol (Lausanne). 2022;13:1092307.

28. Lau ES, Paniagua SM, Zarbafian S, Hoffman U, Long MT, Hwang SJ, et al. Cardiovascular Biomarkers of Obesity and Overlap With Cardiometabolic Dysfunction. Journal of the American Heart Association. 2021;10(14):e020215.

29. Zhu M, Zheng C, Wei W, Everett L, Ginsburg D, Zhang B. Analysis of MCFD2- and LMAN1-deficient mice demonstrates distinct functions in vivo. Blood Adv. 2018;2(9):1014-21.

30. Zhang Y, Zhu M, Zheng C, Wei W, Emmer BT, Zhang B. LMAN1-MCFD2 complex is a cargo receptor for the ER-Golgi transport of alpha1-antitrypsin. The Biochemical journal. 2022;479(7):839-55.

31. Zhang B, Cunningham MA, Nichols WC, Bernat JA, Seligsohn U, Pipe SW, et al. Bleeding due to disruption of a cargo-specific ER-to-Golgi transport complex. Nat Genet. 2003;34(2):220-5.

32. Li S, Gao Y, Ma K, Li Y, Liu C, Yan Y, et al. Lipid-related protein NECTIN2 is an important marker in the progression of carotid atherosclerosis: An intersection of clinical and basic studies. J Transl Int Med. 2021;9(4):294-306.

33. Kobecki J, Gajdzis P, Mazur G, Chabowski M. Nectins and Nectin-like Molecules in Colorectal Cancer: Role in Diagnostics, Prognostic Values, and Emerging Treatment Options: A Literature Review. Diagnostics (Basel). 2022;12(12).

34. Russo E, Runge P, Jahromi NH, Naboth H, Landtwing A, Montecchi R, et al. CD112 Regulates Angiogenesis and T Cell Entry into the Spleen. Cells. 2021;10(1).

35. Mizutani K, Miyata M, Shiotani H, Kameyama T, Takai Y. Nectin-2 in general and in the brain. Molecular and Cellular Biochemistry. 2022;477(1):167-80.

36. Shirakawa K, Sano M. Osteopontin in Cardiovascular Diseases. Biomolecules. 2021;11(7).

37. Lok ZSY, Lyle AN. Osteopontin in Vascular Disease. Arterioscler Thromb Vasc Biol. 2019;39(4):613-22.

38. Brownell NK, Khera A, de Lemos JA, Ayers CR, Rohatgi A. Association Between Peptidoglycan Recognition Protein-1 and Incident Atherosclerotic Cardiovascular Disease Events: The Dallas Heart Study. J Am Coll Cardiol. 2016;67(19):2310-2.

39. Han Y, Hua S, Chen Y, Yang W, Zhao W, Huang F, et al. Circulating PGLYRP1 Levels as a Potential Biomarker for Coronary Artery Disease and Heart Failure. J Cardiovasc Pharmacol. 2021;77(5):578-85.

40. Jin Y, Huang H, Shu X, Liu Z, Lu L, Dai Y, et al. Peptidoglycan Recognition Protein 1 Attenuates Atherosclerosis by Suppressing Endothelial Cell Adhesion. J Cardiovasc Pharmacol. 2021;78(4):615-21.

41. Zlibut A, Bocsan IC, Agoston-Coldea L. Pentraxin-3 and endothelial dysfunction. Adv Clin Chem. 2019;91:163-79.

42. Ristagno G, Fumagalli F, Bottazzi B, Mantovani A, Olivari D, Novelli D, et al. Pentraxin 3 in Cardiovascular Disease. Front Immunol. 2019;10:823.

43. Bovijn J, Krebs K, Chen CY, Boxall R, Censin JC, Ferreira T, et al. Evaluating the cardiovascular safety of sclerostin inhibition using evidence from meta-analysis of clinical trials and human genetics. Science translational medicine. 2020;12(549).

44. Fabre S, Funck-Brentano T, Cohen-Solal M. Anti-Sclerostin Antibodies in Osteoporosis and Other Bone Diseases. J Clin Med. 2020;9(11).

45. Langdahl BL, Hofbauer LC, Forfar JC. Cardiovascular Safety and Sclerostin Inhibition. J Clin Endocrinol Metab. 2021;106(7):1845-53.

46. De Mare A, Opdebeeck B, Neven E, D'Haese PC, Verhulst A. Sclerostin Protects Against Vascular Calcification Development in Mice. J Bone Miner Res. 2022;37(4):687-99.

47. Melendez-Zajgla J, Del Pozo L, Ceballos G, Maldonado V. Tissue inhibitor of metalloproteinases-4. The road less traveled. Mol Cancer. 2008;7:85.

48. Oikonen M, Wendelin-Saarenhovi M, Siitonen N, Sainio A, Juonala M, Kahonen M, et al. Tissue inhibitor of matrix metalloproteinases 4 (TIMP4) in a population of young adults: relations to cardiovascular risk markers and carotid artery intima-media thickness. The Cardiovascular Risk in Young Finns Study. Scand J Clin Lab Invest. 2012;72(7):540-6.

49. Gomez DE, Alonso DF, Yoshiji H, Thorgeirsson UP. Tissue inhibitors of metalloproteinases: structure, regulation and biological functions. Eur J Cell Biol. 1997;74(2):111-22.

50. Pruc M, Jannasz I, Swieczkowski D, Procyk G, Gasecka A, Rafique Z, et al. Diagnostic value of soluble urokinase-type plasminogen activator receptor in patients with acute coronary syndrome: A systematic review and meta-analysis. Cardiol J. 2023.

51. Olesen TB, Pareek M, Vishram-Nielsen JKK, Olsen MH. The influence of age and sex on the prognostic importance of traditional cardiovascular risk factors, selected circulating biomarkers and other markers of subclinical cardiovascular damage. Curr Opin Cardiol. 2023;38(1):21-31.

52. Frary CE, Blicher MK, Olesen TB, Stidsen JV, Greve SV, Vishram-Nielsen JK, et al. Circulating biomarkers for long-term cardiovascular risk stratification in apparently healthy individuals from the MONICA 10 cohort. European journal of preventive cardiology. 2020;27(6):570-8.

53. Brismar K, Hilding A, Lindgren B. Regulation of IGFBP-1 in humans. Prog Growth Factor Res. 1995;6(2-4):449-56.

54. Hoeflich A, David R, Hjortebjerg R. Current IGFBP-Related Biomarker Research in Cardiovascular Disease-We Need More Structural and Functional Information in Clinical Studies. Front Endocrinol (Lausanne). 2018;9:388.

55. Huynh K. Meteorin-like protein repairs the ischaemic heart via receptor KIT in endothelial cells. Nat Rev Cardiol. 2022;19(9):575.

56. Ruperez C, Ferrer-Curriu G, Cervera-Barea A, Florit L, Guitart-Mampel M, Garrabou G, et al. Meteorin-like/Meteorin-beta protects heart against cardiac dysfunction. J Exp Med. 2021;218(5).

57. Newman JD, Anthopolos R, Ruggles KV, Cornwell M, Reynolds HR, Bangalore S, et al. Biomarkers and cardiovascular events in patients with stable coronary disease in the ISCHEMIA Trials. Am Heart J. 2023;266:61-73.

58. Hjort M, Eggers KM, Lakic TG, Lindback J, Budaj A, Cornel JH, et al. Biomarker Concentrations and Their Temporal Changes in Patients With Myocardial Infarction and Nonobstructive Compared With Obstructive Coronary Arteries: Results From the PLATO Trial. Journal of the American Heart Association. 2023;12(1):e027466.

59. Dieden A, Malan L, Mels CMC, Lammertyn L, Wentzel A, Nilsson PM, et al. Exploring biomarkers associated with deteriorating vascular health using a targeted proteomics chip: The SABPA study. Medicine (Baltimore). 2021;100(20):e25936.

60. Aukrust P, Halvorsen B, Yndestad A, Ueland T, Oie E, Otterdal K, et al. Chemokines and cardiovascular risk. Arterioscler Thromb Vasc Biol. 2008;28(11):1909-19.

61. Hwang J, Kim CW, Son KN, Han KY, Lee KH, Kleinman HK, et al. Angiogenic activity of human CC chemokine CCL15 in vitro and in vivo. FEBS Lett. 2004;570(1-3):47-51.

62. Li Z, Lin C, Cai X, Hu S, Lv F, Yang W, et al. Anti-inflammatory therapies were associated with reduced risk of myocardial infarction in patients with established cardiovascular disease or high cardiovascular risks: A systematic review and meta-analysis of randomized controlled trials. Atherosclerosis. 2023;379:117181.

63. Papastamos C, Antonopoulos AS, Simantiris S, Koumallos N, Sagris M, Theofilis P, et al. Interleukin-6 Signaling in Atherosclerosis: From Molecular Mechanisms To Clinical Outcomes. Curr Top Med Chem. 2023;23(22):2172-83.

64. Mitsis A, Kadoglou NPE, Lambadiari V, Alexiou S, Theodoropoulos KC, Avraamides P, et al. Prognostic role of inflammatory cytokines and novel adipokines in acute myocardial infarction: An updated and comprehensive review. Cytokine. 2022;153:155848.

65. Poetsch MS, Strano A, Guan K. Role of Leptin in Cardiovascular Diseases. Front Endocrinol (Lausanne). 2020;11:354.

66. Koh KK, Park SM, Quon MJ. Leptin and cardiovascular disease: response to therapeutic interventions. Circulation. 2008;117(25):3238-49.

67. Soderberg S, Colquhoun D, Keech A, Yallop J, Barnes EH, Pollicino C, et al. Leptin, but not adiponectin, is a predictor of recurrent cardiovascular events in men: results from the LIPID study. International journal of obesity. 2009;33(1):123-30.
