# Supplementary material for: Cardio-metabolic-related plasma proteins reveal biological links between cardiovascular diseases and fragility fractures: a cohort and Mendelian randomisation investigation
Source: eBioMedicine. 2025 Feb 6;113:105580. doi: 10.1016/j.ebiom.2025.105580 (PMC11848109; doi:10.1016/j.ebiom.2025.105580)
Supplement: Supplementary Figures S1–S11 [file mmc10.pdf]

Supplementary Figures S1-S11

Cardio-metabolic-related plasma proteins reveal biological links between cardiovascular diseases and fragility fractures: a cohort and Mendelian randomisation investigation

Karl Michaëlsson,[1] Rui Zheng,[2] John A. Baron,[1,3] Tove Fall,[4] Alicja Wolk,[5] Lars Lind,[2] Jonas Höijer,[1] Carl Brunius,[6] Eva Warensjö Lemming,[1] Olga E. Titova,[1] Bodil Svennblad,[1] Susanna C. Larsson,[1,5] Shuai Yuan,[5] Håkan Melhus,[7] Liisa Byberg,[1] Hannah L. Brooke[1]

[1] Medical Epidemiology, Department of Surgical Sciences, Uppsala University, Uppsala, Sweden.

[2] Clinical Epidemiology, Department of Medical Sciences, Uppsala University, Uppsala, Sweden.

[3] Department of Epidemiology, Gillings School of Global Public Health, University of North Carolina, Chapel Hill, NC, USA;

[4] Molecular Epidemiology, Department of Medical Sciences, Uppsala University, Uppsala, Sweden.

[5] Unit of Cardiovascular and Nutritional Epidemiology, Institute of Environmental Medicine, Karolinska Institutet, Stockholm, Sweden.

[6] Food and Nutrition Science, Department of Life Sciences, Chalmers University of Technology, Gothenburg, Sweden.

[7] Clinical Pharmacology, Department of Medical Sciences, Uppsala University, Uppsala, Sweden.

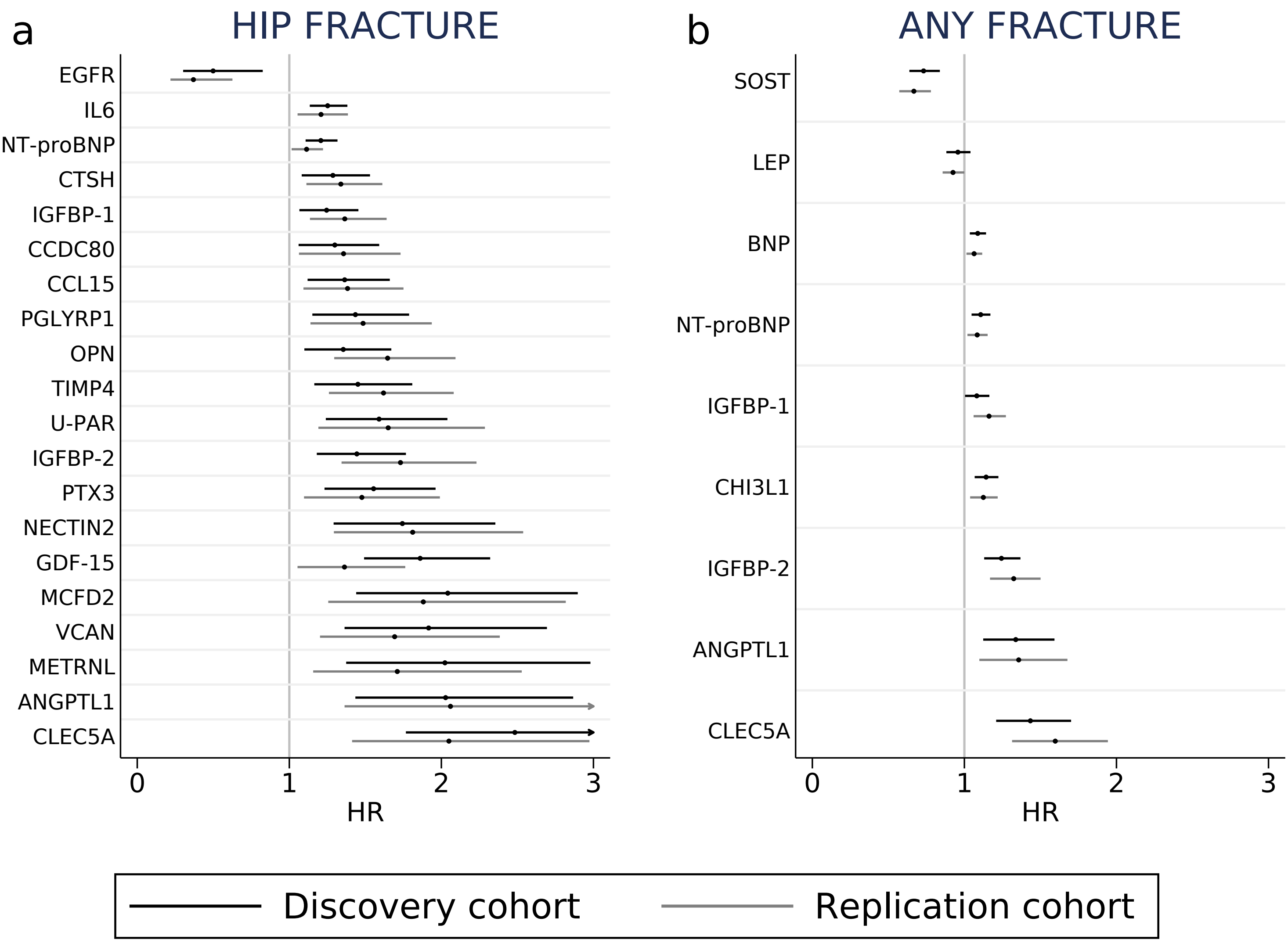

Supplementary Figure S1. Associations of 24 replicated proteins with risk of hip fracture (panel a) and any fracture (panel b) in the discovery (black markers, Uppsala, only women) and replication (grey markers, Västerås, women and men) cohorts. The hazard ratios (HRs) are adjusted for sex, age (continuous), body mass index (continuous), and height (continuous). The bars indicate 95% confidence intervals.

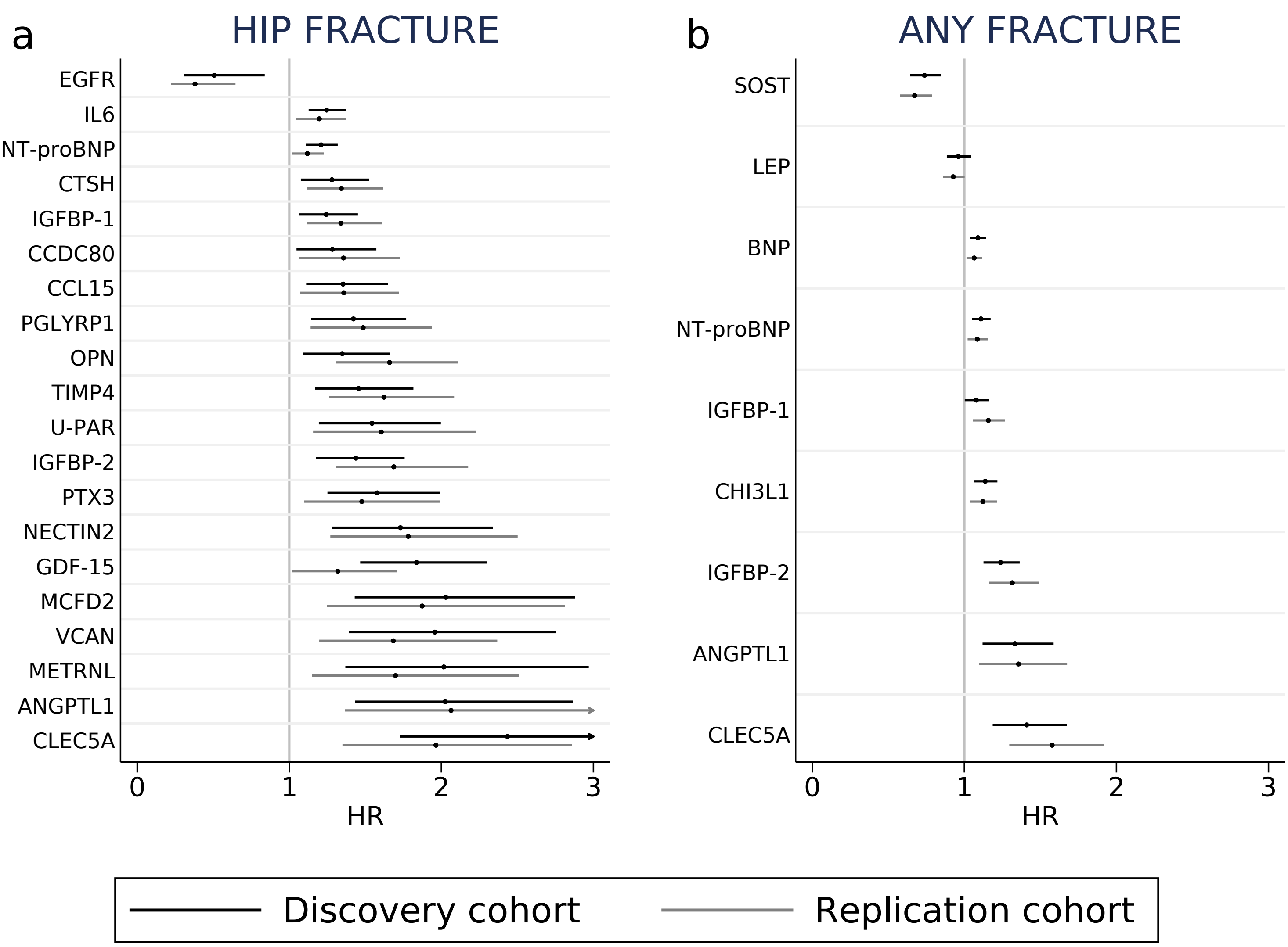

Supplementary Figure S2. Associations of 24 replicated proteins with risk of hip fracture (panel a) and any fracture (panel b) in the discovery (black markers, Uppsala, only women) and replication (grey markers, Västerås, women, and men) cohorts. The hazard ratios (HRs) are adjusted for sex, age (continuous), body mass index (continuous), height (continuous), and current smoking (yes/no). The bars indicate 95% confidence intervals.

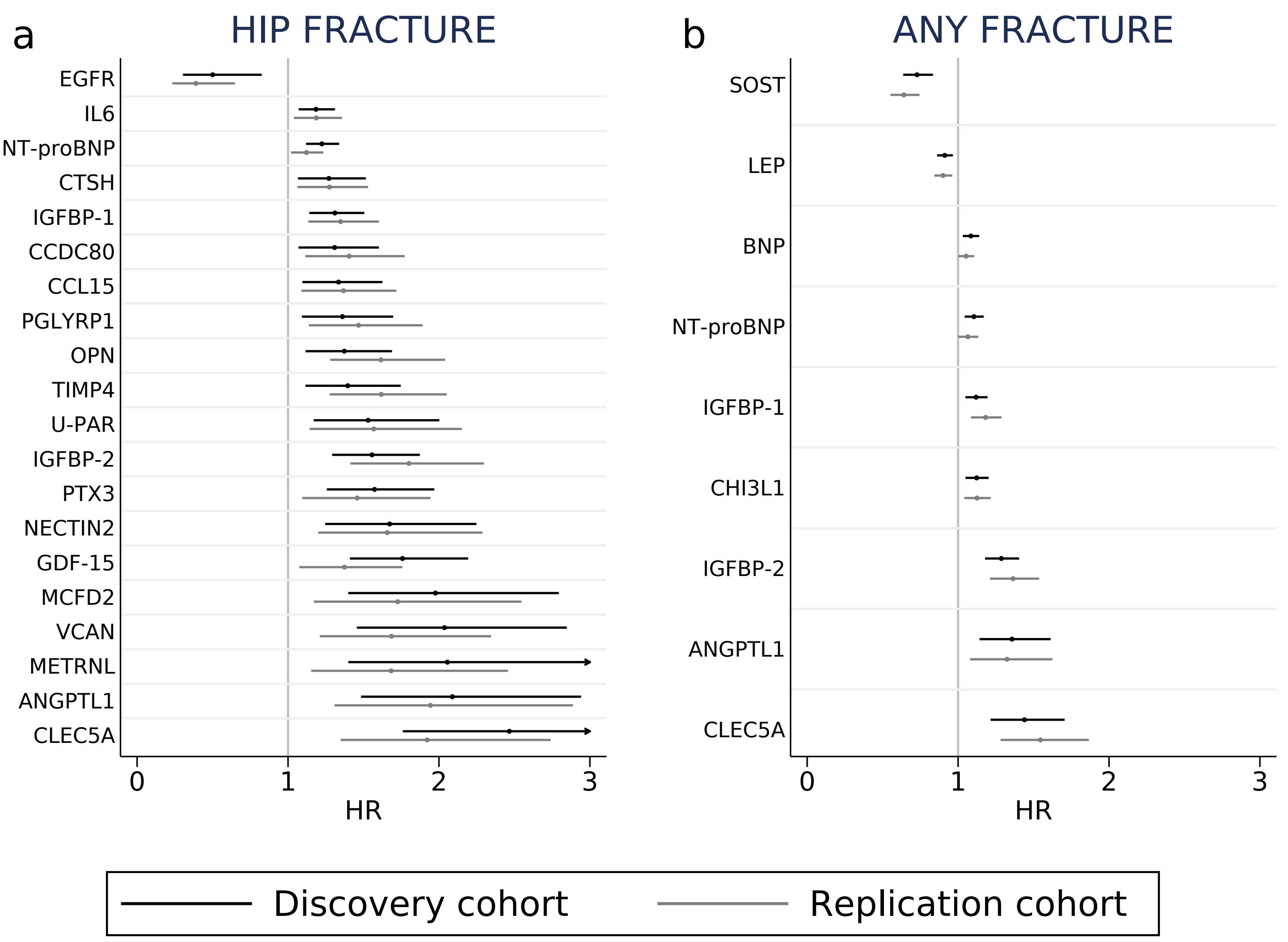

Supplementary Figure S3. Associations of 24 replicated proteins with risk of hip fracture (panel a) and any fracture (panel b) in the discovery (black markers, Uppsala, only women) and replication (grey markers, Västerås, women and men) cohorts. The hazard ratios (HRs) are adjusted for sex, age (continuous), and prevalent cardiovascular disease at baseline (ICD-10 codes I20-I25, I30-I52, and I60-I69, recorded in the Swedish National Patient Register: 0/1). The bars indicate 95% confidence intervals.

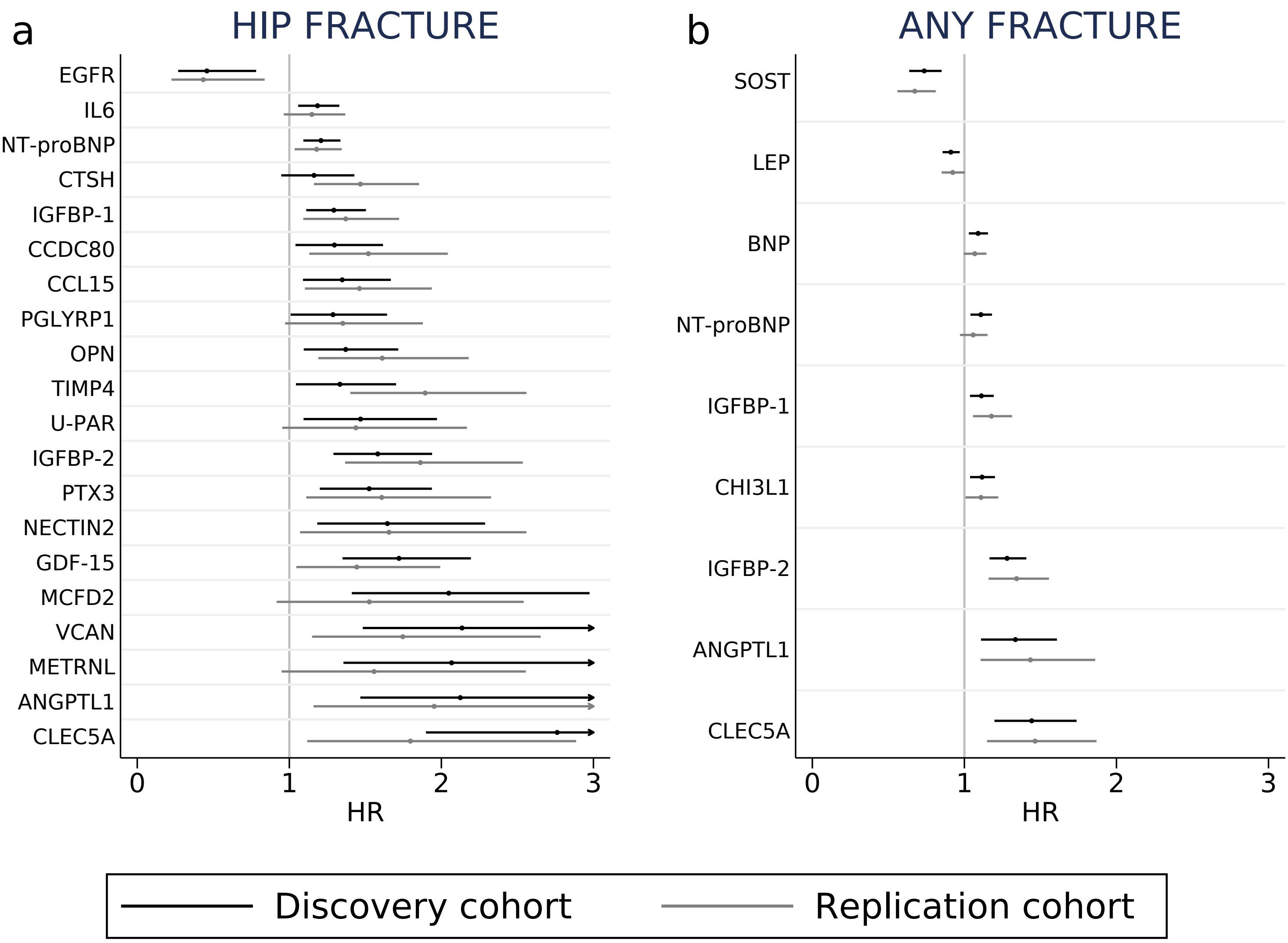

Supplementary Figure S4. Associations of 24 replicated proteins with risk of hip fracture (panel a) and any fracture (panel b) in the discovery (black markers, Uppsala, only women) and replication (grey markers, Västerås, women and men) cohorts after exclusion of those with prevalent cardiovascular disease (ICD-10 codes I20-I25, I30-I52, and I60-I69, recorded in the Swedish National Patient Register) at baseline. The hazard ratios (HRs) are adjusted for sex and age (continuous). The bars indicate 95% confidence intervals.

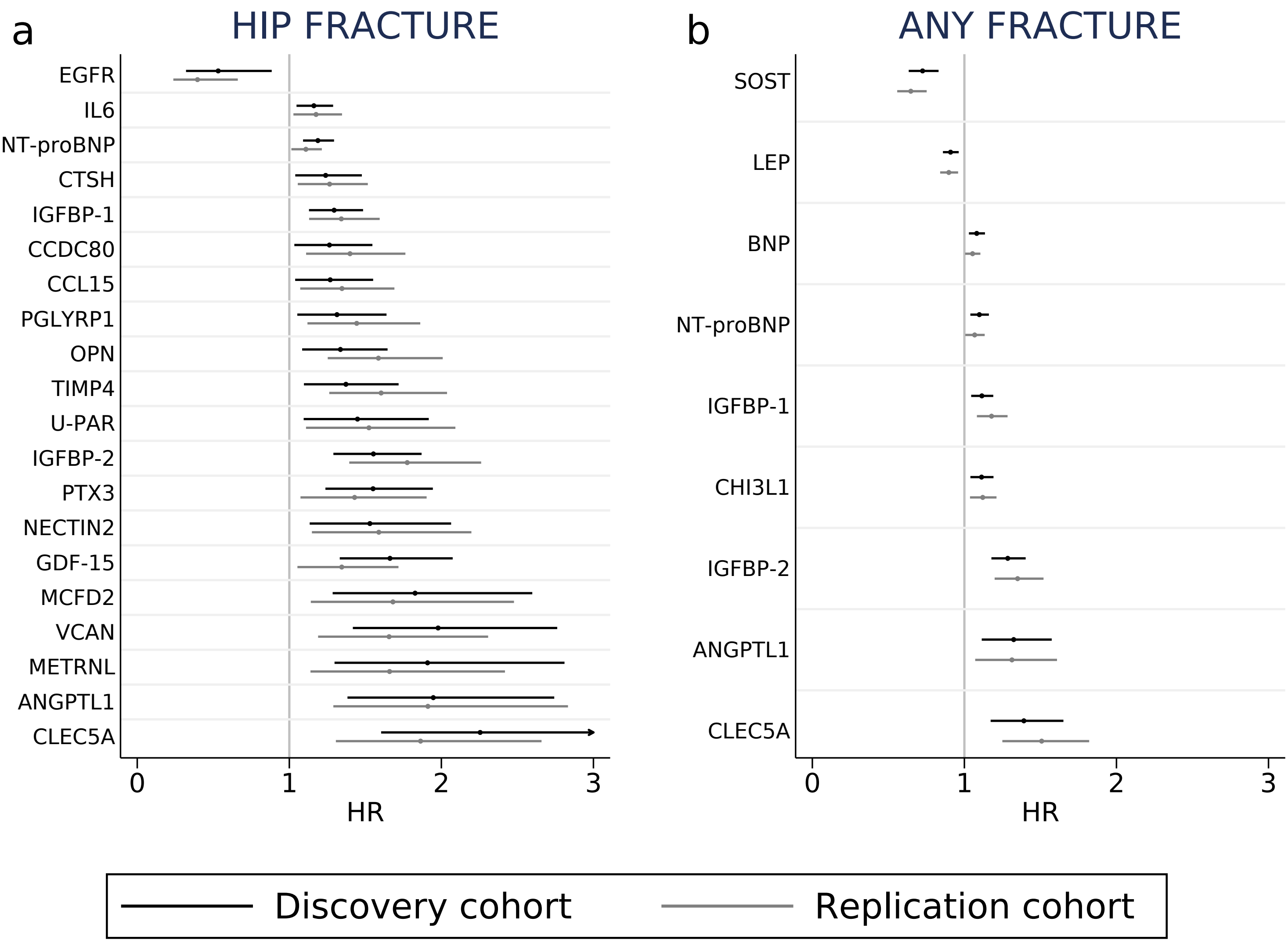

Supplementary Figure S5. Associations of 24 replicated proteins with risk of hip fracture (panel a) and any fracture (panel b) in the discovery (black markers, Uppsala, only women) and replication (grey markers, Västerås, women and men) cohorts. The hazard ratios (HRs) are adjusted for sex, age (continuous), and weighted Charlson comorbidity index at baseline (continuous). The bars indicate 95% confidence intervals.

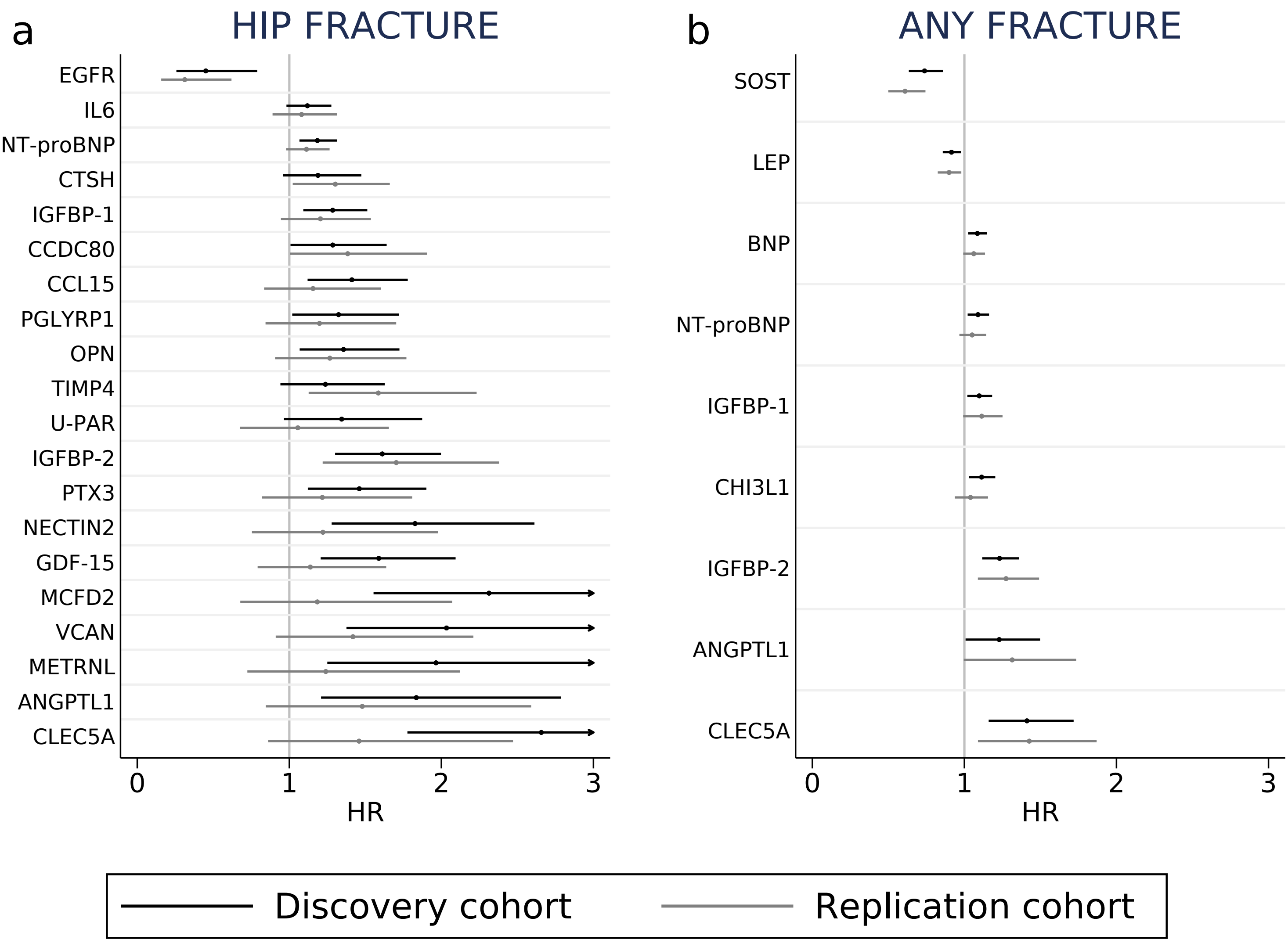

Supplementary Figure S6. Associations of 24 replicated proteins with risk of hip fracture (panel a) and any fracture (panel b) in the discovery (black markers, Uppsala, only women) and replication (grey markers, Västerås, women and men) cohorts after exclusion of those with any prevalent co-morbidity at baseline, as determined by Charlson comorbidity index (a value >0). The hazard ratios (HRs) are adjusted for sex and age (continuous). The bars indicate 95% confidence intervals.

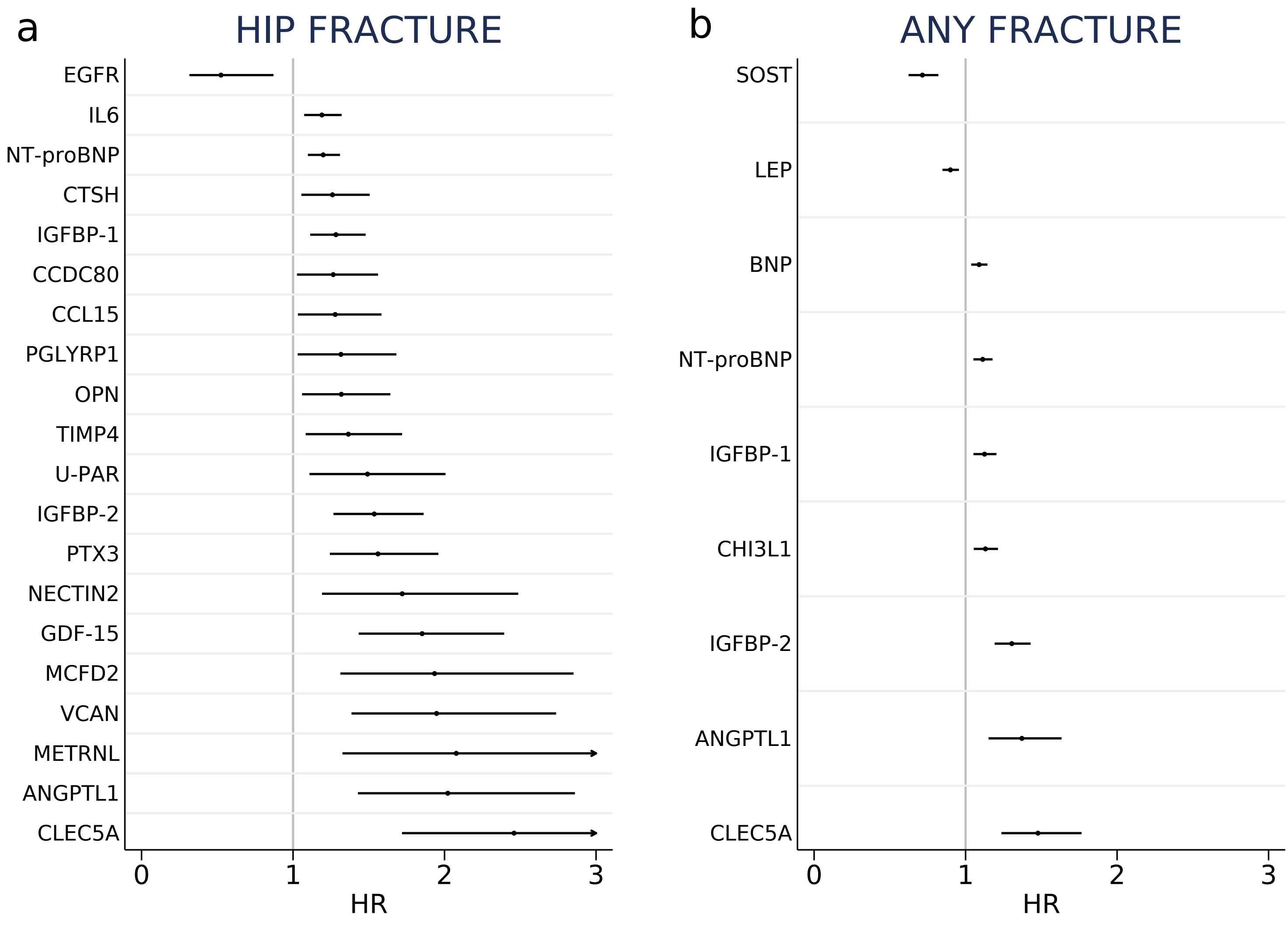

Supplementary Figure S7. Associations of 24 replicated proteins with risk of hip fracture (panel a) and any fracture (panel b) in the discovery cohort. The hazard ratios (HRs) are adjusted for age (continuous), estimated glomerular filtration rate (continuous, based on sex, weight, age, P-Cystatin C, and S-creatinine), and P-alanine aminotransferase (continuous). The bars indicate 95% confidence intervals.

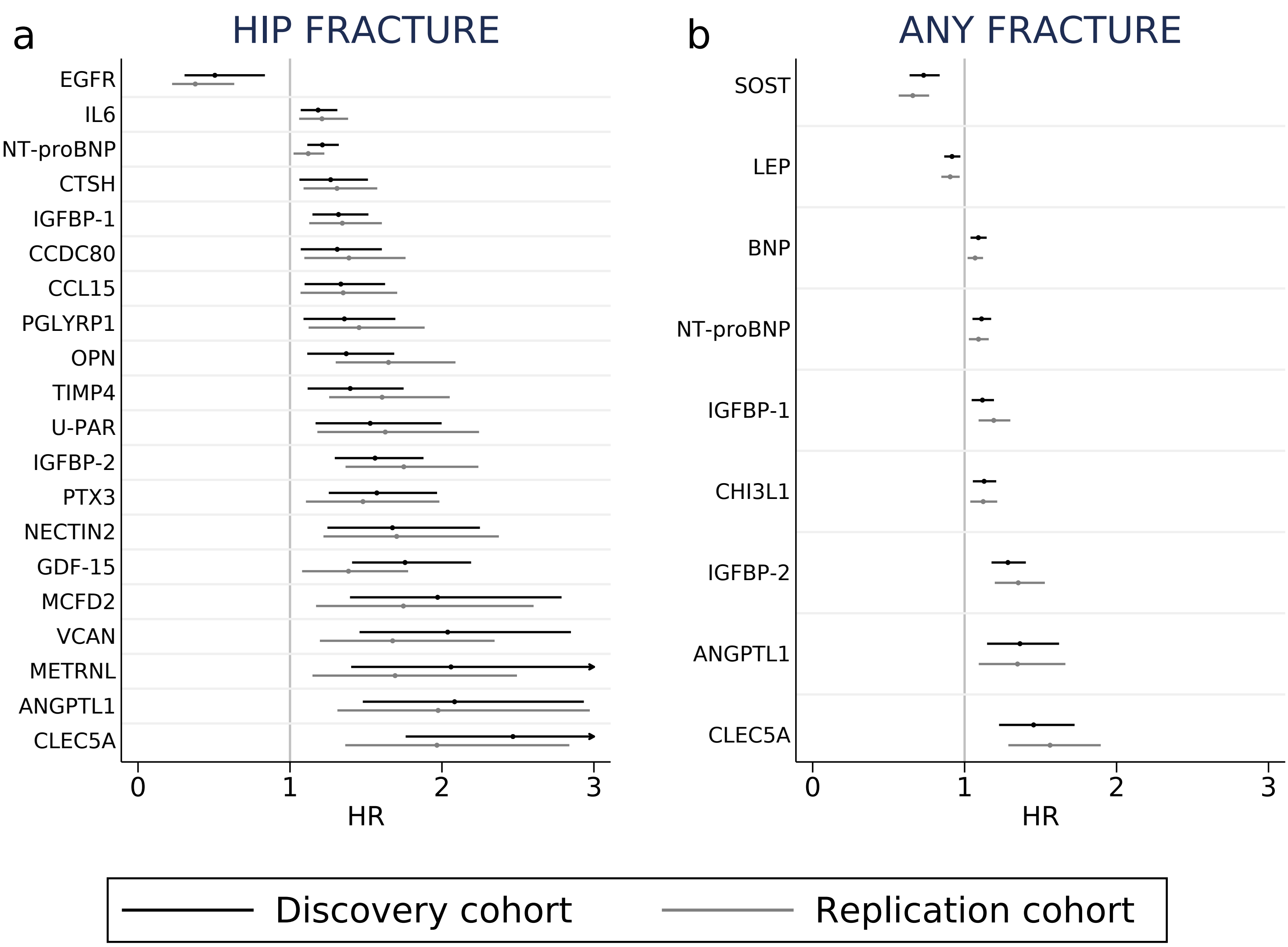

Supplementary Figure S8. Associations of 24 replicated proteins with risk of hip fracture (panel a) and any fracture (panel b) in the discovery (black markers, Uppsala, only women) and replication (grey markers, Västerås, women and men) cohorts. The hazard ratios (HRs) are adjusted for sex, age (continuous), and educational level (</= 9 years, 10-12 years, >12 years). The bars indicate 95% confidence intervals.

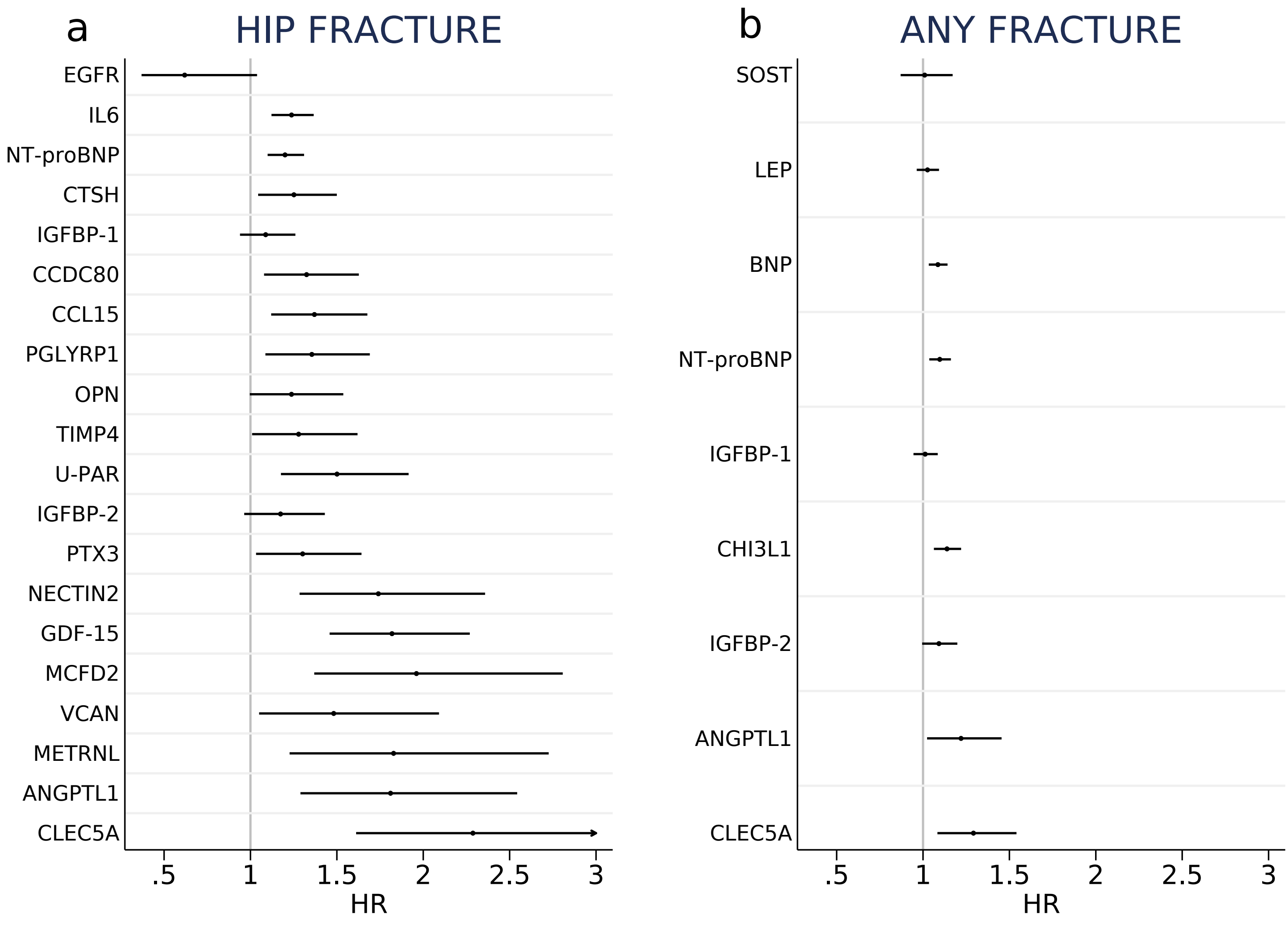

Supplementary Figure S9. Associations of 24 replicated proteins with risk of hip fracture (panel a) and any fracture (panel b) in the discovery cohort. The hazard ratios (HRs) are adjusted for age (continuous) and bone mineral density (continuous). The bars indicate 95% confidence intervals.

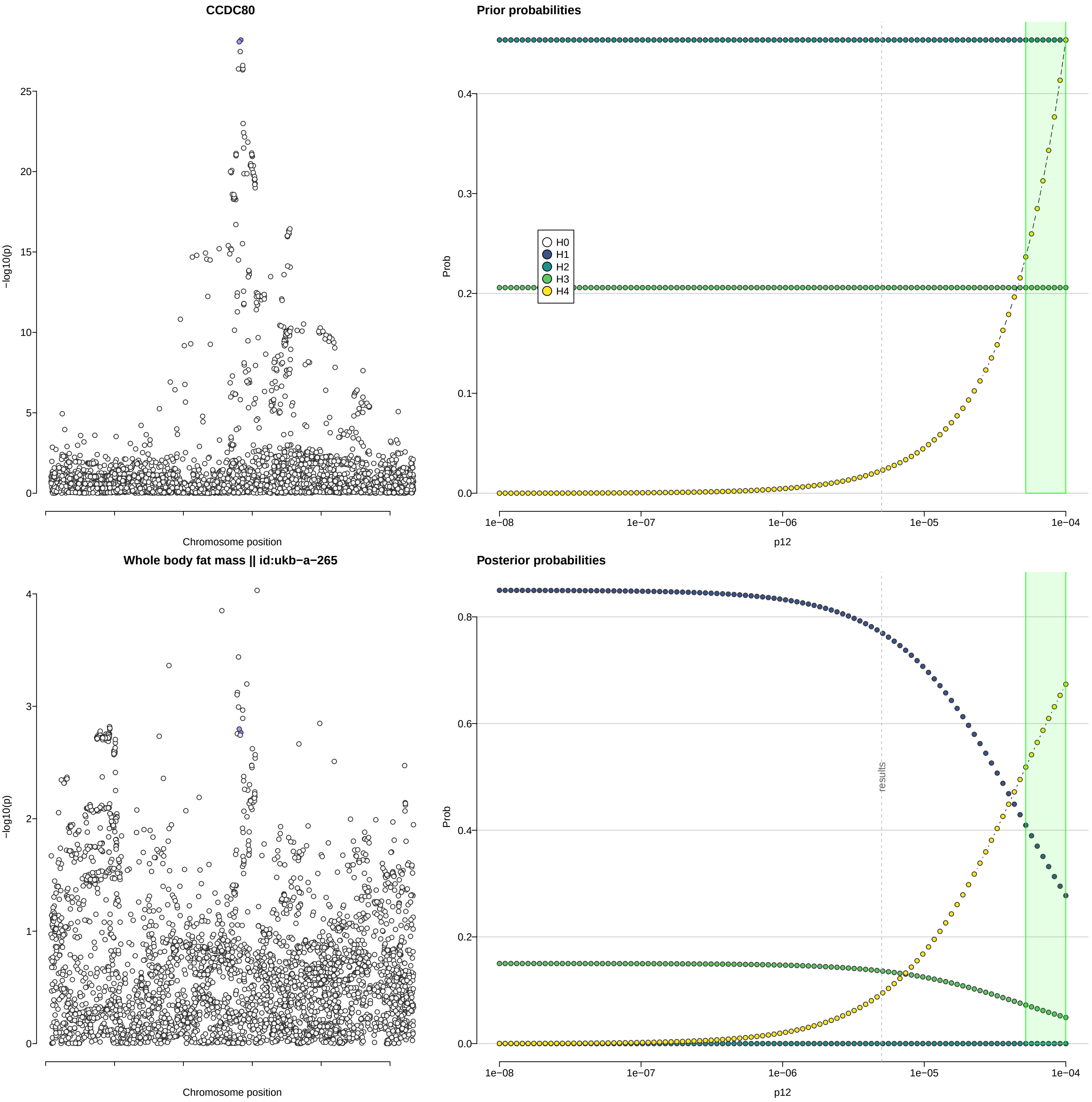

Supplementary Figure S10a. Locus zoom and prior sensitivity plots from colocalization analysis for Mendelian randomisation association of CCDC80 with whole body fat mass

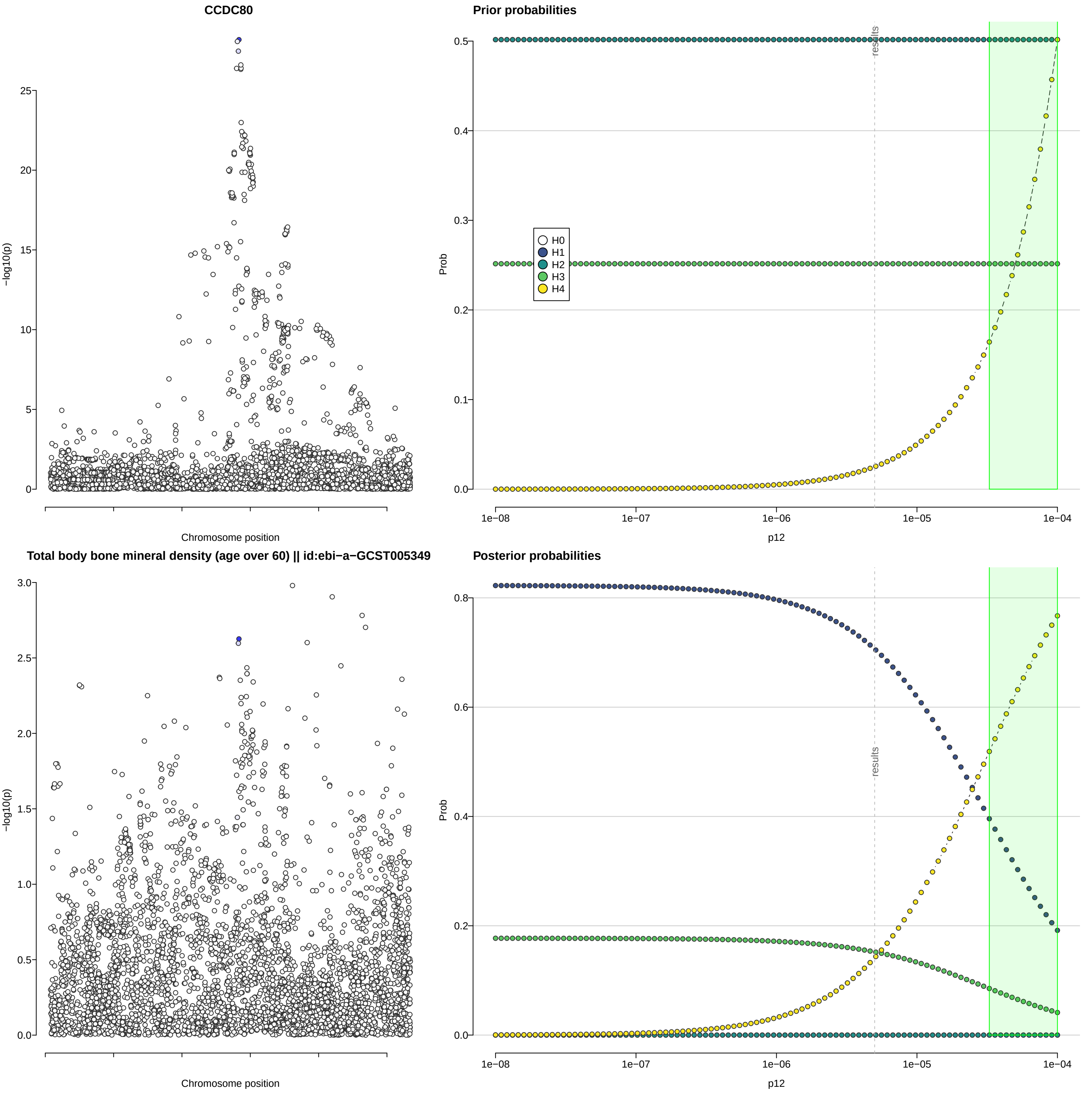

Supplementary Figure S10b. Locus zoom and prior sensitivity plots from colocalization analysis for Mendelian randomisation association of CCDC80 with total body bone mineral density (age over 60)

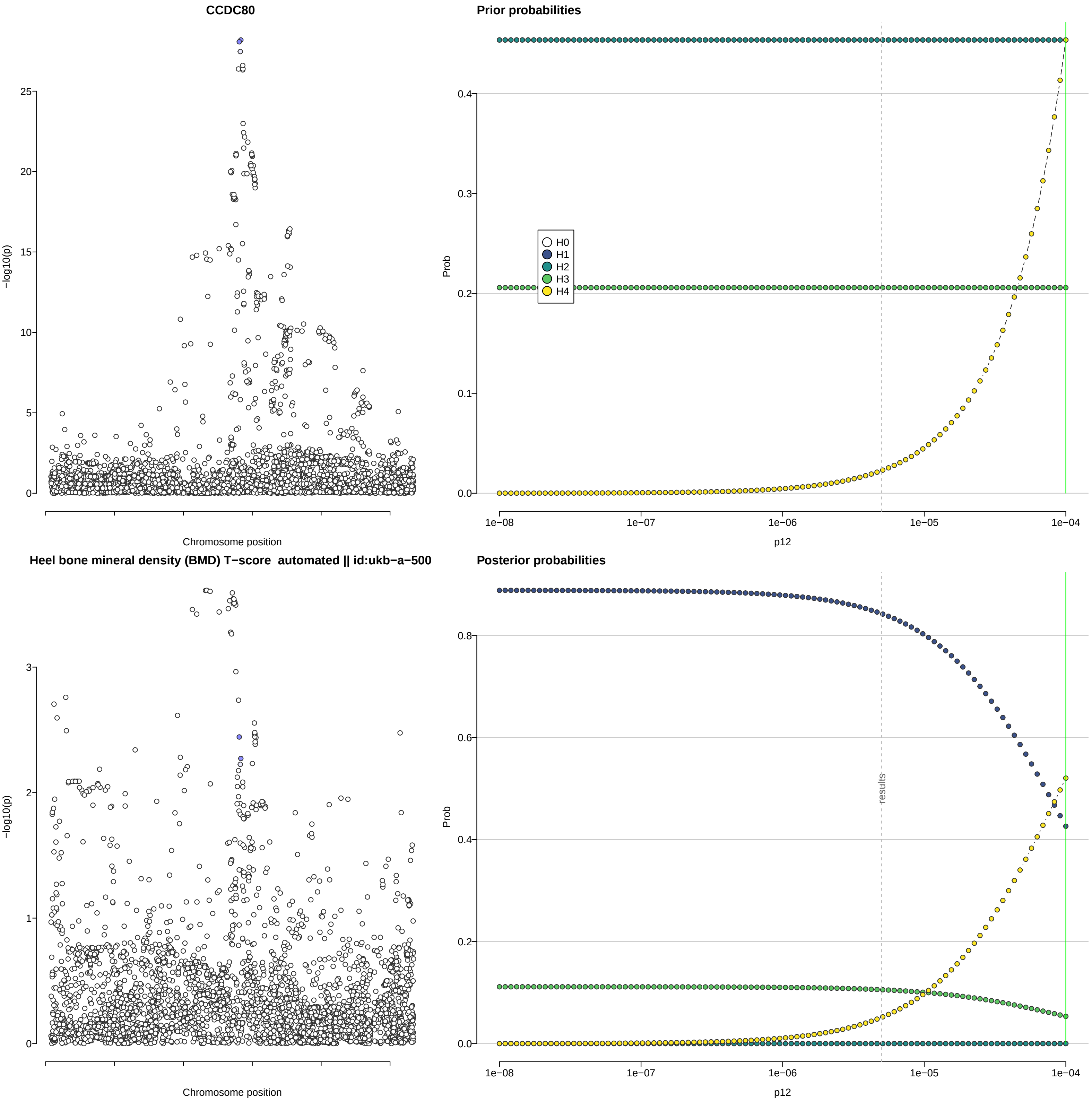

Supplementary Figure S10c. Locus zoom and prior sensitivity plots from colocalization analysis for Mendelian randomisation association of CCDC80 with heel bone mineral density (BMD) T-score automated

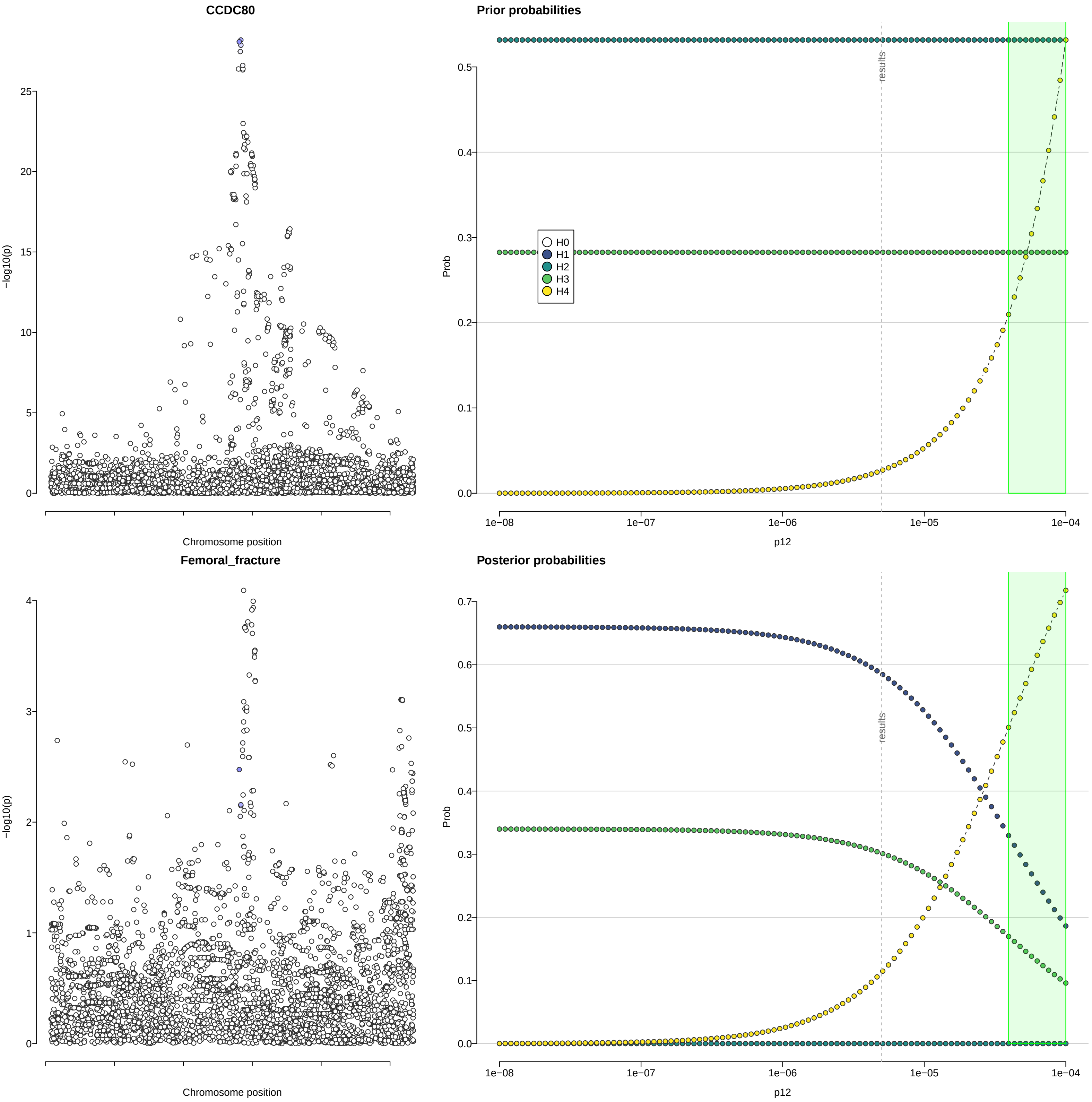

Supplementary Figure S10d. Locus zoom and prior sensitivity plots from colocalization analysis for Mendelian randomisation association of CCDC80 with femoral fracture

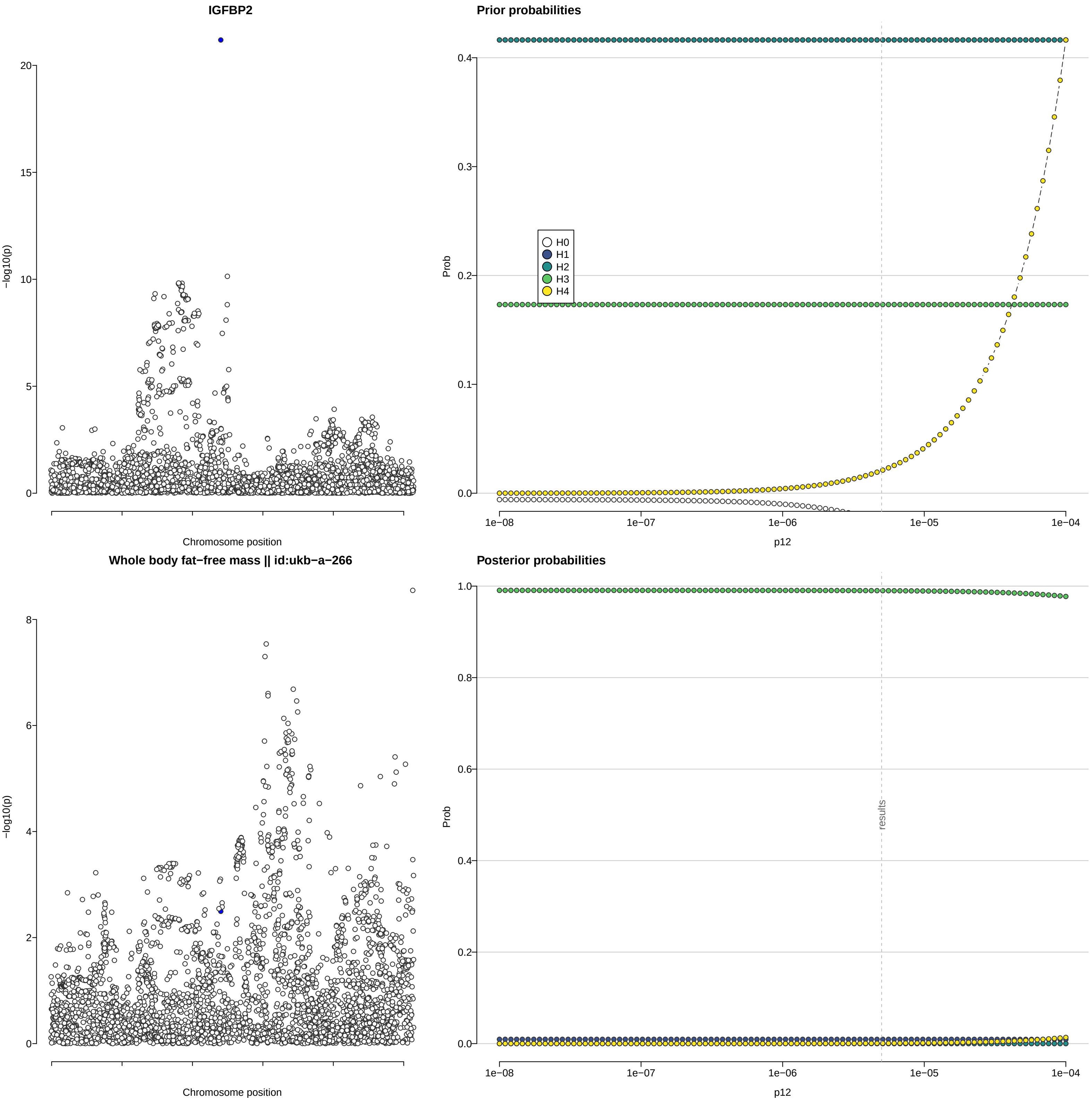

Supplementary Figure S10e. Locus zoom and prior sensitivity plots from colocalization analysis for Mendelian randomisation association of IGFBP2 with whole body fat-free mass

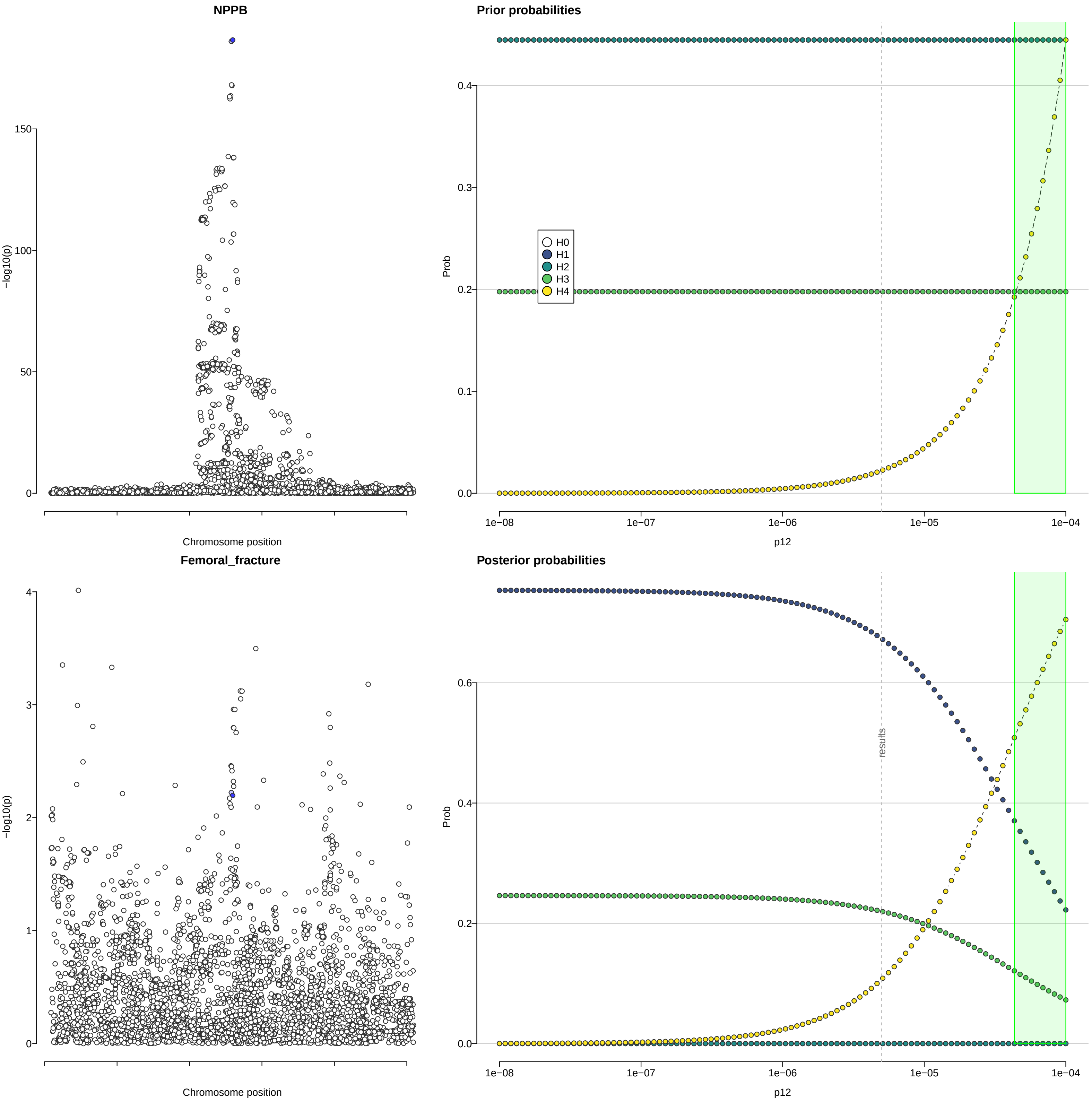

Supplementary Figure S10f. Locus zoom and prior sensitivity plots from colocalization analysis for Mendelian randomisation association of NPPB/BNP with femoral fracture

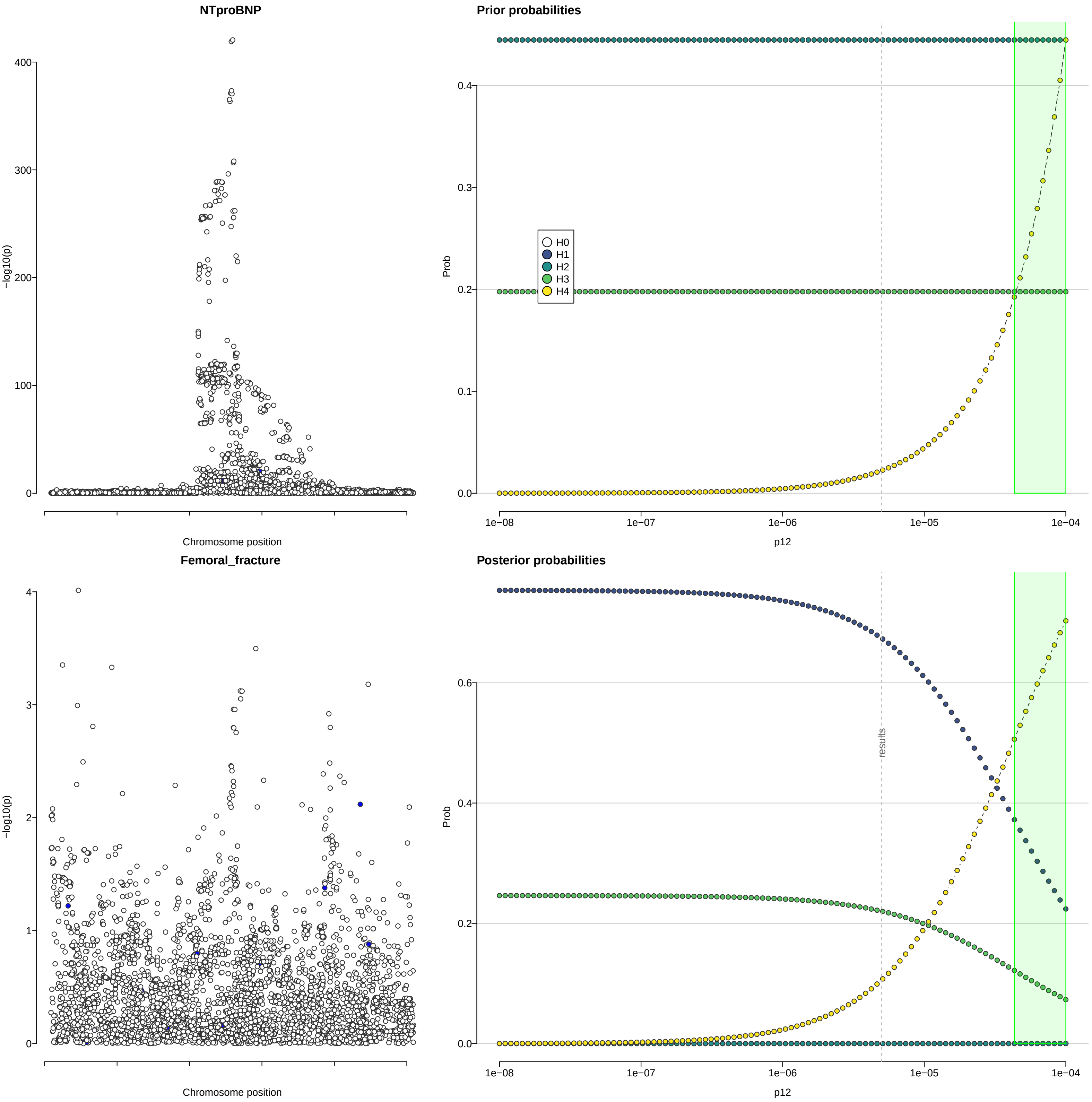

Supplementary Figure S10g. Locus zoom and prior sensitivity plots from colocalization analysis for Mendelian randomisation association of NTproBNP with femoral fracture

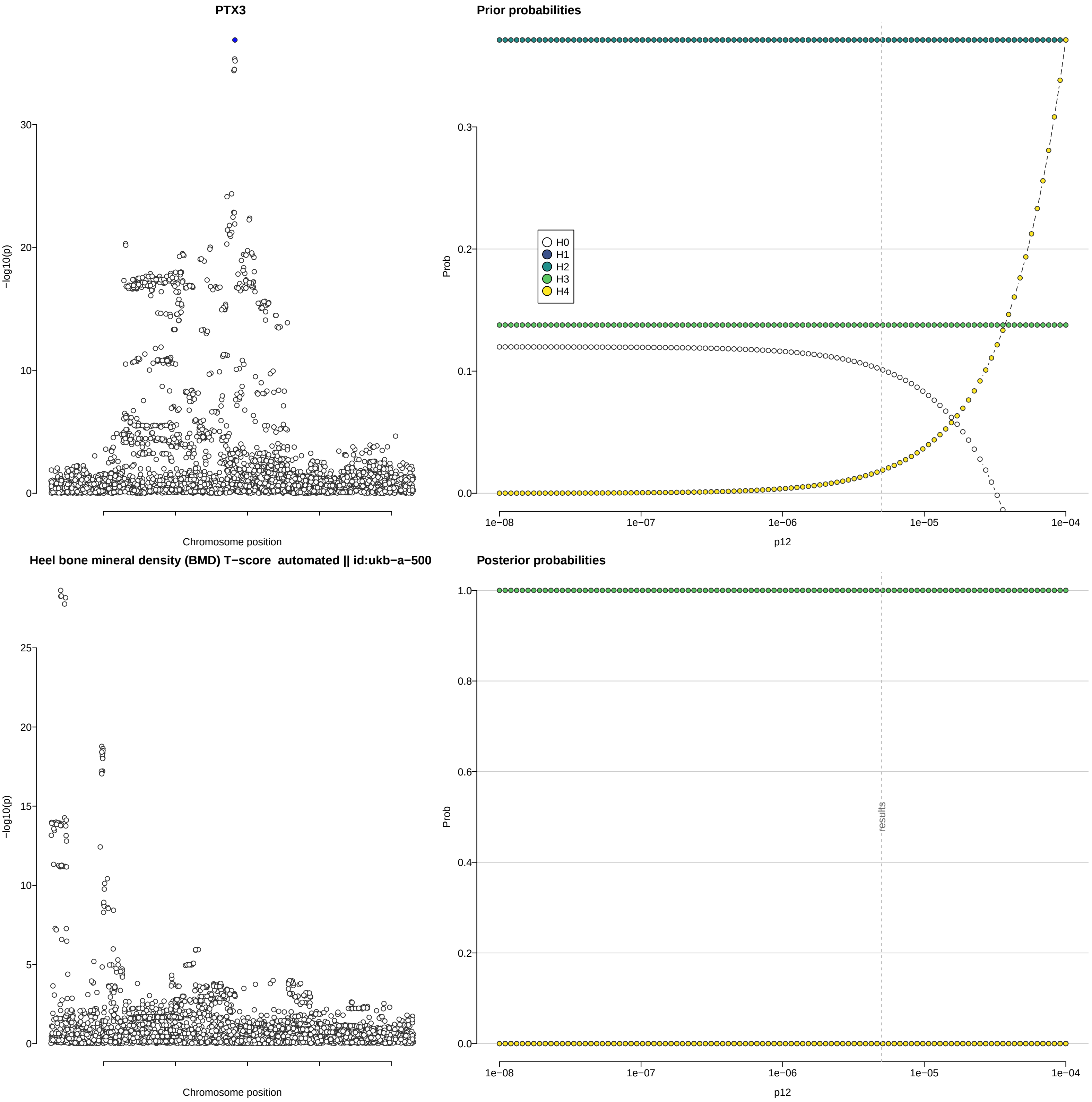

Supplementary Figure S10h. Locus zoom and prior sensitivity plots from colocalization analysis for Mendelian randomisation association of PTX3 with heel bone mineral density (BMD) T-score automated

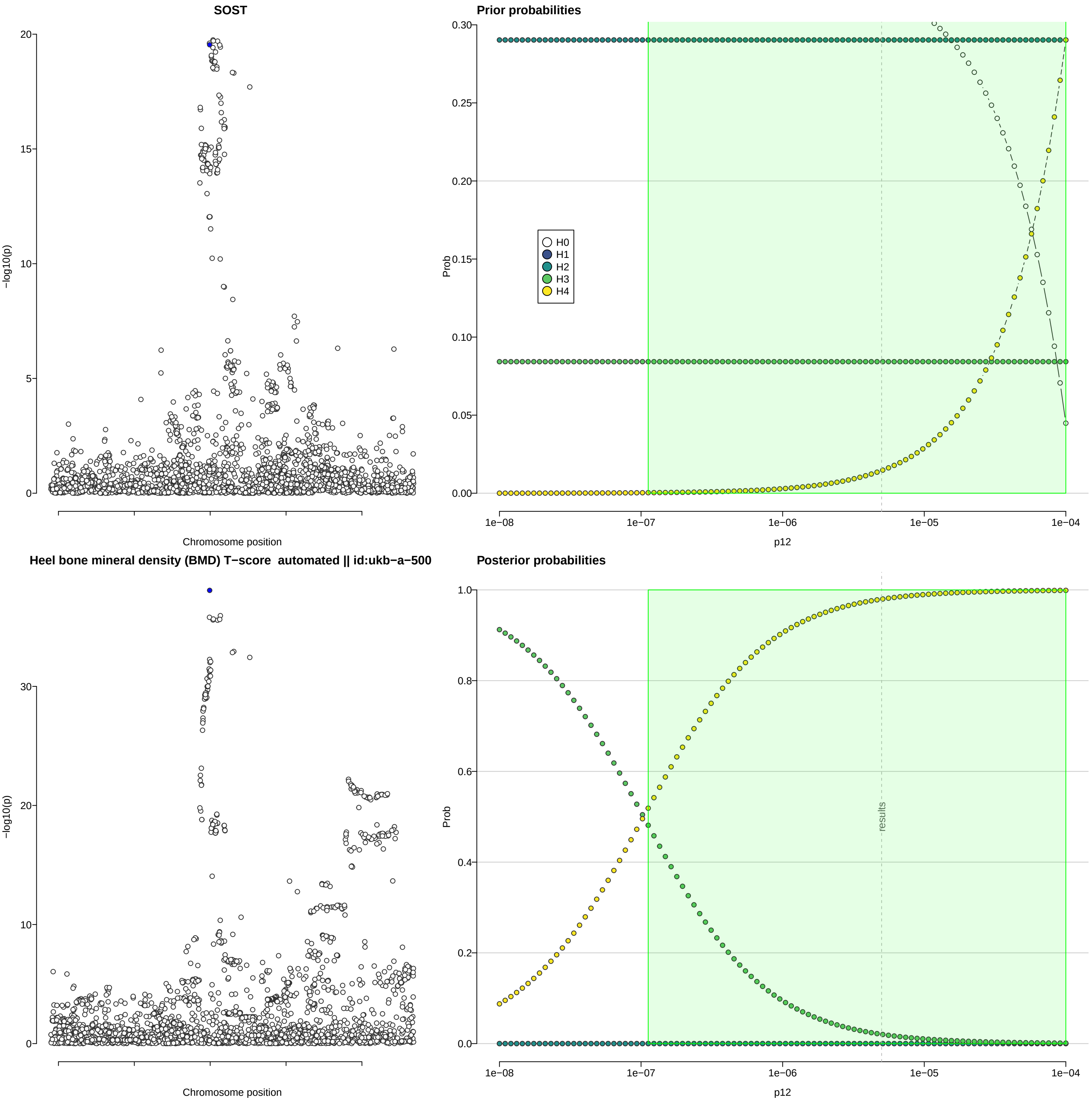

Supplementary Figure S10i. Locus zoom and prior sensitivity plots from colocalization analysis for Mendelian randomisation association of SOST with heel bone mineral density (BMD) T-score automated

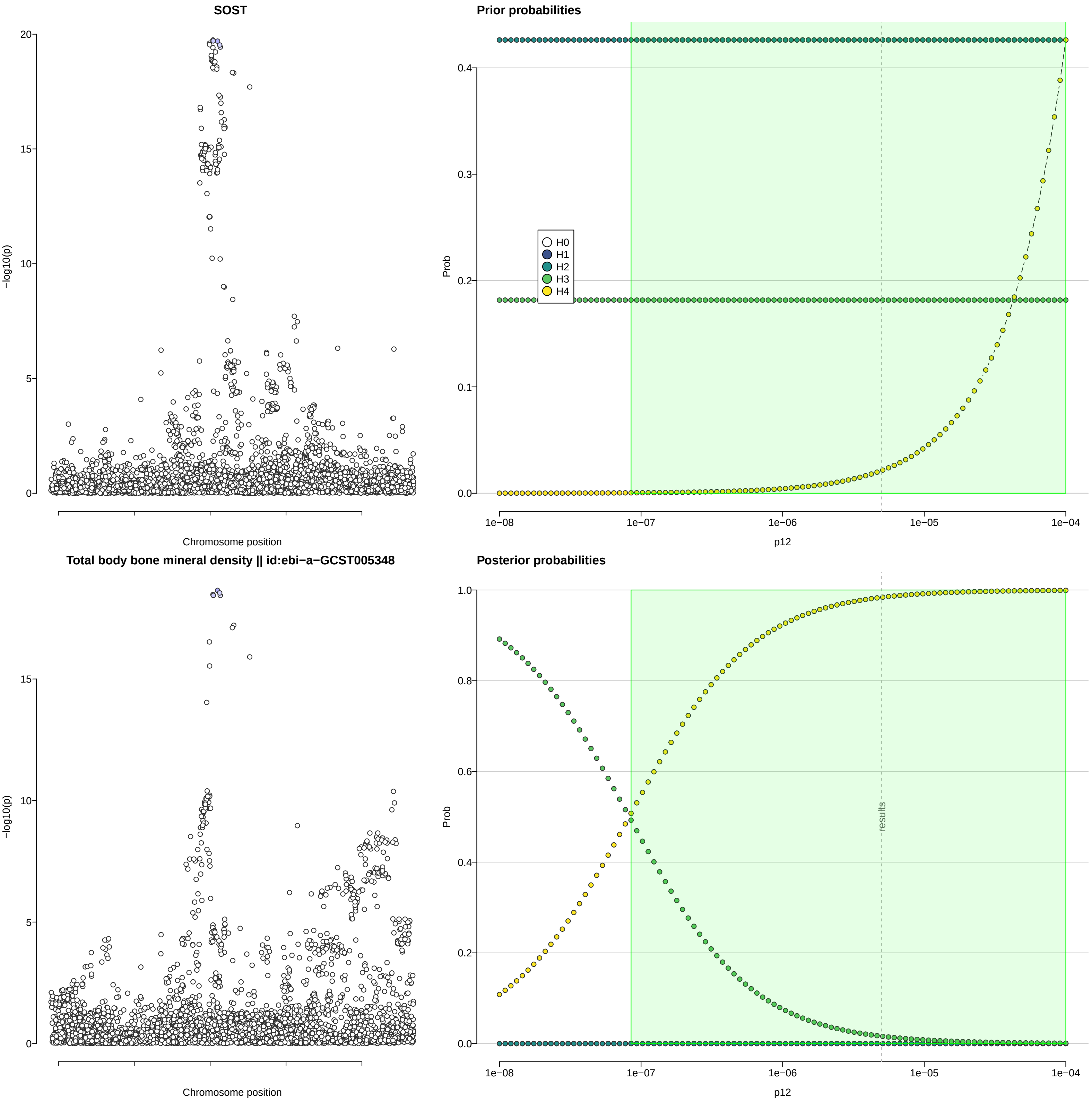

Supplementary Figure S10j. Locus zoom and prior sensitivity plots from colocalization analysis for Mendelian randomisation association of SOST with total body bone mineral density

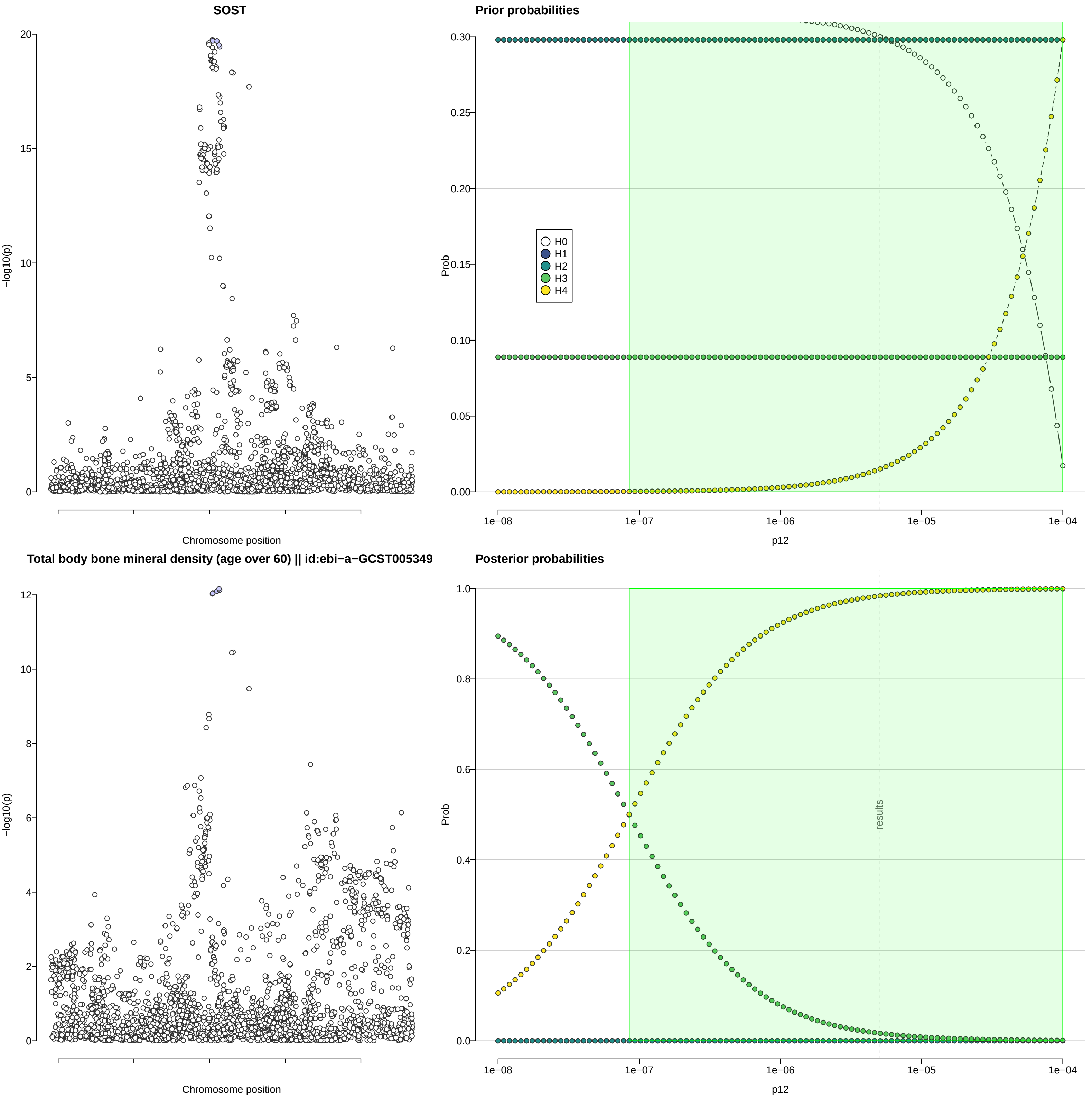

Supplementary Figure S10k. Locus zoom and prior sensitivity plots from colocalization analysis for Mendelian randomisation association of SOST with total body bone mineral density (age over 60)

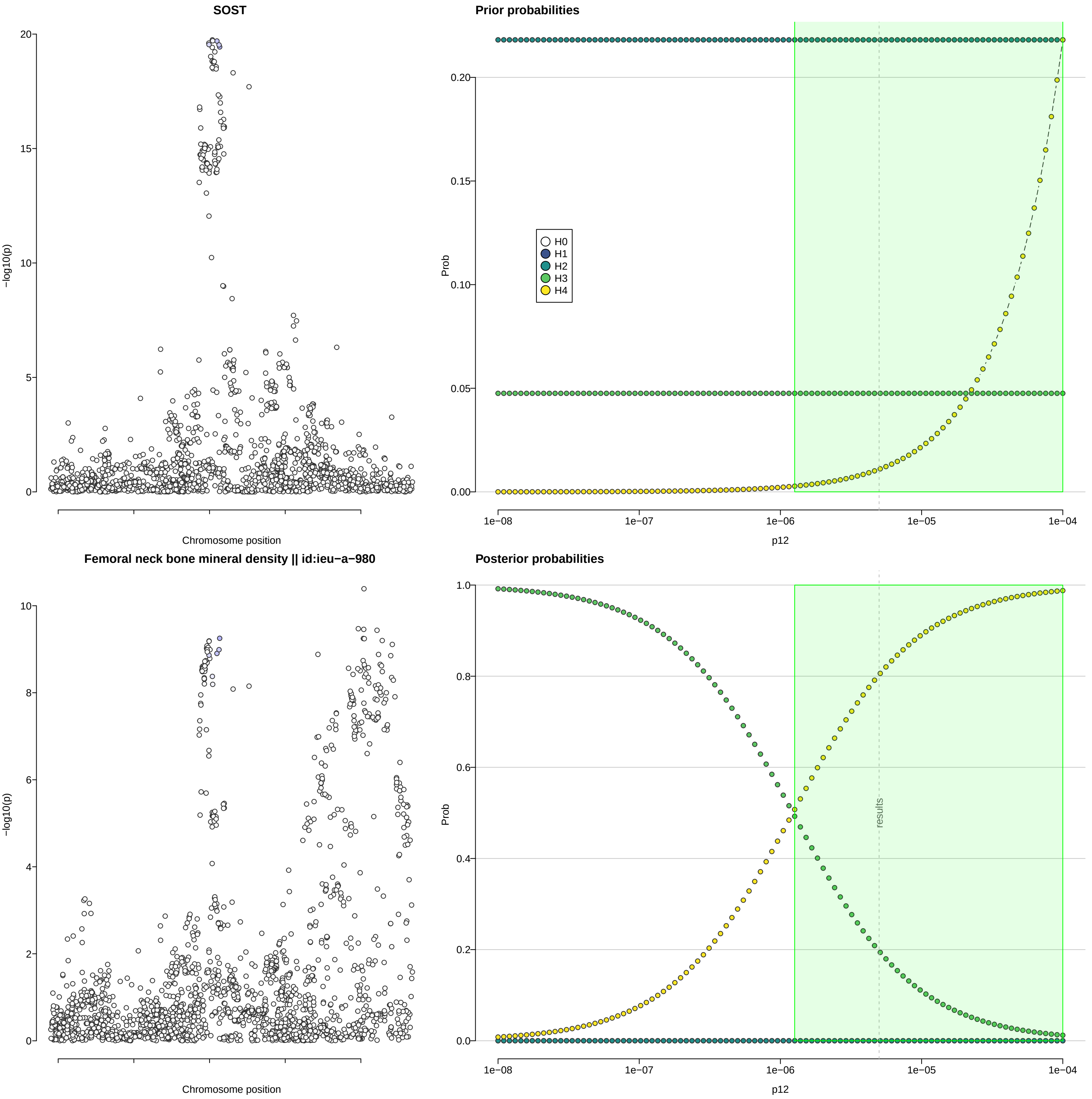

Supplementary Figure S10I. Locus zoom and prior sensitivity plots from colocalization analysis for Mendelian randomisation association of SOST with femoral neck bone mineral density

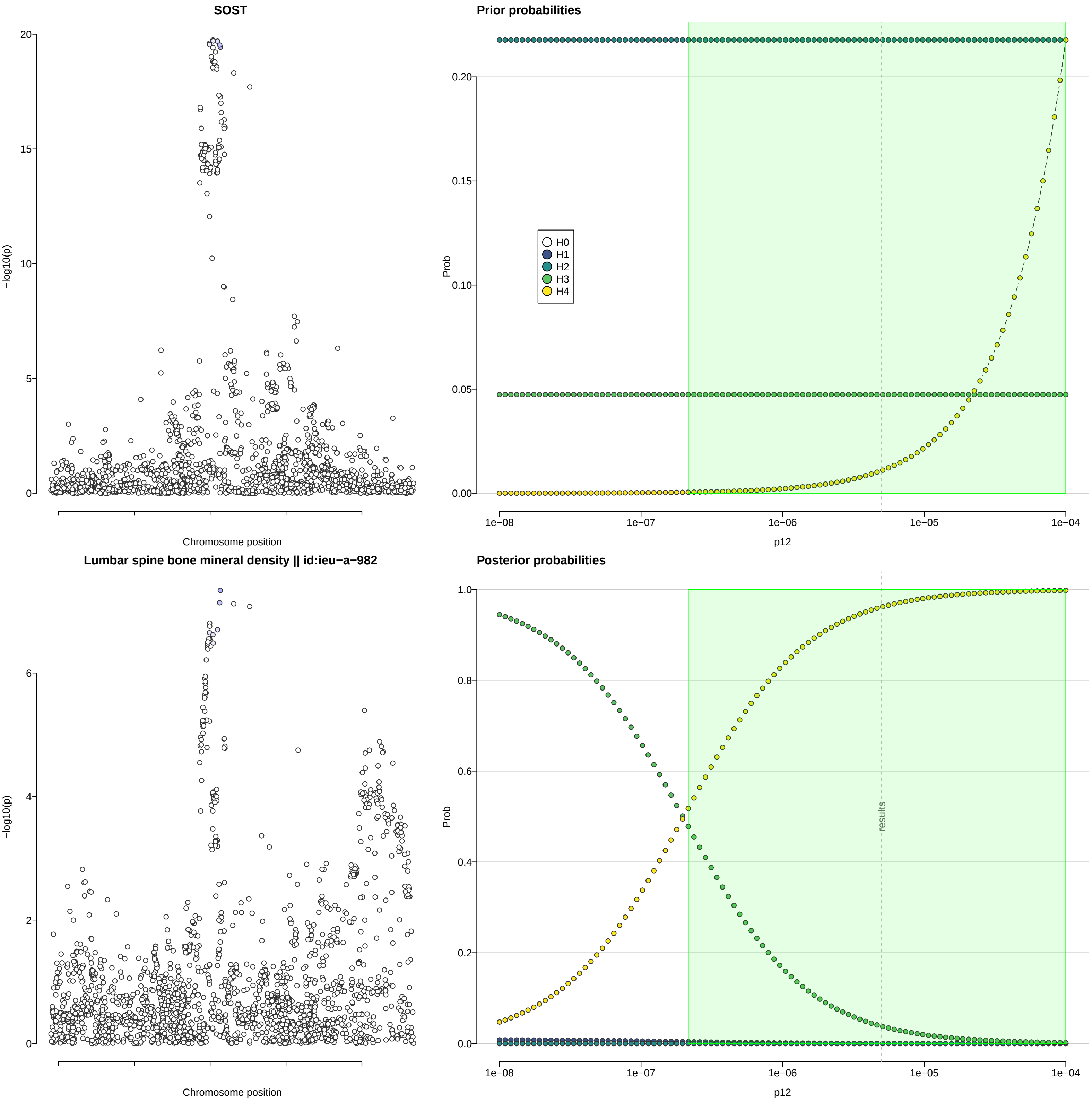

Supplementary Figure S10m. Locus zoom and prior sensitivity plots from colocalization analysis for Mendelian randomisation association of SOST with lumbar spine bone mineral density

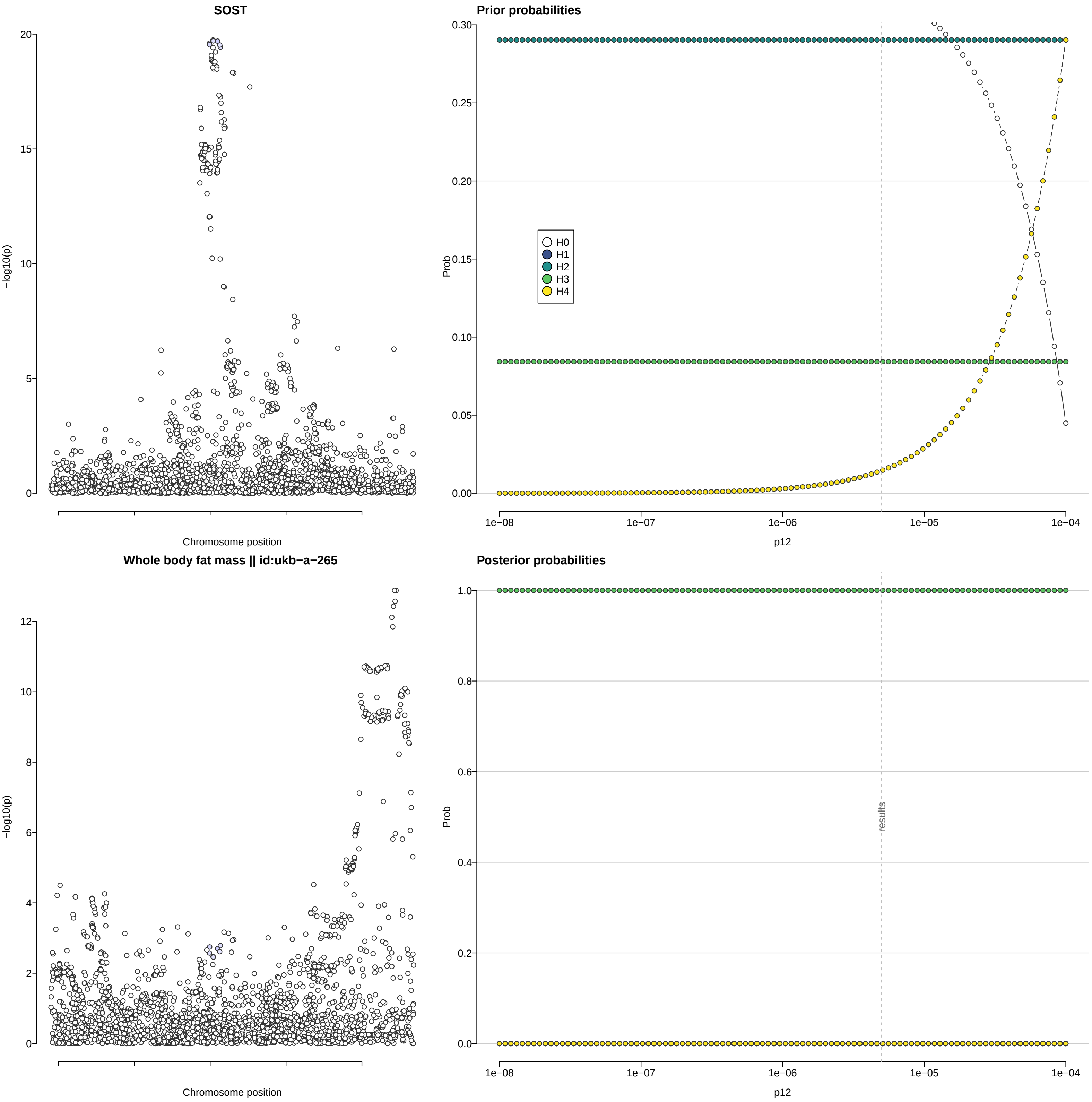

Supplementary Figure S10n. Locus zoom and prior sensitivity plots from colocalization analysis for Mendelian randomisation association of SOST with whole body fat mass

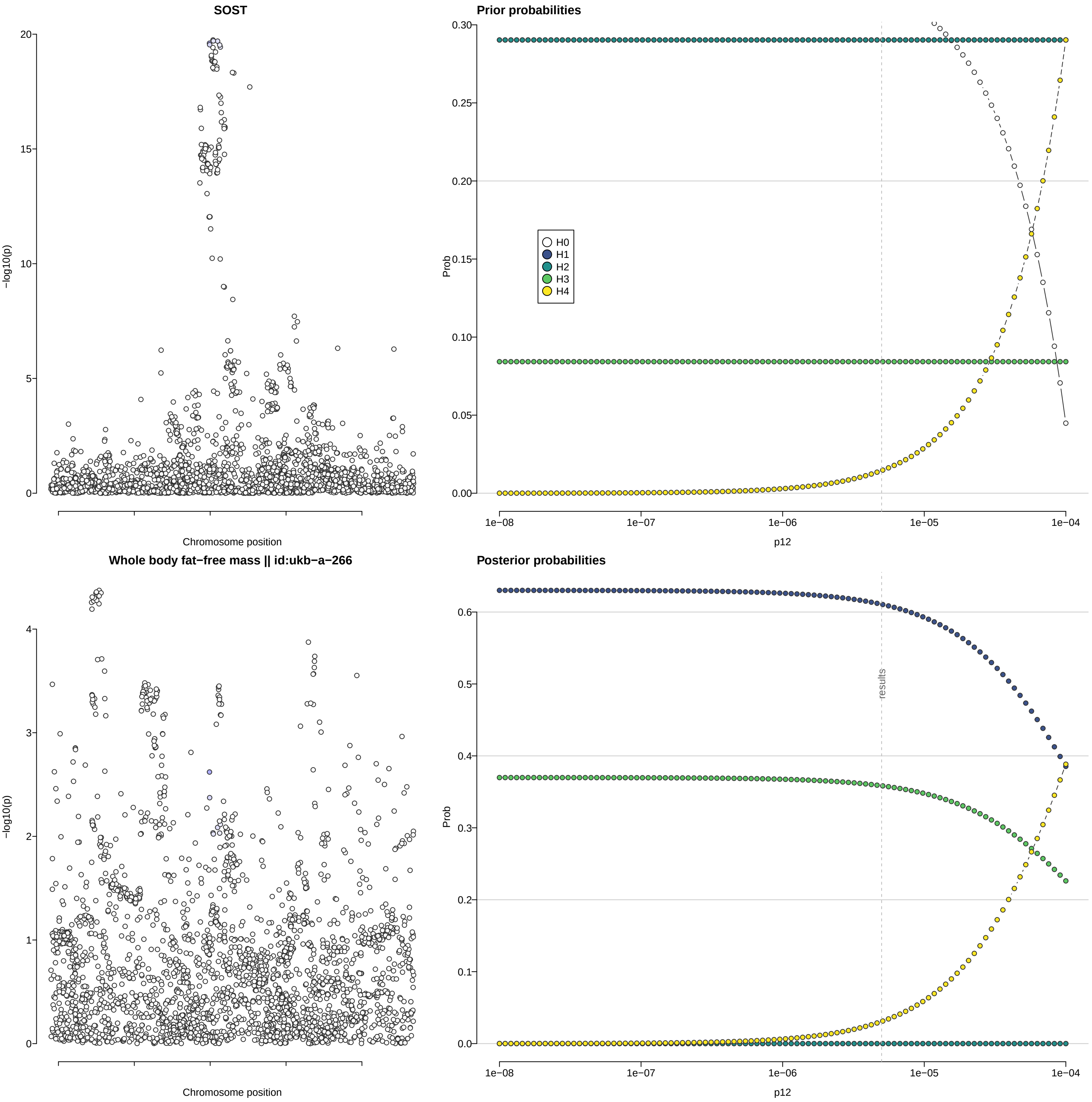

Supplementary Figure S10o. Locus zoom and prior sensitivity plots from colocalization analysis for Mendelian randomisation association of SOST with whole body fat-free mass

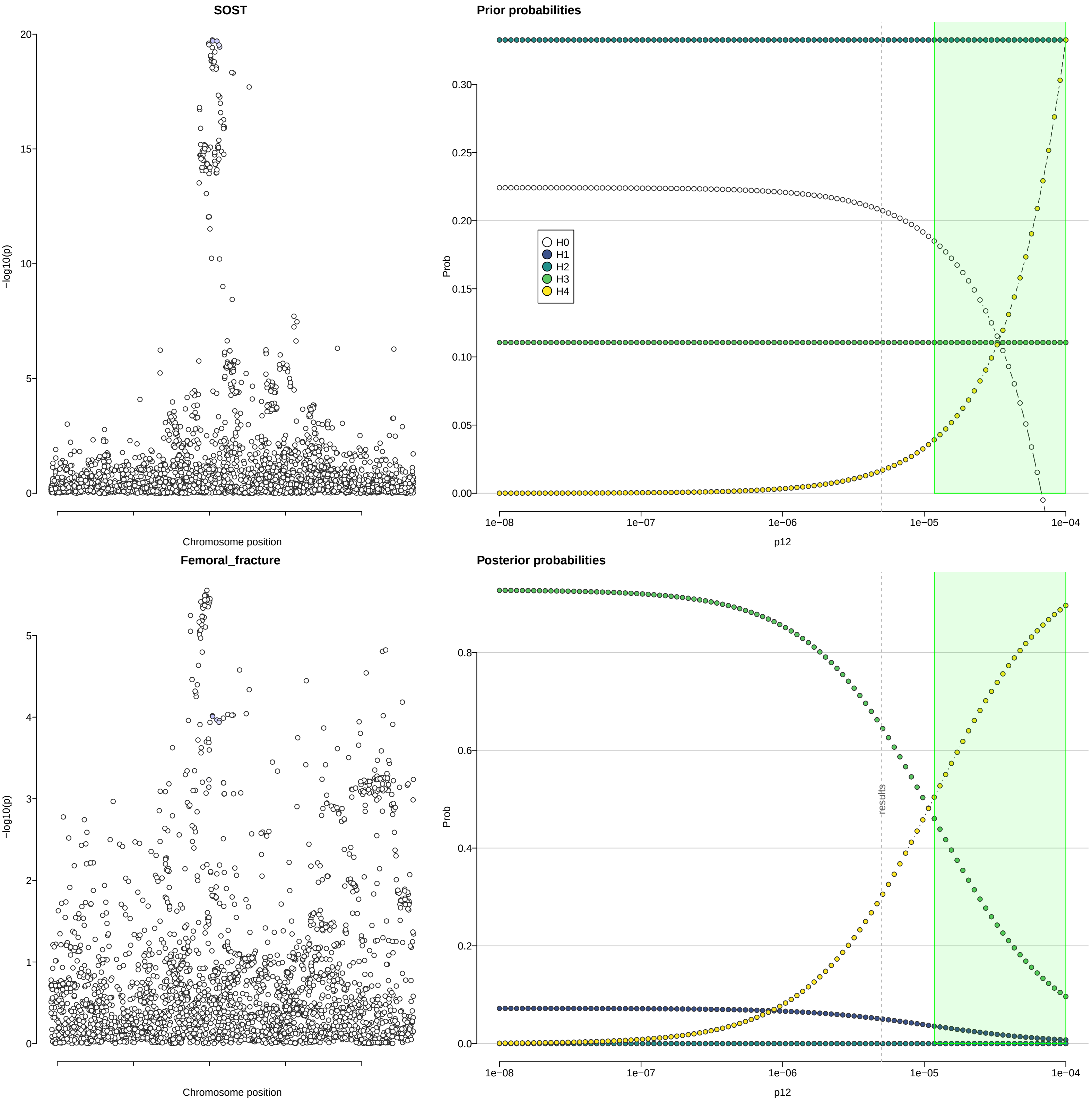

Supplementary Figure S10p. Locus zoom and prior sensitivity plots from colocalization analysis for Mendelian randomisation association of SOST with femoral fracture

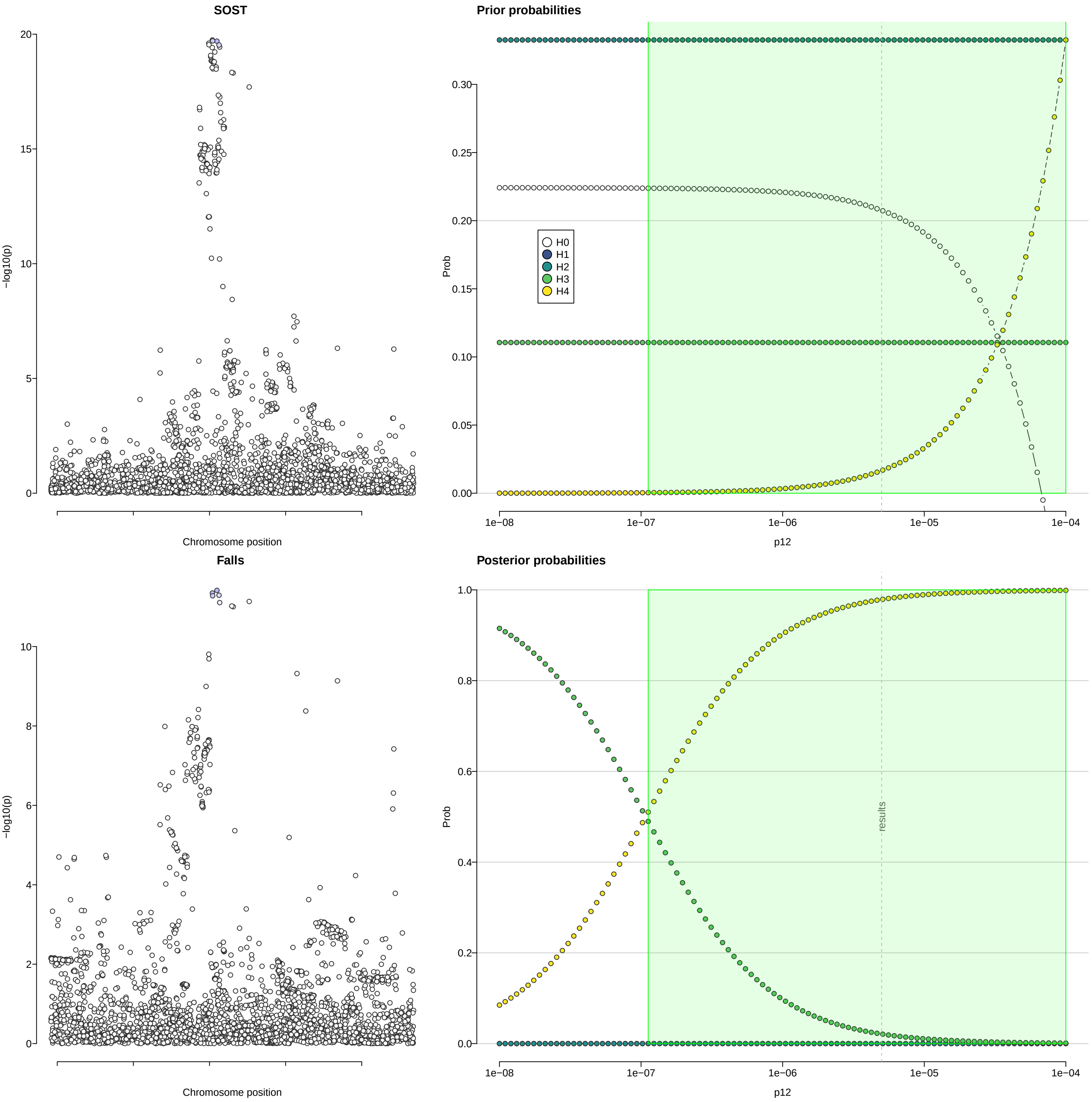

Supplementary Figure S10q. Locus zoom and prior sensitivity plots from colocalization analysis for Mendelian randomisation association of SOST with falls

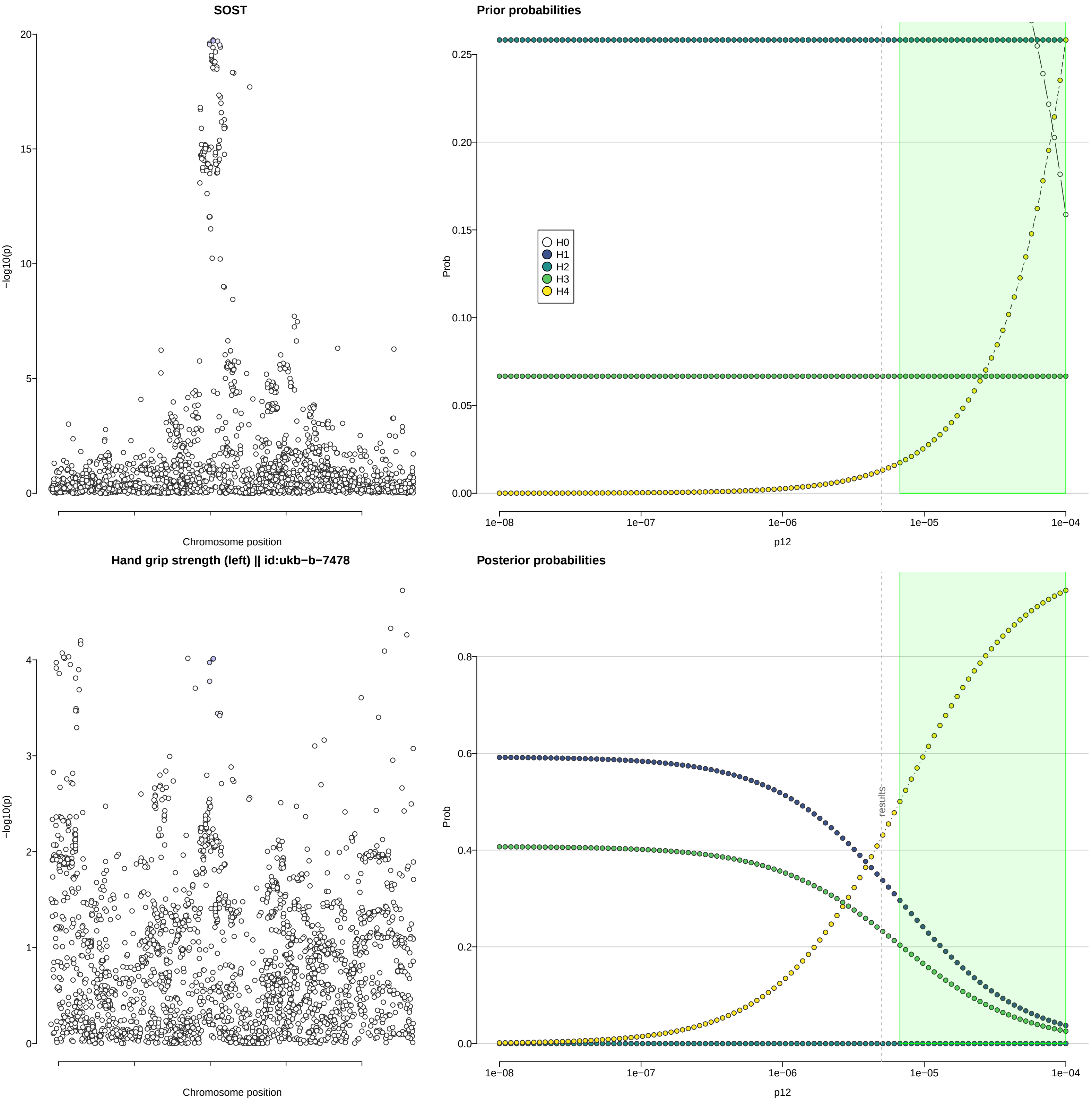

Supplementary Figure S10r. Locus zoom and prior sensitivity plots from colocalization analysis for Mendelian randomisation association of SOST with hand grip strength (left)

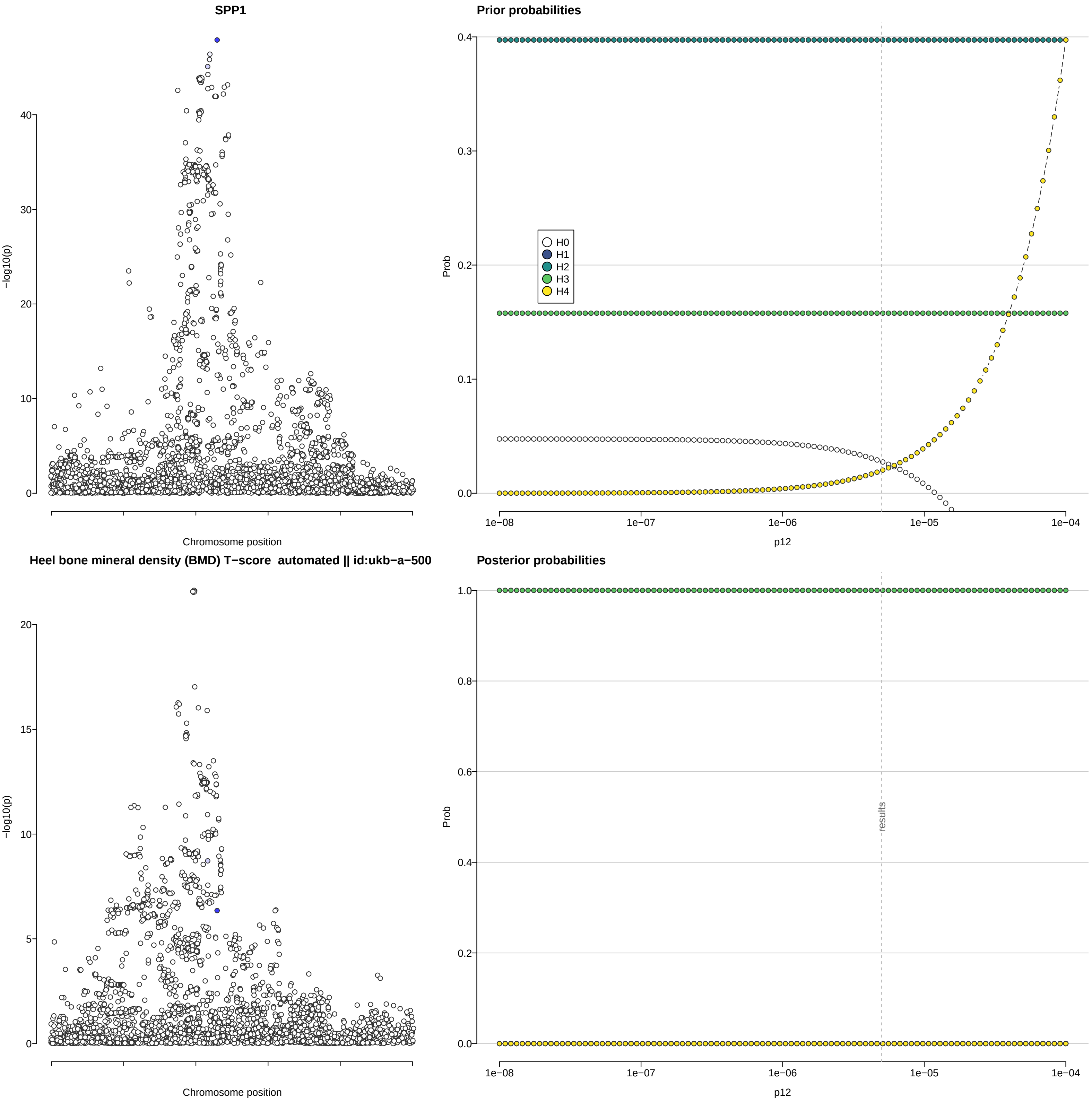

Supplementary Figure S10s. Locus zoom and prior sensitivity plots from colocalization analysis for Mendelian randomisation association of SOST with heel bone mineral density (BMD) T-score automated

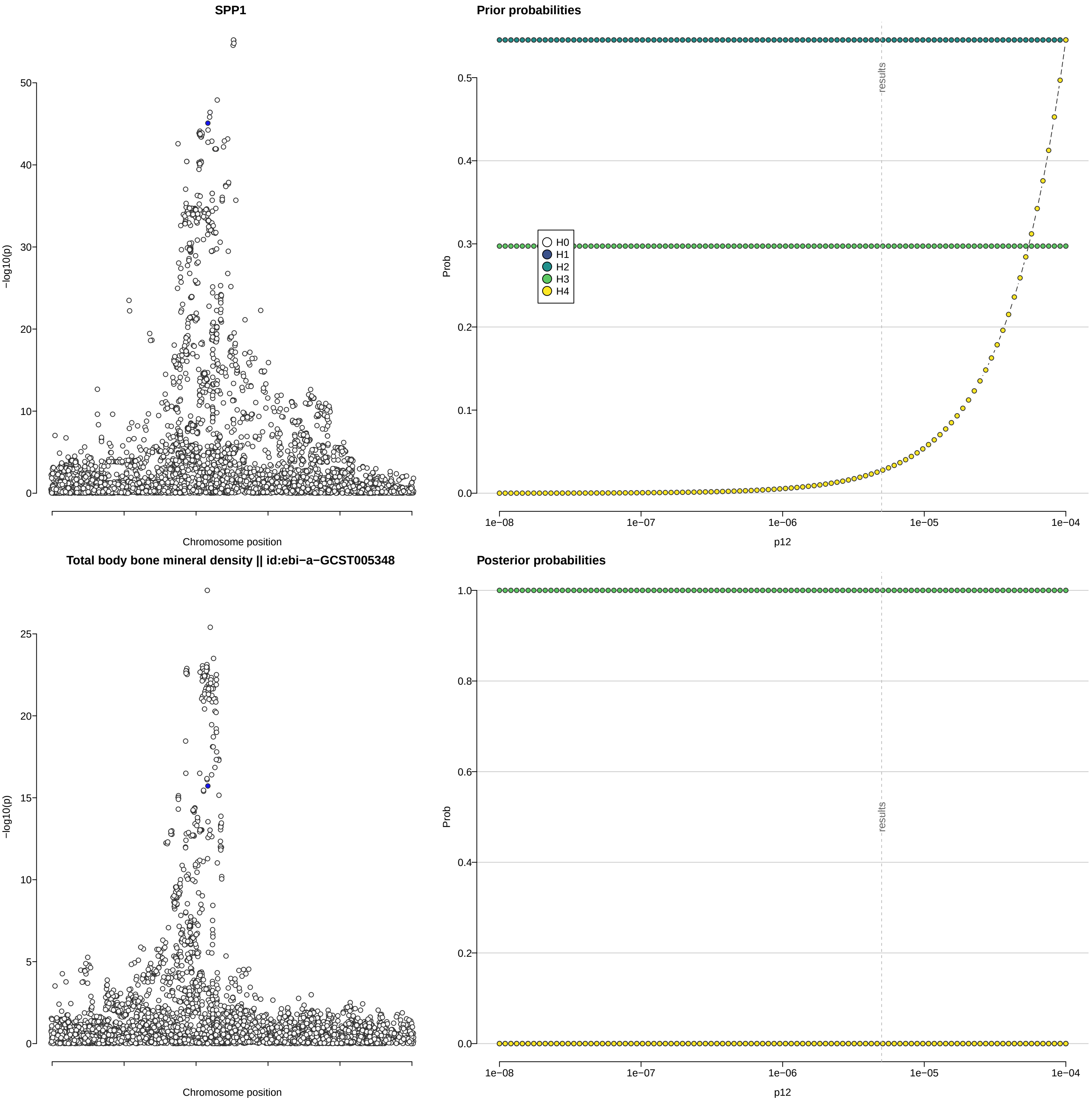

Supplementary Figure S10t. Locus zoom and prior sensitivity plots from colocalization analysis for Mendelian randomisation association of SPP1 with total body bone mineral density

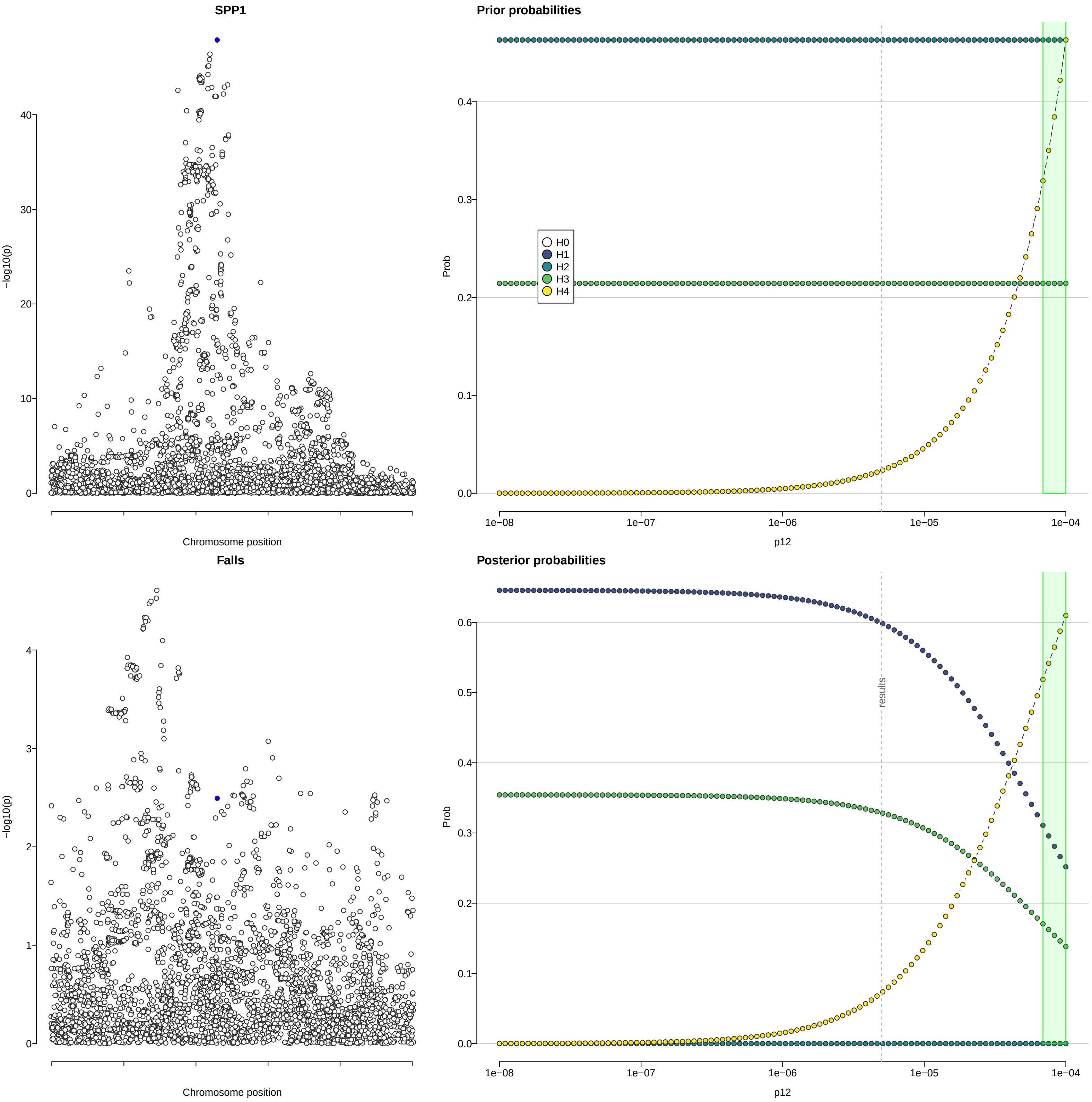

Supplementary Figure S10u. Locus zoom and prior sensitivity plots from colocalization analysis for Mendelian randomisation association of SPP1 with falls

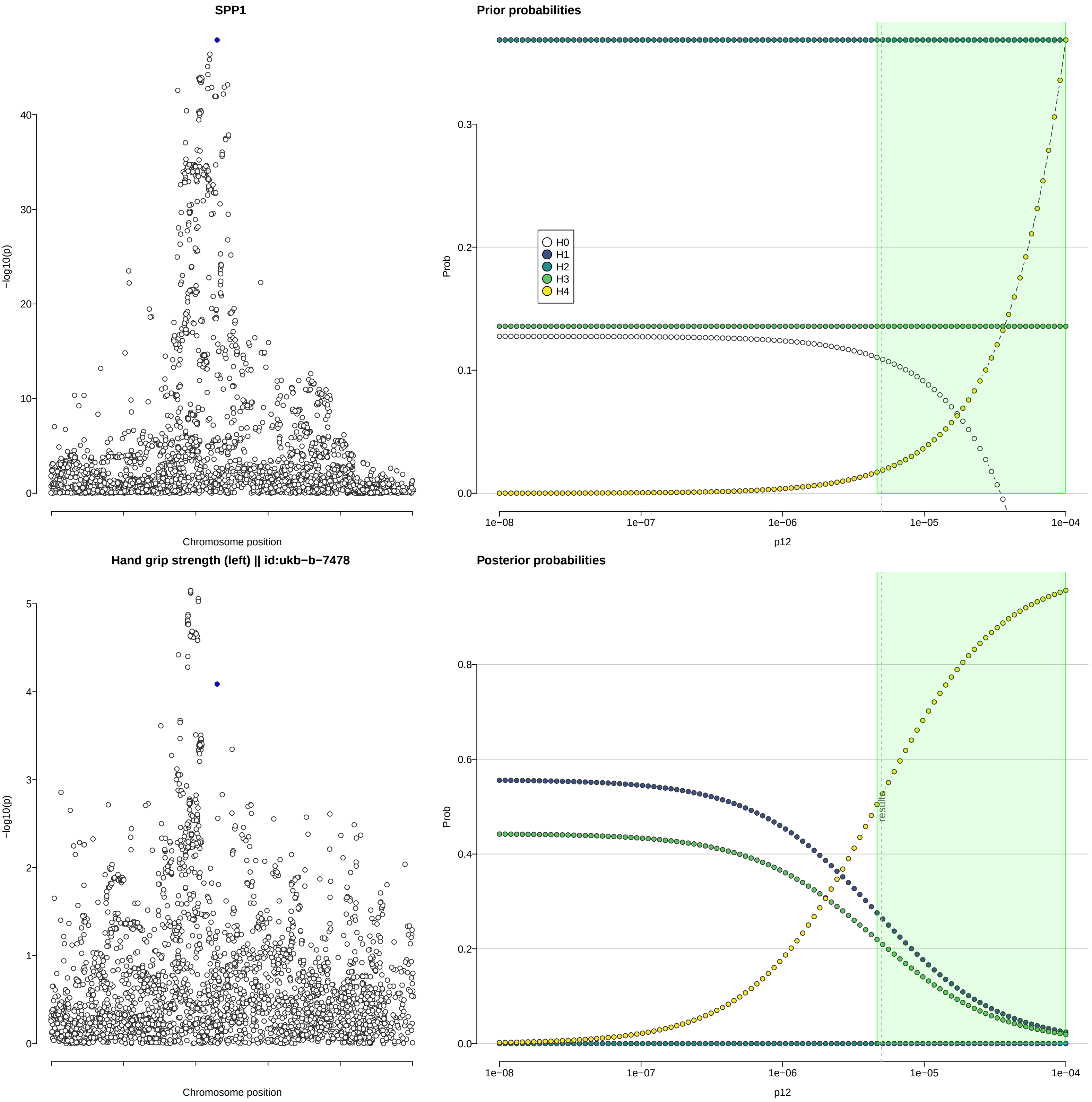

Supplementary Figure S10v. Locus zoom and prior sensitivity plots from colocalization analysis for Mendelian randomisation association of SPP1 with hand grip strength (left)

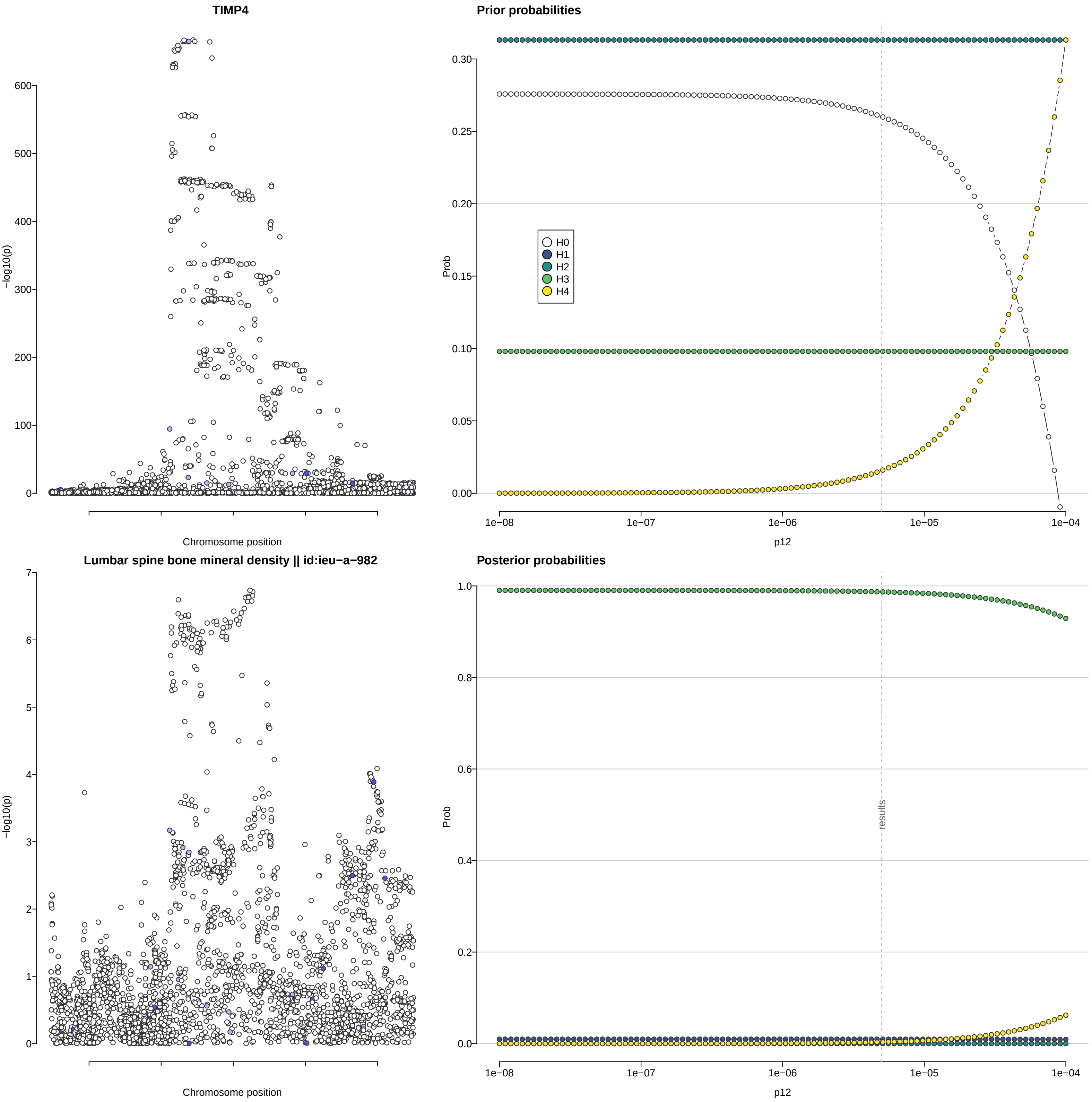

Supplementary Figure S10w. Locus zoom and prior sensitivity plots from colocalization analysis for Mendelian randomisation association of TIMP4 with lumbar spine bone mineral density

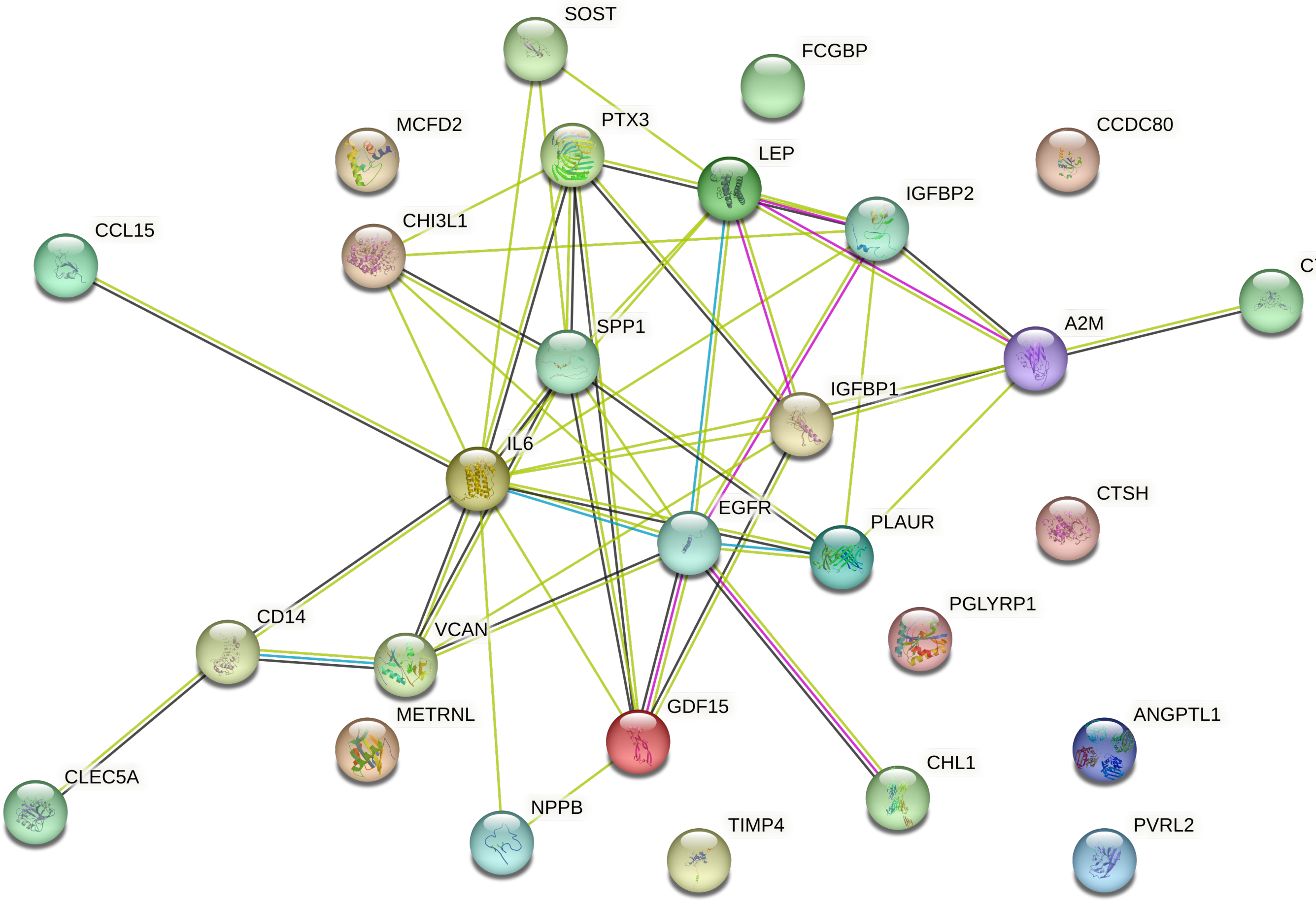

Supplementary Figure S11. The STRING-identified relations between our 24 fracture-related proteins and five proteins (CD14, CHL1, C7, PZP, and FCGBP) were previously discovered to be related to both bone loss and hip fracture.[117] STRING is the acronym for Search Tool for the Retrieval of Interacting Genes/Proteins) database (http://string-db.org).
